# Acute loss of *TET* function results in aggressive myeloid cancer in mice

**DOI:** 10.1038/ncomms10071

**Published:** 2015-11-26

**Authors:** Jungeun An, Edahí González-Avalos, Ashu Chawla, Mira Jeong, Isaac F. López-Moyado, Wei Li, Margaret A. Goodell, Lukas Chavez, Myunggon Ko, Anjana Rao

**Affiliations:** 1 Division of Signaling and Gene Expression, La Jolla Institute for Allergy and Immunology, La Jolla, California 92037, USA; 2 Stem Cells and Regenerative Medicine Center, Department of Pediatrics and Molecular and Human Genetics, Baylor College of Medicine, One Baylor Plaza, Houston, Texas 77030, USA; 3 Dan L. Duncan Cancer Center and Department of Molecular and Cellular Biology, Baylor College of Medicine, Houston, Texas 77030, USA; 4 Computational Oncoepigenomics Group, Division of Pediatric Neurooncology, German Cancer Research Center (DKFZ), Im Neuenheimer Feld 280, 69120 Heidelberg, Germany; 5 School of Life Sciences, Ulsan National Institute of Science and Technology, UNIST-gil 50, Ulju-gun, Ulsan 689-798, Republic of Korea; 6 Department of Pharmacology and Moores Cancer Center, University of California at San Diego, La Jolla, California 92093, USA; 7 Sanford Consortium for Regenerative Medicine, La Jolla, California 92037, USA

## Abstract

TET-family dioxygenases oxidize 5-methylcytosine (5mC) in DNA, and exert tumour suppressor activity in many types of cancers. Even in the absence of TET coding region mutations, TET loss-of-function is strongly associated with cancer. Here we show that acute elimination of TET function induces the rapid development of an aggressive, fully-penetrant and cell-autonomous myeloid leukaemia in mice, pointing to a causative role for TET loss-of-function in this myeloid malignancy. Phenotypic and transcriptional profiling shows aberrant differentiation of haematopoietic stem/progenitor cells, impaired erythroid and lymphoid differentiation and strong skewing to the myeloid lineage, with only a mild relation to changes in DNA modification. We also observe progressive accumulation of phospho-H2AX and strong impairment of DNA damage repair pathways, suggesting a key role for TET proteins in maintaining genome integrity.

## Abstract (web summary)

TET dioxygenases are known to have tumour suppressor activity. Here, An *et al*. show that Tet2/Tet3 double conditional mutant mice develop aggressive myeloid leukaemia, and suggest that rather than increased DNA methylation, aberrant gene expression and defects in DNA damage response and repair are the major drivers of myeloid leukaemogenesis upon TET loss-of-function.

## Graphical abstract

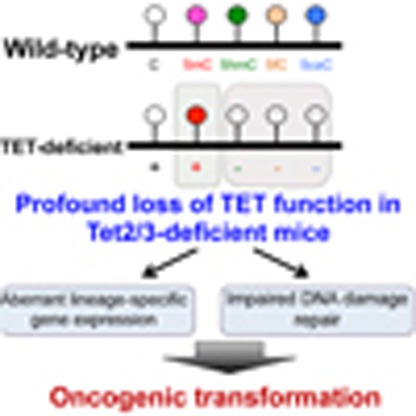

Enzymes of the TET (ten-eleven translocation) family are dioxygenases that convert 5-methylcytosine (5mC) to 5-hydroxymethylcytosine (5hmC) and the further oxidation products 5-formylcytosine (5fC) and 5-carboxylcytosine (5caC)^1^,^2^,^3^,^4^. Together these oxidized methylcytosines (oxi-mC) facilitate DNA demethylation and also function as epigenetic marks^5^,^6^,^7^. Loss-of-function mutations in *TET2* are associated with diverse myeloid and lymphoid malignancies in humans^8^,^9^,^10^, but diminished TET expression or activity are also prominent features of numerous other cancers including melanoma and glioblastoma; moreover, low TET1 levels in breast and other cancers have been shown to correlate with advanced disease, metastases and poor patient survival (reviewed in refs 11, 12). Nevertheless, the molecular connections between TET loss-of-function and oncogenic transformation remain to be defined.

In humans, *TET2* is recurrently deleted or mutated in a wide range of myeloid malignancies including myelodysplastic syndromes, myeloproliferative neoplasms, chronic myelomonocytic leukaemia, acute myeloid leukaemia and secondary acute myeloid leukaemia, as well as in T-cell lymphomas including angioimmunoblastic T-cell lymphoma and peripheral T-cell lymphoma-not otherwise specified^8^,^9^,^10^,^13^,^14^. The *TET2* mutations observed in these conditions are inactivating loss-of-function mutations that impair 5mC oxidation and are associated with decreased genomic 5hmC levels^2^; however, the development of full-blown malignancy requires a second hit^11^,^12^.

To model this phenomenon, we and others have generated and studied *Tet*-deficient mice^8^,^15^,^16^,^17^,^18^. Tet2 and Tet3 are both highly expressed in murine haematopoietic stem/progenitor cells (HSPCs)^2^, and their individual deletion in mice results in aberrant haematopoiesis, characterized by expansion of HSPCs with enhanced self-renewal capacity, augmented haematopoietic repopulation and preferential differentiation towards myeloid lineages^8^,^12^,^15^,^16^,^17^. Certain strains of *Tet2*-deficient mice develop a chronic myelomonocytic leukaemia-like disease, but with long latency and low penetrance^8^,^15^,^16^. Similarly, germline Tet1 deficiency in mice is associated with lymphocytosis and the late development of a poorly penetrant B-cell lymphoma^18^. Together these results indicate that loss of a single TET protein is not sufficient to promote malignancy efficiently.

In this study, to explore the relation between TET loss-of-function and cancer, we generated mice homozygous for a germline deletion of *Tet2* and a conditional allele of *Tet3*. Our goal was not to model a human disease, since *TET3*-coding region mutations are relatively rare in humans^19^. Rather, we wished to examine the relation of TET loss-of-function to oncogenesis, using a mouse model in which a profound loss of TET function could be acutely induced *in vivo*. We show here that *Tet2*^−/−^ mice acutely deleted for *Tet3* displayed a rapid, progressive leukocytosis with neutrophilia, monocytosis, thrombocytopenia and severe anaemia, which developed within a few weeks into a highly aggressive myeloid leukaemia in 100% of the mice. Transcriptional profiling revealed aberrant lineage priming^20^ in HSPC, coupled to impaired erythroid and lymphoid differentiation and marked skewing towards the myeloid lineage. These changes in gene transcription were not strongly linked to changes in DNA methylation. Bone marrow chimera and splenocyte transfer experiments indicated that the myeloid leukaemia was induced in a cell-autonomous manner and was transplantable to secondary recipient mice. Myeloid progenitors and mature myeloid-lineage cells acutely deleted for TET function progressively accumulated DNA damage and showed strong impairment of DNA damage responses and DNA break repair. Our data indicate that TET loss-of-function accelerates myeloid leukaemogenesis, through mechanisms that involve lineage dysregulation, uncontrolled expansion and genomic instability in differentiating cells.

## Results

### Acute loss of TET function results in myeloid leukaemia

**Figure 1:**
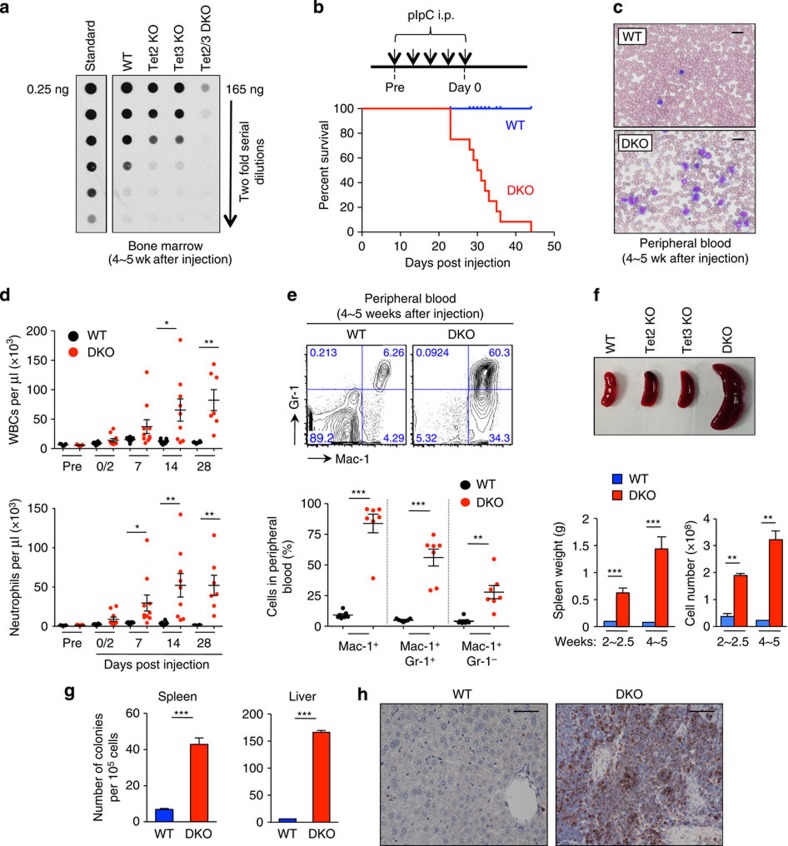
Acute deletion of *Tet3* in *Tet2*-deficient mice results in myeloid leukaemia. (**a**) Quantification of 5hmC levels in *Tet2/3* DKO bone marrow 4 weeks after pIpC injection by anti-CMS dot blot. (**b**) Kaplan–Meier curve representing percent survival of WT (*n*=23) and DKO (*n*=14) mice over time after pIpC injection. No lethality was observed for WT, *Tet2*-deficient (*n*=4) and *Tet3*-deficient (*n*=6) mice during this period. Strategy for conditional deletion of *Tet3* in adult mice is shown above. For exact genotypes, see Methods. (**c**) May–Grünwald–Giemsa-stained peripheral blood smears, 4 weeks after pIpC administration (*n*=7). Scale bar, 20 μm. (**d**) Time-course analysis of peripheral blood cell counts after acute deletion of *Tet3* in *Tet2*-deficient mice (means±s.e.m.). DKO mice developed progressive leukocytosis with neutrophilia (*n*=7∼10 per genotype at each time point examined). *P<0.05, **P<0.005 (Student's *t*-test). Also see Supplementary Fig. 2. (**e**) Expansion of myeloid-lineage cells in DKO mice. Flow cytometric analysis of myeloid cells in the peripheral blood of WT (*n*=7) or DKO (*n*=7) mice was performed at 4 weeks following pIpC injection. Bottom, summary of results (means±s.e.m.). For kinetic analyses of myeloid, B- and T-cell lineages over the entire time course, see Supplementary Fig. 3. **P<0.005, ***P<0.0005 (Student's *t*-test). (**f**) Enlargement of spleens in *Tet2/3* DKO mice at 2∼2.5 weeks following pIpC injection. Top—representative photographs. Bottom—spleen weights and cellularity (*n*=7∼13 and 4∼6 per genotype) at 2–2.5 or 4–5 weeks after pIpC injection (means±s.e.m.). **P<0.005, ***P<0.0005 (Student's *t*-test). (**g**) Extramedullary haematopoiesis in spleen and liver of *Tet2/3* DKO mice. Cells were harvested from the spleen (left) or liver (right) of WT and diseased *Tet2/3* DKO mice and 10^5^ nucleated cells were plated in methylcellulose medium. Colony-forming units were assessed by counting cell colonies 7 days after plating. ***P<0.0005 (Student's *t*-test) (**h**) Myeloperoxidase staining of livers at 4 weeks after pIpC injection (× 40 magnification). For liver weight and histological analysis data, see Supplementary Fig. 4a–d. Scale bar, 60 μm.

To diminish TET function profoundly in adult mice, we first set up an inducible system whereby *Tet3* could be acutely deleted in haematopoietic precursor cells in the context of a germline deletion of *Tet2* (*Tet2*^−/−^ *Tet3*^*fl/fl*^ *Mx1-Cre*^*+*^ mice)^12^,^17^. The mice were injected five times with polyinosine–polycytidine (pIpC) over a 10-day period, a regimen that induces Cre recombinase expressed under control of the interferon-α-inducible *Mx1* promoter^21^. After 2 weeks, we observed a complete loss of *Tet3* messenger RNA expression in several haematopoietic cell types, with no compensatory upregulation of *Tet1* (Supplementary Fig. 1a). Loss of TET function was monitored at 2 and 4 weeks after pIpC injection by anti-cytosine-5-methylenesulfonate dot blot of bisulfite-treated genomic DNA^2^. Ablation of either *Tet2* or *Tet3* led to a modest (approximately twofold) decrease in 5hmC levels in the bone marrow and spleen, but deletion of both genes led to an almost complete loss of 5hmC (Fig. 1a; Supplementary Fig. 1b–e). Thus Tet2 and Tet3 are the main enzymes that catalyse 5hmC production in cells of the haematopoietic system.

Between 3 and 7 weeks after pIpC injection, 100% of injected *Tet2/3* DKO mice became very sick and were euthanized, whereas all wild-type (WT) and singly-deficient mice (*Tet2*^−/−^ or *Tet3*^*fl/fl*^ *Mx1*-*Cre*^*+*^) survived (Fig. 1b; Supplementary Table 1). Examination of peripheral blood showed that DKO mice developed progressive leukocytosis, mainly due to expansion of myeloid-lineage cells including neutrophils, monocytes, eosinophils and basophils, with concomitant development of profound anaemia and thrombocytopenia (Fig. 1c,d; Supplementary Fig. 2). Flow cytometry confirmed progressive amplification of myeloid-lineage cells in the peripheral blood of DKO mice: Gr-1^+^ Mac-1^+^ granulocytes increased within 2 days of the last pIpC injection, with Gr-1^−^ Mac-1^+^ monocytes expanding at somewhat later times (Fig. 1e; Supplementary Fig. 3a–c), and the percentages of B and T lymphoid cells showing a gradual decrease (Supplementary Fig. 3d,e).

In all *Tet2/3* DKO mice, myeloid expansion was accompanied by massive, progressive splenomegaly and hepatomegaly (Fig. 1f; Supplementary Fig. 4a). This was at least partly due to extramedullary haematopoiesis, since DKO spleen and liver cells displayed an increased ability to form haematopoietic colonies *in vitro* (Fig. 1g). We also observed disruption of normal splenic architecture and pronounced infiltration of liver, lung, bone marrow and spleen with haematopoietic cells in *Tet2/3* DKO mice (Supplementary Figs 4 and 5). In the liver, the infiltrating cells were myeloperoxidase positive (Fig. 1h; Supplementary Fig. 4d) and were identified as Gr-1^+^/Mac-1^+^ myeloid-lineage cells by flow cytometry (Supplementary Fig. 6). Staining of DKO bone marrow aspirates revealed a preponderance of myeloid cells of uniform appearance (myeloblasts, granulocytes and monocytes), whereas WT bone marrow showed the normal heterogeneous pattern of trilineage maturation (Supplementary Fig. 5e).

**Figure 2:**
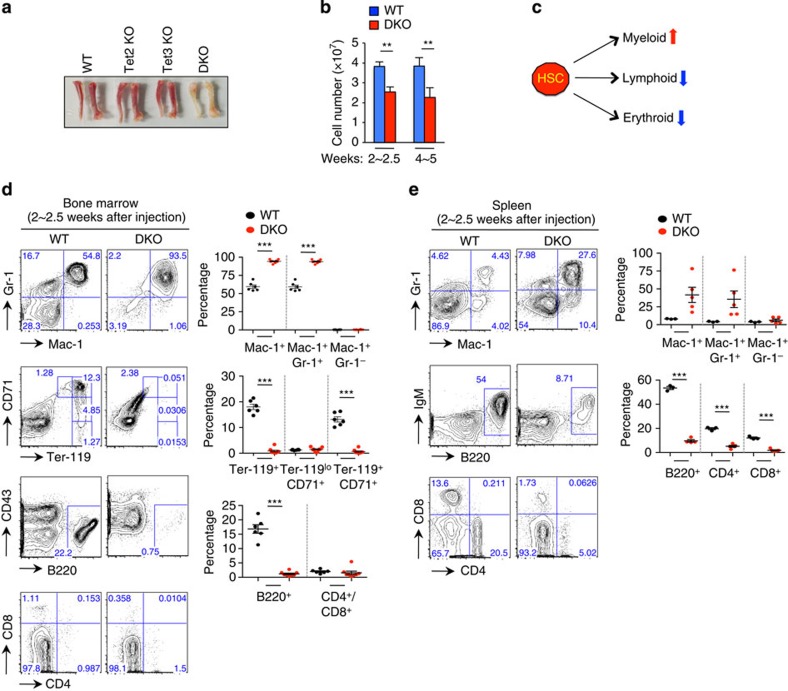
Combined loss of Tet2 and Tet3 results in expansion of myeloid cells with impaired lymphoid and erythroid differentiation. (**a**) Representative photographs of femurs and tibiae at 4 weeks following pIpC injection. Tet2 KO, *Tet2*^−/−^ *Tet3*^*fl/fl*^ *Mx1-Cre*^−^ or *Tet2*^−/−^ *Mx1-Cre*^+^; Tet3 KO, *Tet3*^*fl/fl*^ *Mx1-Cre*^+^. (**b**) Total cellularity of bone marrow was assessed at the indicated time points after pIpC injection (*n*=8∼11 per genotype at each time point). Means±s.e.m. are shown. **P<0.005 (Student's *t*-test). (**c**) Schematic representation of myeloid expansion and impaired lymphoid and erythroid development upon loss of *Tet2* and *Tet3*. (**d**,**e**) Haematopoietic cell development upon simultaneous deletion of *Tet2* and *Tet3*. Flow cytometry was performed to assess myeloid (Gr-1/Mac-1), erythroid (CD71/Ter-119) and lymphoid (B220/CD4/CD8) cell populations in the bone marrow (**d**, *n*=8 per each genotype) and spleen (**e**, *n*=6 per each genotype) of WT or DKO mice. Representative flow cytometry plots (left) and summary graphs (right) at 2∼2.5 weeks after pIpC injection are shown. For results at 4∼5 weeks, see Supplementary Figs 7 and 8. Means±s.e.m. are shown. **P<0.005, ***P<0.0005 (Student's *t*-test).

As expected from our examination of peripheral blood (Supplementary Fig. 2), *Tet2/3* DKO mice developed profound anaemia and lymphopenia. Bones isolated from *Tet2/3* DKO mice (but not Tet2 KO or Tet3 KO mice) were pale and their bone marrow contained reduced numbers of cells (Fig. 2a,b). At both early (2–2.5 weeks) and late (4–5 weeks) times after pIpC injection, we observed a substantial increase in the frequency of Gr-1^+^/ Mac-1^+^ myeloid-lineage cells in liver, bone marrow and spleen, a marked decrease in the frequency of Ter-119^+^ erythroid cells and B220^+^ B cells in bone marrow, and a striking decrease in the fraction of B220^+^ B cells, CD4^+^ and CD8^+^ T cells in liver and spleen of *Tet2/3* DKO mice compared with WT (Fig. 2c–e; Supplementary Figs 6–8).

Taken together, these results indicate that Tet2 and Tet3 play redundant roles in the haematopoietic system and can compensate for one another to suppress malignant transformation. Acute loss of Tet3 in the context of a germline *Tet2* deletion results in rapid and progressive expansion of myeloid-lineage cells with lymphoid and erythroid dysplasia, ultimately causing a lethal myeloid neoplasia. On the basis of the criteria described in the Bethesda proposal for classification of nonlymphoid haematopoietic neoplasms in mice^22^, these phenotypes are characteristic of myeloproliferative disorders-like myeloid leukaemia.

### Profound loss of TET function affects HSPC

**Figure 3:**
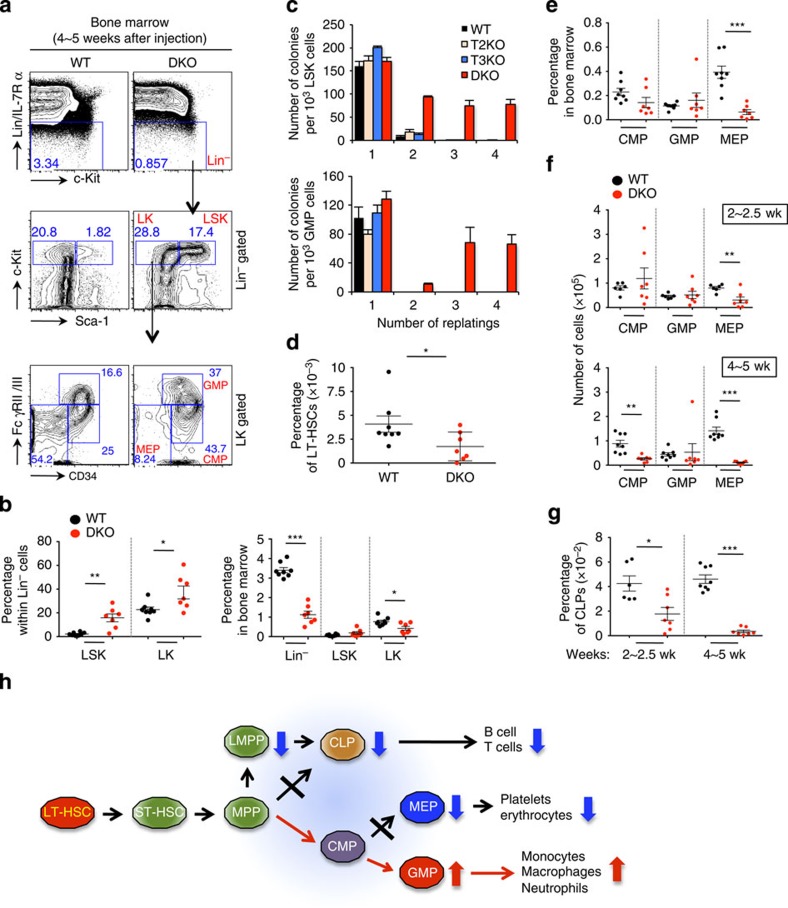
Altered development of haematopoietic stem and progenitor cells in *Tet2/3* DKO mice. (**a**) Representative flow cytometric analysis of LSK (Lin^−^ c-Kit^+^ Sca1^+^) and LK (Lin^−^ c-Kit^+^ Sca1^−^) populations in the bone marrow of WT and *Tet2/3* DKO mice 4 weeks after pIpC injection. Myeloid progenitors (CMP, common myeloid progenitors; GMP, granulocyte–monocyte progenitors; MEP, megakaryocyte-erythroid progenitors) were further analysed based on CD34 and FcγRII/III expression within LK populations. (**b**) Percentages of LSK and LK cells within Lin^−^ (left) or total bone marrow (right) shown in **a** (*n*=7∼8) (means±s.e.m.). *P<0.05, **P<0.005, ***P<0.0005 (Student's *t*-test). For percentages at 2∼2.5 weeks and absolute cell numbers see Supplementary Fig. 9a,b. (**c**) Combined deficiency of Tet2 and Tet3 leads to increased serial replating capacity *in vitro*. WT, Tet2-deficient (T2KO), Tet3-deficient (T3KO) and DKO mice were treated with pIpC, and colony-forming unit assays were performed with LSK (upper panel) or GMP (lower panel) cells. DKO, but not WT, T2KO or T3KO LSK and GMP cells can be serially replated in methylcellulose medium. For results after acute deletion of *Tet3 in vitro* in *Tet2*^−/−^ *Tet3*^*fl/fl*^ *ERT2-Cre*^+^ mice, see Supplementary Fig. 9c–f. (**d**) Percentage of long-term haematopoietic stem cells (LT-HSC, LSK CD150^+^ CD48^−^). For representative flow cytometric analysis, see Supplementary Fig. 10a. (**e**) Percentages of myeloid progenitor cell subsets shown in (**a**) (*n*=7∼8) (means±s.e.m.). ***P<0.0005 (Student's *t*-test). For results at 2∼2.5 weeks and absolute cell numbers, see Supplementary Fig. 11a,b. (**f**) Absolute numbers of myeloid progenitor cells in bone marrow of WT and *Tet2/3* DKO mice. Means±s.e.m. are shown. **P<0.005, ***P<0.0005 (Student's *t*-test). (**g**) Percentages of common lymphoid progenitors (CLP, Lin^−^ Flt3^+^ CD27^+^ IL-7Rα^+^) in the bone marrow at 2 or 4 weeks after pIpC injection (*n*=6∼8). Gating strategy and absolute cell numbers are shown in Supplementary Fig. 11c,d. Means±s.e.m. are shown. *P<0.05, ***P<0.0005 (Student's *t*-test). (**h**) Schematic representation of altered haematopoietic development in *Tet2/3* DKO mice.

We examined the impact of TET deficiency on haematopoietic stem cell (HSC) and progenitor compartments in the bone marrow. Loss of *Tet2* and *Tet3* led to a striking increase in the percentage of LSK cells within the lineage-negative (Lin^−^) population, but because the frequency of the Lin^−^ population was greatly reduced, the average number of LSK cells in total bone marrow was unchanged or slightly decreased (Fig. 3a,b; Supplementary Fig. 9a,b). Notably, DKO LSK and granulocyte–monocyte progenitor (GMP) cells, but not the corresponding WT, Tet2 KO or Tet3 KO cells, showed increased serial replating capability when cultured in methylcellulose *in vitro* (Fig. 3c; Supplementary Fig. 9f; for the flowchart of experiment and extent of *Tet3* deletion see Supplementary Fig. 9c–e). In previous studies, Tet2-deficient LSK cells displayed increased serial replating capacity compared with WT LSK cells^15^, but our data show that double-Tet2/Tet3 deficiency has an even stronger effect.

Our attempts to determine the impact of *Tet2*/*Tet3* deletion on HSCs were complicated by the fact that TET loss-of-function altered HSC marker expression (Supplementary Fig. 10a–c). The frequency and number of SLAM-marked long-term HSCs (LT-HSCs, defined as LSK CD150^+^ CD48^−^)^23^ were markedly decreased in DKO bone marrow (Fig. 3d; Supplementary Fig. 10a), but the percentage and numbers of LT-HSCs defined using different markers (LSK CD34^−^ Flt3^−^)^24^ showed a significant increase (Supplementary Fig. 10b,c). Using Flt3 and CD34 as markers, we documented a decrease in lymphoid-primed multipotent progenitor cells (LMPP, LSK CD34^+^ Flt3^hi^)^25^ (Supplementary Fig. 10b,c), and also showed that within the Flt3^lo/−^ LSK population, the ratio of CD34^−^ (LT-HSC) to CD34^+^ (ST-HSC) cells was only slightly decreased in DKO mice (Supplementary Fig. 10d).

To resolve the discrepancy between the two methods of identifying LT-HSC, we used Hoechst 33342 staining to define a ‘side population' that has been shown to be highly enriched for HSC^26^. Compared with WT mice, *Tet2/3* DKO mice showed a substantial decrease in the side population of HSCs (Supplementary Fig. 10e). Moreover, CD150^hi^ CD48^−^ cells within the LT-HSC population (CD34^−^ Flt3^−^ LSK) in the bone marrow, which are enriched for a dormant HSC population in mice^27^, were decreased in DKO compared with WT bone marrow (Supplementary Fig. 10f). Together these data suggest that Tet2 and Tet3 act to maintain the size of the HSC pool, but that the developmental transition from LT- to ST-HSCs is largely unaffected in the absence of Tet2 and Tet3. CD48 is among the first markers to be upregulated when HSCs transition out of the stem cell compartment^28^, hence the striking acquisition of CD48 on cells that otherwise resemble LT-HSCs suggests that HSC function is skewed towards myeloid progenitor cells in *Tet2/3 DKO* mice, consistent with the transcriptional profiles described below.

The developmental bias observed in *Tet2/3* DKO mice correlated strongly with alterations in the distribution of early lymphoid and myeloid progenitors in bone marrow (Fig. 3a). The percentages and absolute numbers of megakaryocyte-erythroid progenitors were substantially reduced in DKO bone marrow compared with WT; the mice displayed a substantial decrease in the numbers of common lymphoid progenitors (Lin^−^ Flt3^+^ CD27^+^ IL-7Rα^+^)^29^, and the size of the common myeloid progenitor compartment gradually decreased after pIpC injection, whereas the numbers of GMPs remained unchanged (Fig. 3a,e–g; Supplementary Fig. 11). In some mice, myeloid progenitor (LK) populations contained only GMPs (data not shown). These findings are consistent with the impaired erythropoiesis, decreased frequency and number of LMPP, impaired B and T lymphopoiesis and dominant myelopoiesis observed in *Tet2/3* DKO mice (Fig. 3h).

### Cell-autonomous leukaemia development in *Tet2/3 DKO* mice

**Figure 4:**
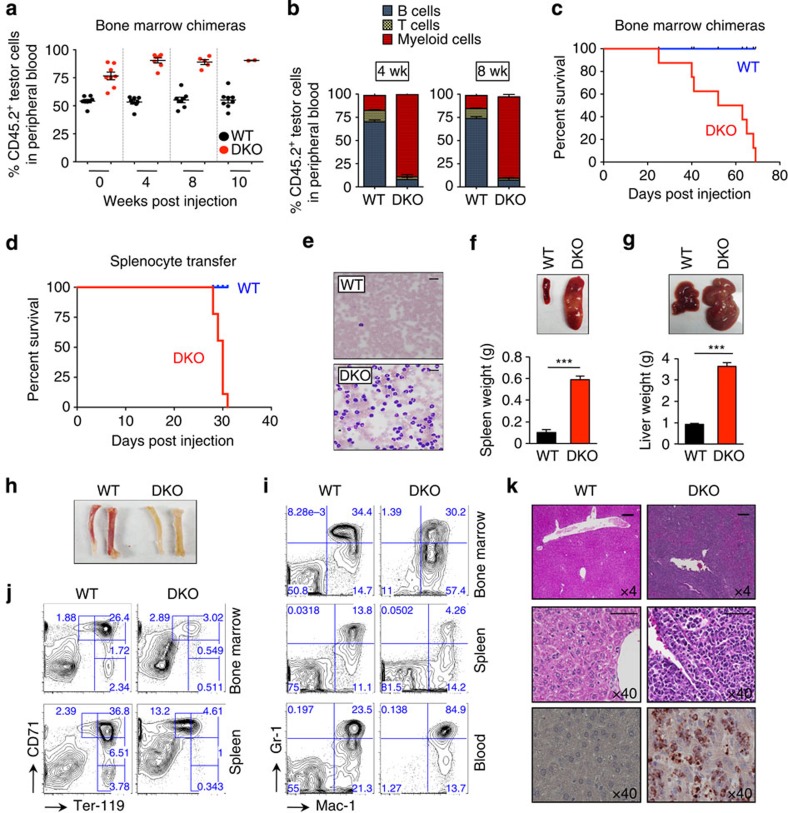
Cell-autonomous development of transplantable myeloid leukaemia in *Tet2/*3 DKO mice. (**a**) Competitive repopulation assay using bone marrow cells from inducible chimeric mice. Red blood cell-depleted CD45.2^+^ bone marrow cells from *Tet2*^+/+^ *Tet3*^*fl/fl*^ *Mx1-Cre*^−^ (WT) or *Tet2*^−/−^ *Tet3*^*fl/fl*^ *Mx1-Cre*^+^ (DKO) mice were mixed with equal numbers of CD45.1^+^ competitor cells and transplanted into lethally irradiated CD45.1^+^ congenic B6.SJL mice (see Supplementary Fig. 12a). At 4 weeks after transplantation, *Tet3* deletion was induced by administration of pIpC and peripheral blood was examined for donor chimerism at the indicated time points after pIpC injection. (**b**) Analysis of multilineage differentiation as percentage of peripheral blood cells within donor-derived (CD45.2^+^) cells. Myeloid cells (Mac-1^+^), B cells (B220^+^), T cells (CD3ɛ^+^). (**c**) Kaplan–Meier curve representing percent survival of bone marrow chimeric mice that received WT or *Tet2/3* DKO cells after pIpC injection (*n*=8 per group). No lethality was observed for recipients of WT bone marrow cells. (**d**) Kaplan–Meier curve representing percent survival of recipient mice transplanted with 2 × 10^6^ splenocytes from WT and diseased *Tet2/3* DKO mice (*n*=9 per group). (**e**) May–Grünwald–Giemsa-stained peripheral blood smears of recipient mice. Scale bar, 20 μm. (**f**,**g**) Enlargement of spleens and livers of recipient mice. Representative photographs of spleen (**f**) and liver (**g**) from recipients of control or *Tet2/3* DKO splenocytes. Weights of spleen or liver are shown below (*n*=7 per group). Means±s.e.m. are shown. ***P<0.0005 (Student's *t*-test). (**h**) Representative photographs of femurs and tibiae from recipients of WT or *Tet2/3* DKO splenocytes. (**i**) A representative flow cytometric analysis of myeloid-lineage cells (Gr-1^+^/Mac-1^+^) in bone marrow, spleen and blood of recipient mice (*n*=5 per each group). (**j**) A representative flow cytometric analysis of erythroid-lineage cells (Ter-119^+^/CD71^+^) in bone marrow and spleen of recipient mice (*n*=5 per each group). (**k**) Haematoxylin and eosin staining and immunohistochemistry (IHC) of livers with anti-myeloperoxidase show loss of normal liver structure and infiltration with myeloid cells. Top, × 4 magnification; middle and bottom, × 40 magnification. Scale bar, 200 μm (upper panel) and 60 μm (middle panel).

Bone marrow chimera and competitive engraftment experiments showed that myeloid leukaemia developed in a cell-autonomous manner in *Tet2/3* DKO mice. CD45.2^+^ bone marrow cells from WT or *Tet2*^−/−^ *Tet3*^*fl/fl*^ *Mx1-Cre*^*+*^ mice were combined with CD45.1^+^ competitor cells, followed by transplantation into lethally irradiated mice. Acute deletion of *Tet3* was induced by pIpC administration 4 weeks after transplantation (Supplementary Fig. 12a). The proportion of CD45.2^+^ donor-derived cells progressively increased for DKO donors but not for WT donors (Fig. 4a), indicating that *Tet2/3* DKO cells possessed a competitive advantage over WT control cells. Notably, recipients of WT bone marrow cells showed the normal heterogeneous profile of haematopoietic lineages, whereas the peripheral blood of chimeric mice that received *Tet2/3* DKO bone marrow cells was dominated by myeloid-lineage cells (Fig. 4b). None of the mice transplanted with DKO bone marrow survived longer than 70 days after transplantation (Fig. 4c). Moreover, the myeloid malignancy induced in *Tet2/3* DKO mice was transplantable to sublethally irradiated WT mice by transfer of splenocytes from pIpC-injected WT or *Tet2*^−/−^ *Tet3*^*fl/fl*^ *Mx1-Cre*^*+*^ mice. None of the recipient mice that received WT splenocytes developed disease. However, transfer of DKO splenocytes resulted in lethality with 100% penetrance ∼1 month after transplantation (Fig. 4d), and the disease course recapitulated that observed in the primary *Tet2/3* DKO donor mice (Fig. 4e–h, Supplementary Table 2a). Flow cytometry and histological analysis confirmed a marked expansion of myeloid cells and erythroblasts and infiltration of liver by myeloid cells (Fig. 4i–k; Supplementary Fig. 12b). Identical results were obtained when we performed the transfer experiment using splenocytes from pIpC-injected *Tet2*^*fl/fl*^ *Tet3*^*fl/fl*^ *Mx1-Cre*^*+*^ mice; bone marrow cells from tamoxifen-treated *Tet2*^*fl/fl*^ *Tet3*^*fl/fl*^ *Cre-ERT2*^*+*^ mice; or purified primary Mac-1^+^ cells from the bone marrow of pIpC-injected *Tet2*^*fl/fl*^ *Tet3*^*fl/fl*^ *Mx1-Cre*^*+*^ mice, a population that includes fully differentiated myeloid cells as well as Mac-1^+^ myeloid progenitors and cycling HSC (Supplementary Figs 13–15; Supplementary Tables 2b–d).

Together these data show that acute deletion of *Tet3* in a *Tet2*-deficient context, or concurrent deletion of both *Tet2* and *Tet3*, leads to the rapid cell-autonomous induction of an aggressive, transplantable myeloid leukaemia in mice. Limiting dilution experiments with purified stem cells will be needed to determine whether *Tet2/3 DKO* cells cause leukaemia by acquiring a true stem cell advantage such as increased self-renewal.

### Aberrant lineage priming in *Tet2/3 DKO* HSPC

**Figure 5:**
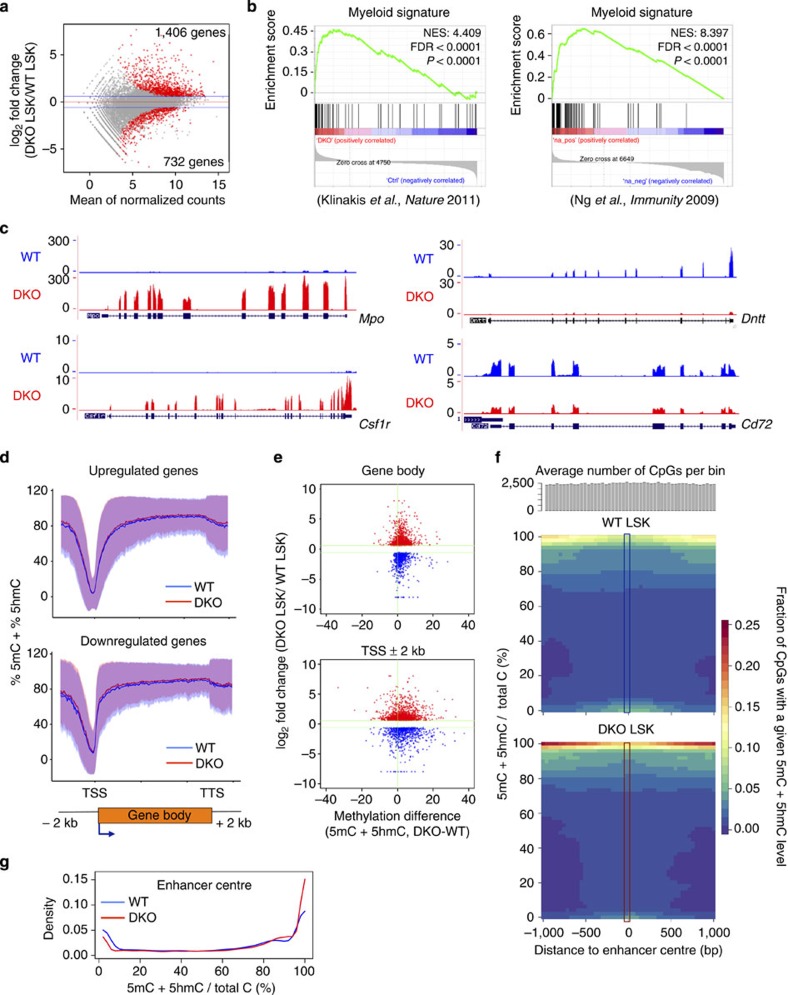
Changes in gene expression and DNA modification (5mC+5hmC) in *Tet2/3 DKO* cells. (**a**) Differential gene expression in WT versus *Tet2/3* DKO LSK cells. Red dots—differentially expressed genes (*P* value ≤0.05, fold change >1.5 or <0.67; thresholds indicated by blue lines). Grey dots—all other genes. (**b**) Gene set enrichment analysis (GSEA) of RNA-Seq data. Myeloid gene signatures are significantly enriched for genes upregulated in *Tet2/3* DKO versus WT LSK cells. (**c**) Genome browser visualization showing increased and decreased expression of myeloid (*Mpo*, *Csf1r*) and lymphoid (*Dntt*, *Cd72*) genes in *Tet2/3* DKO versus control LSK cells. (**d**) Slight but consistent increase in average DNA modification at gene regions in *Tet2/3* DKO (red lines) versus WT LSK cells (blue lines), regardless of whether gene expression is upregulated (top) or downregulated (bottom). Similar results were obtained for all genes (Supplementary Fig. 17c). Shaded areas=±1 s.d. TSS, transcription start site; TTS, transcription termination site. (**e**) Changes in gene expression versus changes in average DNA methylation for all genes differentially expressed in *Tet2/3* DKO LSK versus WT LSK cells. Top, gene bodies; bottom, promoter regions (TSS±2 kb). Each gene body/TSS region is represented by a dot. Most regions show increased methylation, regardless of whether they are up- or downregulated in DKO LSK cells relative to WT. (**f**) Active enhancer regions in LT-HSC^43^ show increased DNA modification (5mC+5hmC) in *Tet2/3 DKO* versus WT LSK cells. Each bin contains all the CpGs falling within the ∼7,000 active enhancer regions. Colour scale, density of CpGs with the indicated methylation levels. Enhancer centres in WT LSK cells show an increased fraction of unmethylated CpGs relative to *Tet2/3* DKO LSK cells (green colour at the bottom), whereas enhancer edges in *Tet2/3* DKO LSK cells show an increased fraction of methylated CpGs (5mC+5hmC) compared with WT (red colour at the top). For details, see Supplementary Methods. (**g**) Graphical representation of increased DNA modification in *Tet2/3 DKO* versus WT LSK cells. Data are from central regions of the enhancers (blue and red rectangles in **f**).

To delineate the mechanism by which Tet2 and Tet3 influence early haematopoiesis, we performed RNA sequencing on duplicate samples of WT and *Tet2/3* DKO LSK cells (Supplementary Tables 3 and 4). About twice as many genes were upregulated (*n*=1,406) as downregulated (*n*=732) in *Tet2/3* DKO compared with WT LSK cells (Fig. 5a; Supplementary Data 1). Genes upregulated in *Tet2/3* DKO compared with WT LSK cells were strongly enriched for myeloid^30^,^31^ (Fig. 5b) and pre-granulocyte/macrophage progenitor (pre-GM)^32^ gene signatures, whereas genes with HSC^33^, pre-megakaryocyte/erythrocyte progenitors^32^ and common lymphoid progenitors^32^ signatures were downregulated (Supplementary Fig. 16a). Figure 5c shows genome browser views of the RNA-seq data for selected myeloid (*Mpo, Csf1r*) or lymphoid (*Dntt, Cd72*) genes that were upregulated or downregulated, respectively, in *Tet2/3* DKO compared with control LSK cells.

Genes implicated in lymphoid, myeloid and erythroid-lineage commitment are co-expressed in HSCs, a process known as lineage priming^20^, and their expression is differentially up- or downregulated as appropriate during subsequent lineage restriction and differentiation. Compared with WT LSK cells, *Tet2/3* DKO LSK cells showed downregulation of numerous genes whose expression is enriched in HSC^31^,^34^,^35^, including ‘stem' genes^31^ and genes reported to induce reprogramming of committed haematopoietic cells towards induced HSCs^34^. They also showed downregulation of *s-mpp* genes^31^ expressed in multipotent progenitors (MPP) and LMPPs; erythroid genes that are primed in HSC (*s-ery*)^31^; and lymphoid genes such as *Dntt*, *Satb1*, *Notch1*, *Sox2*, *Cd72* and *Ets1* (Supplementary Fig. 16b–d). In contrast, they tended to upregulate myeloid-lineage genes that were primed in HSC (*s-myly*)^31^, and displayed premature induction of many myeloid genes normally expressed in GMP (*d-my*)^31^ (Supplementary Fig. 16d).

Taken together, these results indicate that Tet2 and Tet3 normally induce stem cell-related genes, promote lymphoid and erythroid priming, and prevent the premature induction of late myeloid genes in early haematopoietic progenitors, thus supporting the undifferentiated state of HSCs; consequently, their loss leads to myeloid skewing. These molecular findings are well aligned with the observed developmental phenotype of the *Tet2/3* DKO bone marrow, in which production of LMPPs and megakaryocyte-erythroid progenitors is impaired, but production of myeloid progenitors (particularly GMPs) remains intact.

### Lack of relation between transcription and DNA methylation

To relate changes in gene expression to changes in DNA modification, we performed whole-genome bisulfite sequencing on WT and *Tet2/3* DKO LSK cells (Supplementary Table 5; see Methods). Because bisulfite sequencing does not distinguish between 5mC and 5hmC^36^, we use the term DNA modification in preference to DNA methylation; however, because of the very low level of 5hmC in Tet2/3-deficient bone marrow cells (Fig. 1a), most of the observed signal of unconverted C is likely due to 5mC with little or no contribution from 5hmC.

Overlaid upon the typical bimodal distribution of DNA methylation in both WT and *Tet2/3* DKO LSK cells, we observed a slight but consistent increase of 5mC+5hmC throughout the transcription start site (TSS)/promoter and gene body regions in *Tet2/3* DKO compared with WT LSK cells (Supplementary Fig. 17a–c). This increase was reproducible (compare the triplicate biological samples from WT and DKO cells; Supplementary Fig. 17c, left panel); specific (it was not seen in randomly chosen genome fragments of similar size; Supplementary Fig. 17c, right panel); and limited in extent (Supplementary Fig. 17a,b), most likely reflecting the fact that 5hmC is only a minor proportion of 5mC (5–10%). The results support the well-established notion that TET proteins regulate DNA cytosine modification and facilitate DNA demethylation^1^,^5^,^7^,^37^,^38^. A similar small average increase was observed in differentially expressed genes, regardless of whether they were upregulated or downregulated in *Tet2/3* DKO compared with WT cells (Fig. 5d).

To examine changes in DNA modification at the level of individual TSS regions and gene bodies, we divided the distance from the TSS to the transcription termination site for each differentially expressed gene into 100 bins, calculated the average change in DNA modification within each gene body (bins 26–100) and at each promoter (±2 kb relative to the TSS) and plotted the values against the log2-fold change in expression of the corresponding gene (Fig. 5e). The resulting dot plot confirms that the slight to moderate increase in average DNA modification at gene bodies and promoters in *Tet2/3* DKO compared with WT LSK cells occurs independently of the direction of change in gene transcription, since it is similar for upregulated versus downregulated genes.

Since these findings run counter to previous reports of a strong inverse correlation between promoter methylation and gene expression^39^, we also examined differentially modified regions, CpG islands (CGIs)^40^, CGI shores^41^ and the subset of long (⩾3.5 kb) undermethylated regions classified as ‘canyons'^42^. The majority of differentially modified regions (3,864/5,555; ∼70%) gained methylation in *Tet2/3* DKO LSK cells compared with WT; they were depleted from intergenic regions and enriched in promoter/TSS regions and gene bodies (Supplementary Fig. 17d; Supplementary Table 6). As previously described^41^, DNA methylation increased primarily at CGI shores rather than within CGIs in *Tet2/3* DKO compared with WT cells, but only a minority of CGI shores within or at promoter regions (±2 kb) showed increased DNA modification (1,539/12,005 (12.8%); Supplementary Fig. 18a, left). Moreover, for CGI shores within or at promoter regions (±2 kb), there was no clear correlation between the observed change in DNA modification and the change in expression level of the associated genes (Supplementary Fig. 18a, right; Supplementary Data 2).

Among the undermethylated regions in WT LSK cells, we defined 1,135 canyons^42^ (see Methods); of these, 846 were located in close proximity (<2 kb) to 841 gene promoters (Supplementary Fig. 18b, top). Only 105 of these genes (12.4%) showed significantly altered expression, but more were downregulated (*n*=77) than upregulated (*n*=28) in *Tet2/3* DKO compared with WT LSK cells (Supplementary Fig. 18b, top). As expected, more canyons (465; 41%) gained DNA methylation at canyon edges in *Tet2/3* DKO LSK cells relative to WT, compared with 59 (5.25%) that lost DNA methylation (Supplementary Fig. 18b, middle and bottom; Supplementary Fig. 18c); but again, only a minority of genes associated with shrinking and expanding canyons showed altered expression by RNA-seq (93/524; 17.7%). Of the 86 differentially expressed genes associated with shrinking canyons, 67 were downregulated and 19 were upregulated (Supplementary Fig. 18d, left; Supplementary Data 3); this difference was statistically significant (*P* value<2.834 × e−96, *χ*^2^-test).

Finally, *Tet2/3 DKO* LSK cells showed moderate hypermethylation compared with WT LSK cells at active enhancers previously defined in LT-HSC^43^ (Fig. 5f,g); note the decrease in modification from WT to DKO in the area of low modification at the enhancer centre, and the increase in density of CpGs with a high modification level across the entire enhancer region in the red area of near 100% modification from DKO to WT (Fig. 5f). Thus some active enhancers have low levels of DNA modification (5mC+5hmC) and TET proteins maintain this unmodified state; however, some active enhancers contain high levels of DNA modification, potentially 5hmC^44^, but these regions are also subject to increased modification (most likely hypermethylation) upon loss of TET proteins. We have not attempted to correlate changes in DNA modification at enhancers to gene expression, since it is not yet straightforward to identify the gene(s) controlled by a given enhancer^43^,^45^.

Overall, TET loss-of-function is primarily associated with canyon shrinking and enhancer hypermethylation, and canyon shrinking is more strongly associated with downregulation than upregulation of gene expression. The results are consistent with previous findings that increased DNA methylation at canyon edges often results in decreased gene expression^42^, and confirm, for some genes, that increased promoter methylation correlates with decreased gene expression^39^. However, the identities of the up- and downregulated genes did not generally shed light on myeloid skewing or downregulation of HSC-expressed, erythroid and lymphoid genes in *Tet2/3* DKO compared with WT LSK cells. For instance, *Car2* (encoding carbonic anhydrase 2) and *Gcnt2* (encoding glucosaminyl (*N*-acetyl) transferase 2, the I-branching enzyme that generates the I blood group antigen) were among the most downregulated genes associated with shrinking canyons in *Tet2/3* DKO LSK cells compared with WT (Supplementary Fig. 18d,e); to the best of our knowledge, however, the functions of these genes have not been associated specifically with haematopoietic differentiation or oncogenesis.

### *Tet2/3 DKO* cells show DNA damage and impaired DNA repair

**Figure 6:**
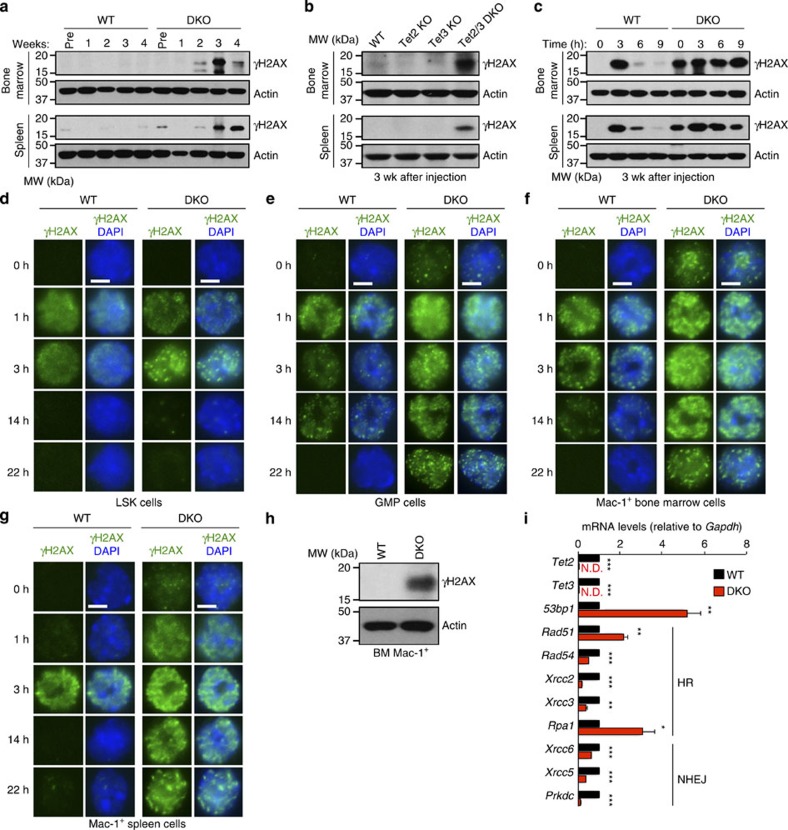
Loss of Tet2 and Tet3 results in DNA damage and impaired DNA repair. (**a**–**c**) γH2AX levels in whole-cell lysates from bone marrow (top) and spleen (bottom), assessed by immunoblotting. Actin serves as a loading control. (**a**) Progressive increase in γH2AX levels in bone marrow and spleen of *Tet2/3* DKO mice with time after pIpC injection. (**b**) Accumulation of γH2AX in bone marrow and spleen of *Tet2/3 DKO* but not *Tet2KO* or *Tet3KO* mice, 3 weeks after pIpC injection. (**c**) Impaired DNA repair in *Tet2/3 DKO* cells. WT and DKO mice were exposed to 6 Gy of ionizing radiation 3 weeks after pIpC injection, single-cell suspensions of bone marrow and spleens were prepared at the indicated times, and DNA repair kinetics were assessed by immunoblotting. Similar results were obtained 1 week after pIpC injection (Supplementary Fig. 19a). (**d**–**g**) DNA damage repair is impaired in *Tet2/3 DKO* myeloid-lineage cells. LSK (**d**), GMP (**e**) and Mac-1^+^ cells in bone marrow (**f**), or Mac-1^+^ cells in spleen (**g**) were sorted from WT and DKO mice at 3 weeks after pIpC injection, exposed to 6 Gy of ionizing radiation, and DNA repair kinetics were assessed by immunocytochemistry. LSK cells repair DNA damage efficiently, whereas GMP and Mac-1^+^ cells do not. Similar results were obtained using tamoxifen-injected *Tet2*^*fl/fl*^ *Tet3*^*fl/fl*^ *ERT2-Cre*^*+*^ mice (Supplementary Fig. 19b–d). Scale bar, 5 μm. (**h**) Increase in γH2AX levels in Mac-1^+^ cells sorted from bone marrow of DKO mice 3–4 weeks after pIpC injection. (**i**) TET proteins control expression of DNA repair genes in myeloid cells. Quantitative reverse transcription–PCR of Mac-1^+^ cells sorted from bone marrow of WT (*Tet2*^*fl/fl*^ *Tet3*^*fl/fl*^) and DKO (*Tet2*^*fl/fl*^ *Tet3*^*fl/fl*^ *Mx1-Cre*^*+*^) mice 3–4 weeks after pIpC injection. Results are expressed as fold change over WT cells (set to 1; three independent experiments; means±s.e.m.). Similar results were observed in sorted Mac-1^+^ cells from WT (*Tet2*^*+/+*^*Tet3*^*fl/fl*^) and *Tet2*^−/−^ *Tet3*^*fl/fl*^ *Mx1-Cre*^*+*^ mice (Supplementary Fig. 19e). HR, homologous recombination; NHEJ, non-homologous end-joining; ND, not detected. *P<0.05, **P<0.005, ***P<0.0005 (Student's *t*-test).

Genome instability, arising from replication stress or DNA lesions coupled with defective DNA damage checkpoints and repair, promotes the accumulation of mutations and chromosomal aberrations that drive progression to malignancy^46^. Beginning at 2 weeks after pIpC injection, there was strong spontaneous accumulation of phosphorylated histone H2A.X (γH2AX), an early marker of DNA damage^46^, in cells from the bone marrow and spleen of *Tet2/3* DKO but not WT, Tet2KO or Tet3KO mice (Fig. 6a,b). When we exposed WT and *Tet2/3* DKO mice to 6 Gy of ionizing irradiation at 1 and 3 weeks after pIpC injection, there was strong γH2AX induction at 3 h after irradiation, but the DNA double-strand breaks were rapidly resolved (6–9 h) in WT cells as shown by the disappearance of γH2AX. In cells from injected DKO mice, however, γH2AX persisted at high levels until 9 h after irradiation (Fig. 6c; Supplementary Fig. 19a), indicating that γH2AX accumulation in the DKO cells was due to impairment of DNA damage repair.

Notably, this defect was most prominent in differentiated cell populations compared with early precursors. LSK, GMP and Mac-1^+^ cells were isolated from bone marrow and spleen of WT and DKO mice that had been injected with pIpC 3 weeks previously, and exposed to ionizing radiation; the appearance and resolution of DNA double-strand breaks was then examined by immunocytochemistry for γH2AX (Fig. 6d–g). Early precursor (LSK) cells from WT and *Tet2/3* DKO bone marrow developed equivalent levels of γH2AX foci at 1 and 3 hr after irradiation, and these were efficiently resolved with similar kinetics (Fig. 6d); further along the differentiation pathway, GMP cells from DKO mice displayed heightened levels of ionizing radiation-induced γH2AX foci compared with WT GMP cells and were less capable of removing γH2AX over time (Fig. 6e); and finally, the most mature cell population, Mac-1^+^ myeloid cells sorted from bone marrow and spleen, displayed high levels of γH2AX foci even before irradiation (0 h), and were unable to repair γH2AX foci even 22 h after irradiation (Fig. 6f,g). Similar results were obtained in *Tet2*^−/−^ *Tet3*^fl/fl^ *ERT2-Cre*^+^ mice when *Tet3* was deleted by tamoxifen treatment *in vivo* (Supplementary Fig. 19b–d).

We showed that TET proteins influence the expression of DNA damage repair genes in myeloid cells, and that TET loss-of-function can in some cases lead to overt genomic instability. Mac-1^+^ cells were sorted from WT (*Tet2*^*fl/fl*^ *Tet3*^*fl/fl*^*or* *Tet2*^*+/+*^*Tet3*^*fl/fl*^) and DKO (*Tet2*^*fl/fl*^ *Tet3*^*fl/fl*^ *Mx1-Cre*^*+*^) mice at 3∼4 weeks after pIpC injection (Fig. 6h), and quantitative reverse transcription–PCR was performed to assess the expression of genes implicated in homologous recombination and non-homologous end-joining. The expression of *53bp1*, *Rad51* and *Rpa1* was increased in *Tet2/3* DKO cells compared with WT cells, whereas the expression of *Rad54*, *Xrcc2*, *Xrcc3*, *Xrcc5*, *Xrcc6* and *Prkdc* was decreased (Fig. 6i). Similar results were obtained using *Tet2*^−/−^ *Tet3*^*fl/fl*^ *Mx1-Cre*^*+*^mice (Supplementary Fig. 19e). We then submitted bone marrow samples from four sick *Tet2/3*-deficient DKO mice for G-banded karyotyping; these samples contain primarily differentiated Mac-1^+^ cells. In one of these samples, 6 of 20 DKO cells examined displayed an unbalanced translocation of distal chromosome 6 to distal chromosome 3 (Supplementary Fig. 20). Together, these results link TET loss-of-function to progressive impairment of the DNA damage response, and show that TET function is important for genome integrity in certain cell types.

## Discussion

In this study, we demonstrate for the first time a causal association of TET loss-of-function with the rapid occurrence of a malignant cancer. Induced deficiency of both Tet2 and Tet3 in mice leads to the almost complete loss of 5hmC in the bone marrow and spleen, and concomitantly to the rapid and cell-autonomous development of an aggressive, fully penetrant and transmissible myeloid leukaemia. This phenotype is not observed with mice lacking only Tet2 or Tet3, indicating that Tet2 and Tet3 function redundantly to suppress oncogenesis.

Typically, cancer initiation, progression and metastasis involve multiple steps of progressive cellular dysfunction, including loss of genome integrity and the consequent acquisition of mutations that promote cell transformation^47^. These processes are rarely the result of a single oncogenic hit, but require multiple mutagenic events that generally accumulate over a long time period. The striking feature of our study is that acute TET loss-of-function accelerates oncogenesis, such that uncontrolled cell proliferation and genome instability develop in a compressed time span of only ∼4–5 weeks. Profound loss of function is required, since mice lacking individual TET proteins may show precancerous symptoms but develop full-blown malignancy only sporadically and at a very late age, if at all^8^,^15^,^16^,^17^,^18^. Thus TET proteins are required to maintain genome stability, and collectively constitute a major class of tumour suppressors in cells.

While *TET2* mutations are thought to be driver mutations in haematopoietic malignancies, whole-exome sequencing of large cancer cohorts has revealed only rare mutations in *TET1* or *TET3* (refs 19, 48, 49). However, numerous studies have demonstrated substantial decreases in 5hmC levels in diverse human cancers, even in the absence of *TET*-coding region mutations^2^. In several cases, decreased 5hmC levels reflect decreased TET messenger RNA or protein expression secondary to upregulation of E3 ligases or microRNAs^50^,^51^,^52^,^53^, or correlate with increased DNA methylation at *TET* gene promoters^18^,^54^. In other cases, inhibition of TET function appears to be the underlying cause: for instance in many glioblastomas and myeloid leukaemias, recurrent dominant mutations in *IDH1* and *IDH2*, the cellular enzymes that produce 2-oxoglutarate, result in massively increased levels of 2-hydroxyglutarate^55^, an ‘oncometabolite' that interferes with the activity of TET enzymes and other dioxygenases^56^. On the basis of these findings, we explored the relation of profound TET loss-of-function to cell lineage specification and oncogenic transformation. Our data document a causal relation between TET loss-of-function and cancer, and argue against the possibility that decreased 5hmC in cancer cells merely reflects their rapid proliferation.

Notably, we observed no consistent relation between DNA modification and gene expression in DKO versus WT LSK cells. As expected from the fact that TET proteins facilitate DNA demethylation, early progenitor (LSK) cells from *Tet2/3 DKO* mice showed increased DNA modification relative to WT LSK cells, and the biggest differences were observed at genomic regions where DNA modification levels are intermediate: canyon edges, gene bodies and enhancers. Moreover, transcriptional profiling revealed clear changes in gene expression in DKO versus WT LSK cells, including decreased expression of stem cell genes, altered lineage priming and strong myeloid skewing. However, only a minority of regions with increased DNA methylation displayed decreased transcription of the associated genes. Potentially, increased DNA methylation might better correlate with decreased gene transcription in GMP or Mac-1^+^ cells that are further along the myeloid differentiation pathway than the LSK progenitor cells examined here. If so, it would underscore the view that altered DNA methylation is a late consequence of altered gene transcription rather than its direct cause (reviewed in refs 57, 58).

What mechanisms underlie the lineage skewing induced by deletion of both Tet2 and Tet3, if increased DNA methylation is not the primary cause? An important factor may be the profound loss of TET-generated oxi-mC bases in *Tet2/3 DKO* cells. 5hmC is present at high levels at active enhancers and the gene bodies of highly transcribed genes^59^; moreover, both 5hmC and 5fC are stable modifications that are lost through DNA replication in somatic cells^60^. Therefore, profound loss of 5hmC in *Tet2/3 DKO* cells would be expected to affect gene transcription only after several replication cycles. If, as recently reported^61^, 5fC and 5caC directly affect the processivity of RNA polymerase II, oxi-mC would be most heavily diluted, and gene transcription would be most prominently decreased, at genes expressed in actively proliferating cells that encode key signalling proteins and transcriptional regulators that shape cell lineage commitment and function^62^.

The hypothesis that oxi-mC may be more relevant than 5mC is also supported by recent findings that mice lacking either Dnmt3a or Tet2 in HSC show similar phenotypes in terms of augmented HSC repopulation capacity, myeloid skewing and ultimately myeloid transformation^8^,^15^,^16^,^17^,^63^,^64^,^65^. DNMT3A produces 5mC, the substrate for TET proteins^66^. Therefore, impaired function of these two enzymes would be expected to have opposite effects on DNA methylation, resulting in overall decreases and increases in 5mC, respectively, but would be similar in terms of decreasing oxi-mC (Dnmt3a-deficient cells have lower levels of the TET substrate 5mC and therefore less oxi-mC, whereas *TET2* mutations are loss-of-function mutations and therefore also have low oxi-mC^2^). Thus the similar biological phenotypes of Tet2- and Dnmt3a-deficient mice could plausibly reflect loss of oxi-mC in each case.

In addition to skewing to the myeloid lineage, *Tet2/3 DKO* cells display uncontrolled proliferation and rapid progression to oncogenic transformation, a process accompanied by increased levels of the DNA damage marker phospho-H2AX and slower resolution of this mark. These features were most apparent in cells that had progressed along the differentiation pathway rather than in early precursors. Mac-1^+^ cells sorted from bone marrow and spleen of *Tet2/3 DKO* mice showed altered expression of many genes encoding proteins in the homologous recombination and non-homologous end-joining pathways of DNA repair, but it is not clear whether this is a cause or a consequence of the observed defects in DNA damage responses. Further mechanistic studies are necessary to determine how TET loss-of-function leads to impaired DNA damage.

## Methods

### Acute deletion of *Tet2* and *Tet3*

*Tet2*^−/−^, *Tet2*^*fl/fl*^ and *Tet3*^*fl/fl*^ mice have been described previously^12^,^17^. To induce conditional excision of floxed alleles using the *Mx1-Cre* recombinase, pIpC (polyinosinic–polycytidylic acid, Sigma: 300 μg per mouse) in PBS was injected five times intraperitoneally every other day for a total of five injections. To delete floxed alleles using the *ERT2-Cre* recombinase, tamoxifen (Sigma) was solubilized at 10 mg ml^−1^ in corn oil (Sigma) and delivered into mice by intraperitoneal injection of 2 mg tamoxifen per mouse every day for 5 consecutive days. The day of the last pIpC or tamoxifen injection was designated Day 0 (Fig. 1b). In Fig. 1b, WT indicates *Mx1-Cre*^+^; *Tet2*^+/+^ *Tet3*^*fl/fl*^ *Mx1-Cre*^−^ or *Tet2*^+/+^ *Tet3*^*fl/+*^ *Mx1-Cre*^−^ genotypes; DKO indicates *Tet2*^−/−^ *Tet3*^*fl/fl*^ *Mx1-Cre*^+^ genotype; *Tet2*-deficient (T2KO) indicates *Tet2*^−/−^ *Tet3*^*fl/fl*^ *Mx1-Cre*^−^ or *Tet2*^−/−^ *Mx1-Cre*^+^ genotypes; and *Tet3*-deficient (Tet3KO) indicates the *Tet3*^*fl/fl*^ *Mx1-Cre*^+^ genotype. In Fig. 3c, WT indicates *Tet3*^*fl/fl*^*, Tet2*^*fl/fl*^ *Tet3*^*fl/+*^ *or Tet2*^*fl/fl*^ *Tet3*^*fl/fl*^; T2KO indicates *Tet2*^−/−^ or *Tet2*^−/−^ *Tet3*^*fl/fl*^; T3KO indicates *Tet3*^*fl/fl*^ *Mx1-Cre*^*+*^; and DKO indicates *Tet2*^−/−^ *Tet3*^*fl/fl*^ *Mx1-Cre*^*+*^ mice. For all other experiments, *Tet2*^*+/+*^ *Tet3*^*fl/fl*^ *Mx1-Cre*^−^ or *Tet2*^*+/+*^ *Tet3*^*fl/+*^ *Mx1-Cre*^−^ mice were used as WT controls. Efficient gene deletion in sorted HSPCs was verified by real-time reverse transcription–PCR. The pIpC-injected mice were assessed on a daily basis for survival and development of any haematopoietic abnormalities. For all experiments described here, mice were killed immediately if they were hunched or slow-moving, had piloerection of the fur, if their eyes were sunken or closed or they were not responsive to handling. To draw a Kaplan–Meier survival plot, it was assumed that the mice would live 1 more day unless killed, so death was not required as an end point. All cell or mouse irradiation procedures were performed using RS2000 Biological Irradiator (Rad Source Technologies, Inc.). All animal procedures were approved by the La Jolla Institute Institutional Animal Care and Use Committee and were conducted in accordance with institutional guidelines.

### *In vivo* transplantation experiments

For competitive engraftment assays, total bone marrow cells were isolated from tibiae and femurs of *Tet2*^*+/+*^ *Tet3*^*fl/fl*^ *Mx1-Cre*^−^ or *Tet2*^−/−^ *Tet3*^*fl/fl*^ *Mx1-Cre*^+^ testor mice (CD45.2^+^ background), and red blood cells were lysed by incubation with ACK lysis buffer (0.15 M NH_4_Cl, 10 mM KHCO_3_ and 0.1 mM EDTA) at room temperature for 1 min. Cells were counted using BD Accuri C6 Flow Cytometer (BD Biosciences). One million tester-derived cells were mixed with equal number of competitor bone marrow cells (CD45.1^+^ background) and the mixtures were injected into lethally irradiated (10 Gy, two split doses with a 3-h interval) congenic (CD45.1^+^) B6.SJL-Ptprc^a^Pepc^b^/BoyJ mice (Jackson Laboratories). At 4 weeks post transplant, chimeric mice were administered intraperitoneally with 300 μg of poly(I:C) per mouse in PBS every other day for a total of five injections (week 0) to excise floxed alleles. To evaluate the percentage of tester-derived cells and different lineage contribution, peripheral blood was collected from the chimeric mice via retro-orbital sinus under anaesthesia at 4, 8 and 10 weeks after injection, depleted of red blood cells and stained with combinations of antibodies against CD45.1, CD45.2, CD3ɛ, CD19 and Mac-1, and the percentage of haematopoietic subsets within the CD45.2^+^ population was analysed by flow cytometry. Kaplan–Meier plots were drawn based on the monitoring criteria described above. To evaluate the transplantability of disease, splenocytes were isolated from *Tet2*^*+/+*^ *Tet3*^*fl/fl*^ *Mx1-Cre*^−^ and *Tet2*^−/−^ *Tet3*^*fl/fl*^ *Mx1-Cre*^+^ (for Fig. 4d–k) or *Tet2*^*fl/fl*^ *Tet3*^*fl/fl*^ *Mx1-Cre*^−^ and *Tet2*^*fl/fl*^ *Tet3*^*fl/fl*^ *Mx1-Cre*^+^ (for Supplementary Fig. 13) mice at about 3.5 or 4.5 weeks following pIpC injection, respectively, depleted of red blood cells and 2 × 10^6^ cells in PBS were injected intravenously via retro-orbital route into sublethally irradiated (600 cGy) C57BL/6 recipient mice. For transfer of bone marrow cells in Supplementary Fig. 14, bone marrow cells were isolated from *Tet2*^*fl/fl*^ *Tet3*^*fl/fl*^ *ERT2-Cre*^−^ and *Tet2*^*fl/fl*^ *Tet3*^*fl/fl*^ *ERT2-Cre*^+^ at 3 weeks following tamoxifen injection, depleted of red blood cells and 2 × 10^6^ cells in PBS were injected intravenously via retro-orbital route into sublethally irradiated (600 cGy) C57BL/6 recipient mice. For transfer of Mac-1^+^ cells in Supplementary Fig. 15, 2 × 10^6^ Mac-1^+^ cells sorted from *Tet2*^*fl/fl*^ *Tet3*^*fl/fl*^ *Mx1-Cre*^−^ and *Tet2*^*fl/fl*^ *Tet3*^*fl/fl*^ *Mx1-Cre*^+^ at about 4.5 weeks following pIpC injection were combined with equal number of WT bone marrow helper cells, followed by injection into lethally irradiated (10 Gy, two split doses with a 3-h interval) C57BL/6 recipient mice. Then, the recipient mice were monitored for survival every day. Mice were killed when they met our monitoring criteria described above and histological or cytological analyses were carried out as described.

### Colony-forming assay *in vitro*

For colony-forming assay using splenocytes and liver cells (Fig. 1g), cells were harvested from spleens and livers of *Tet2*^*+/+*^ *Tet3*^*fl/fl*^ *Mx1-Cre*^−^ and *Tet2*^−/−^ *Tet3*^*fl/fl*^ *Mx1-Cre*^+^ mice about 3 weeks following pIpC injections, depleted of red blood cells and plated at 10^5^ cells per 35-mm dish in duplicate into cytokine-supplemented methylcellulose medium (Methocult M3534, STEMCELL Technologies). Colony-forming units were photographed and counted on day 7.

For colony-forming assay using sorted LSK or GMP cells (Fig. 3c), LSK or GMP cells were sorted from the bone marrow of WT (*Tet2*^*fl/fl*^, *Tet2*^*fl/fl*^ *Tet3*^*fl/fl*^ or *Tet3*^*fl/+*^ *Tet3*^*fl/fl*^), Tet2KO (*Tet2*^−/−^ or *Tet2*^−/−^ *Tet3*^*fl/fl*^), Tet3KO (*Tet3*^*fl/fl*^ *Mx1-Cre*^*+*^) or DKO (*Tet2*^−/−^ *Tet3*^*fl/fl*^ *Mx1-Cre*^*+*^) mice at 4 weeks after pIpC injection and seeded at a density of 1,000 cells per 35-mm dish in duplicate into cytokine-supplemented methylcellulose media (Methocult M3534, STEMCELL Technologies). For experiments in Supplementary Fig. 9c–f, LSK cells were sorted from the bone marrow of *Tet2*^*+/+*^ *Tet3*^*fl/fl*^ *ERT2-Cre*^−^ and *Tet2*^−/−^ *Tet3*^*fl/fl*^ *ERT2-Cre*^+^ mice and seeded at a density of 1,000 cells per 35-mm dish in duplicate into cytokine-supplemented methylcellulose media (Methocult M3534). To induce *Tet3* deletion *in vitro*, 4-hydroxytamoxifen (Sigma) was dissolved in ethanol at 4 mg ml^−1^ and diluted immediately before treatment for a final concentration of 1 μM. Colonies propagated in culture were scored after 7–9 days. For serial replating experiments, colonies in the dish were pooled and cells were resuspended and counted, and re-seeded at a density of 1,000 cells per dish in duplicate for a total of four platings.

## Additional information

Accession numbers: The RNA sequencing and whole-genome bisulfite sequencing data have been deposited in the GEO database under accession code GSE72630.

**How to cite this article:** An, J. *et al*. Acute loss of *TET* function results in aggressive myeloid cancer in mice. *Nat. Commun.* 6:10071 doi: 10.1038/ncomms10071 (2015).

## Supplementary Material

Supplementary Information Supplementary Figures 1-21, Supplementary Tables 1-6, Supplementary Methods and Supplementary References

Supplementary Data 1 List of differentially expressed genes.

Supplementary Data 2 Differentially expressed genes with CGI shores more methylated in DKO LSK

Supplementary Data 3 Genes associated with shrinking canyons.
