# Supplementary material for: Acute loss of TET function results in aggressive myeloid cancer in mice
Source: Nat Commun. 2015 Nov 26;6:10071. doi: 10.1038/ncomms10071 (PMC4674670; doi:10.1038/ncomms10071)
Supplement: Supplementary Information — Supplementary Figures 1-21, Supplementary Tables 1-6, Supplementary Methods and Supplementary References [file ncomms10071-s1.pdf]

Supplementary Fig. 1

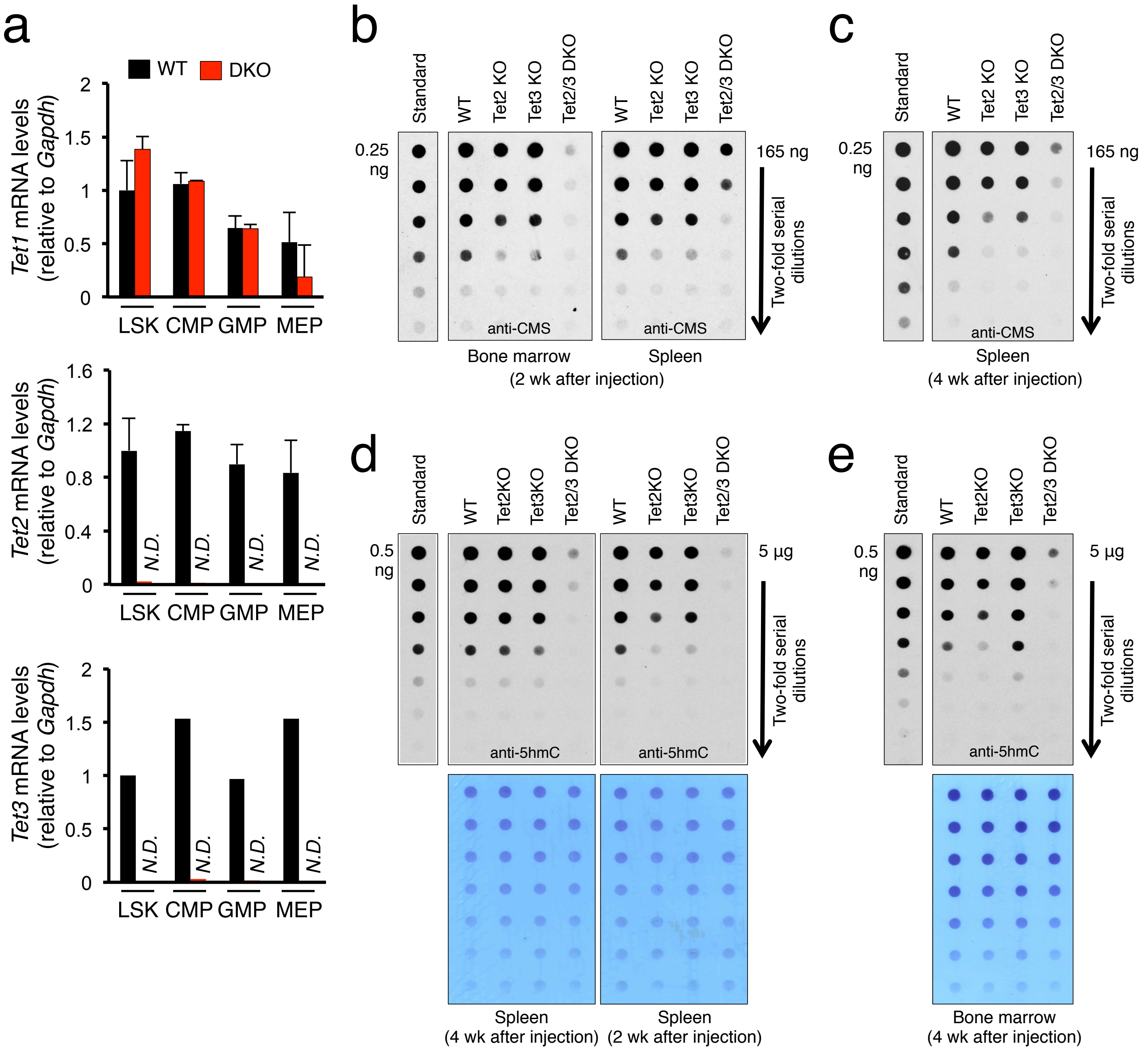

Supplementary Figure 1. Loss of genomic 5hmC in hematopoietic cells of *Tet2/3* DKO mice.
(a) Expression of *Tet1*, *Tet2* and *Tet3* in sorted LSK and myeloid progenitor cells. Cells were isolated from bone marrow of WT ($Tet3^{fl/fl}$) or DKO ($Tet2^{-/-}$ $Tet3^{fl/fl}$ $Mx1\text{-}Cre^{+}$) mice at 2 weeks after pIpC administration by flow cytometry, and quantitative RT-PCR was performed. The relative levels of mRNAs after normalization to the level of *Gapdh* mRNA in the same cell population are shown, with the amount in the WT LSK cells arbitrarily set to 1. *N.D.*, not detected.
(b-e) Quantification of 5hmC levels in cells from *Tet2/3* DKO mice. (**b, c**) At two (**b**) or four (**c**) weeks following pIpC administration, 5hmC levels in the bone marrow or spleen were quantified by dot blot assay with anti-CMS antibody after treatment of genomic DNA with bisulfite. A synthetic oligonucleotide with a known amount of CMS was used as standard.
(**d, e**) *Top panels*, At two or four weeks following pIpC administration, 5hmC levels in the spleen (**d**) or bone marrow (**e**) were quantified by dot blot assay with anti-5hmC antibody (*top panels*). *Bottom panels*, Methylene blue staining was used to monitor equivalent DNA loading. A synthetic oligonucleotide with a known amount of 5hmC was used as standard.

Supplementary Fig. 2

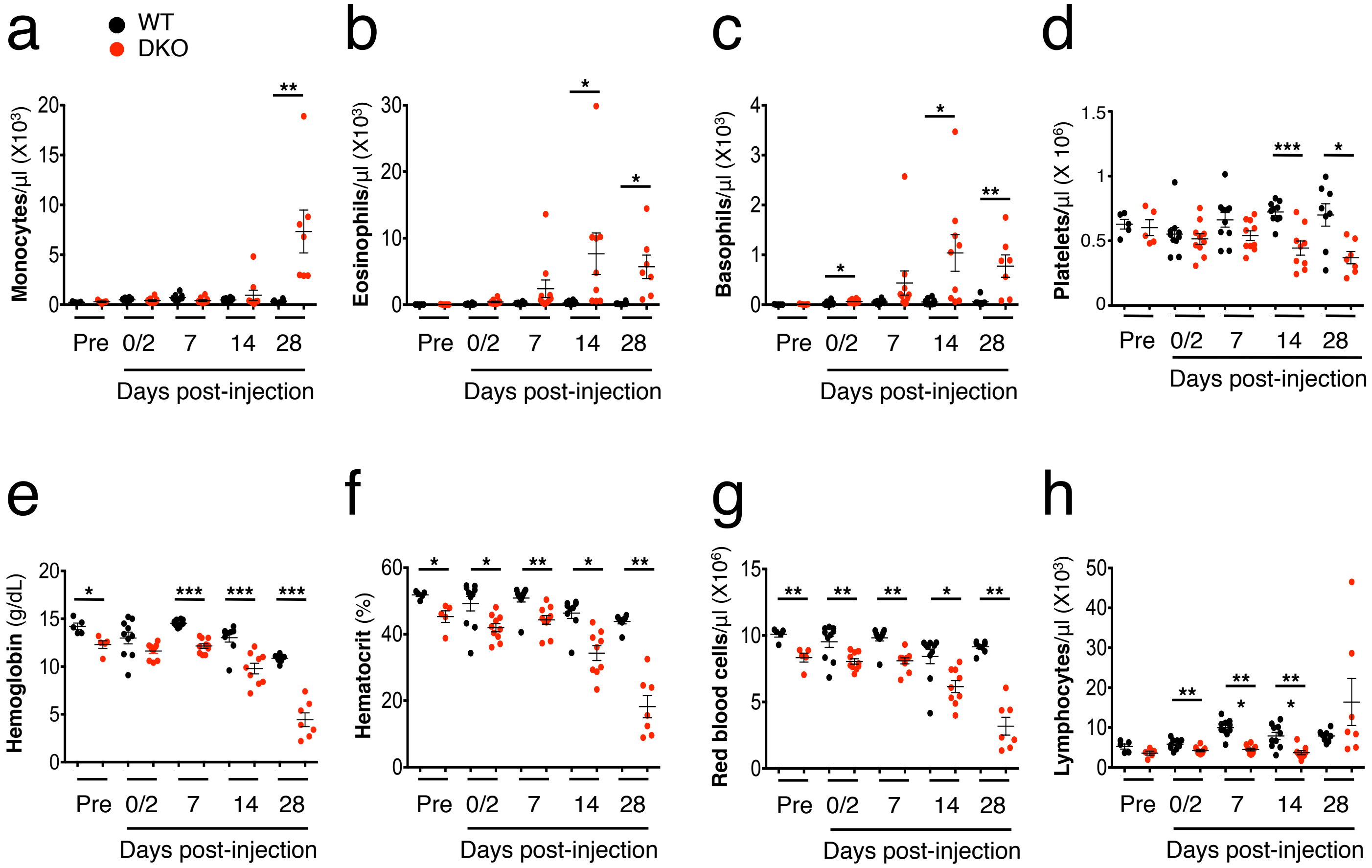

Supplementary Figure 2. Hematopoietic cell numbers in peripheral blood of WT and *Tet2/3* DKO mice.
(a-g) Time-course analysis of peripheral blood cell counts. *Tet2/3* DKO mice developed progressive monocytosis (**a**), eosinophilia (**b**), basophilia (**c**), thrombocytopenia (**d**) and anemia (**e-g**).
(h) There was a slight decrease in lymphocyte numbers in the peripheral blood of DKO mice after pIpC injection although the difference at day 28 was not statistically significant (n = 7~10 per each genotype at each time point examined). Means ± SEM are shown. *$P < 0.05$, **$P < 0.005$, ***$P < 0.0005$ (Student's t test).

Supplementary Fig. 3

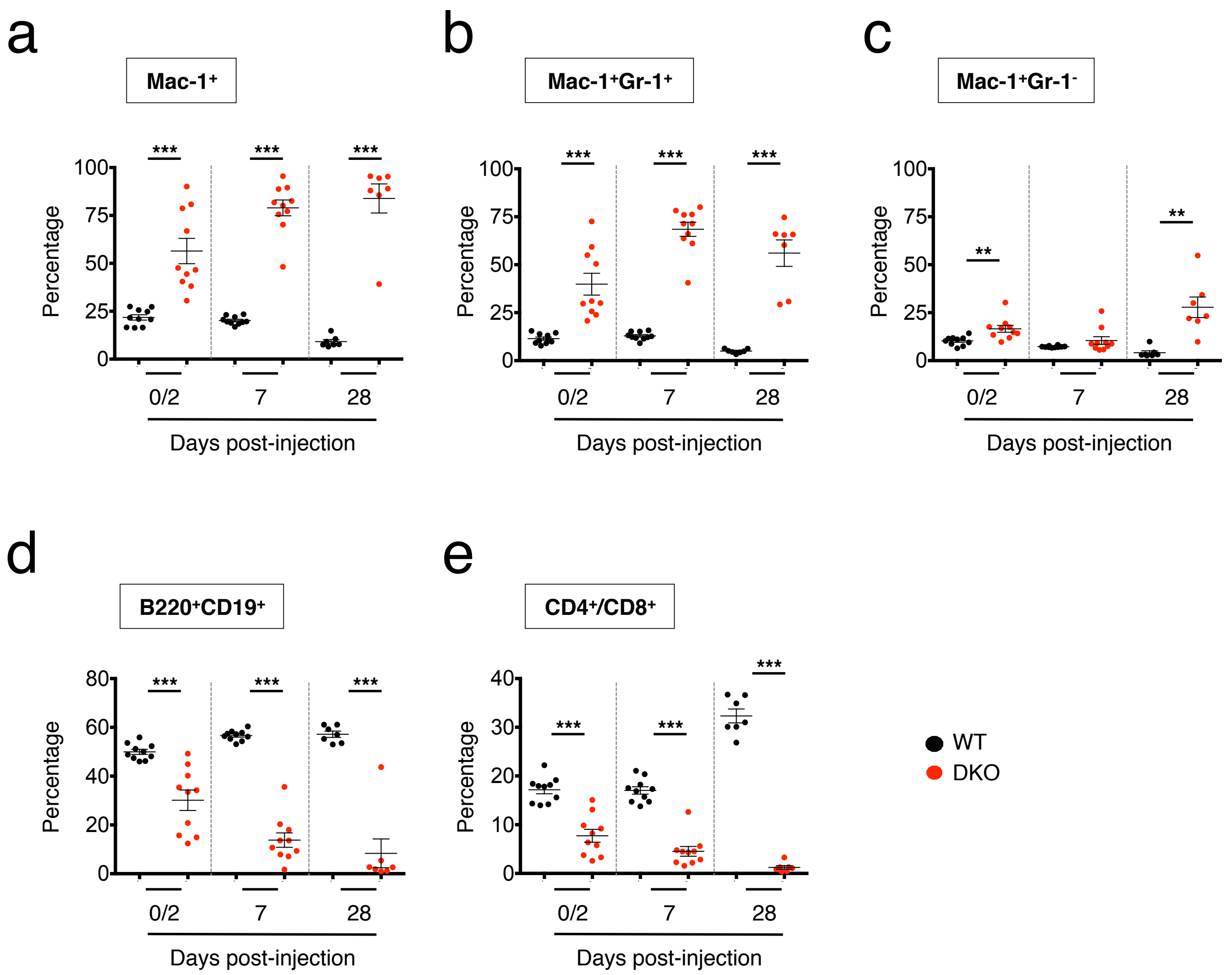

Supplementary Figure 3. Expansion of myeloid lineage cells with decrease in lymphoid lineage cells in *Tet2/3* DKO mice.
(**a-e**) Cells in the peripheral blood of WT or *Tet2/3* DKO mice were isolated at the indicated time points following pIpC injection and flow cytometric analysis of myeloid (**a-c**), B-cells (**d**) and T-cells (**e**) was performed (n = 7~10 mice per time point examined). Means ± SEM are shown. $^{**}P < 0.005$, $^{***}P < 0.0005$ (Student's *t* test).

Supplementary Fig. 4

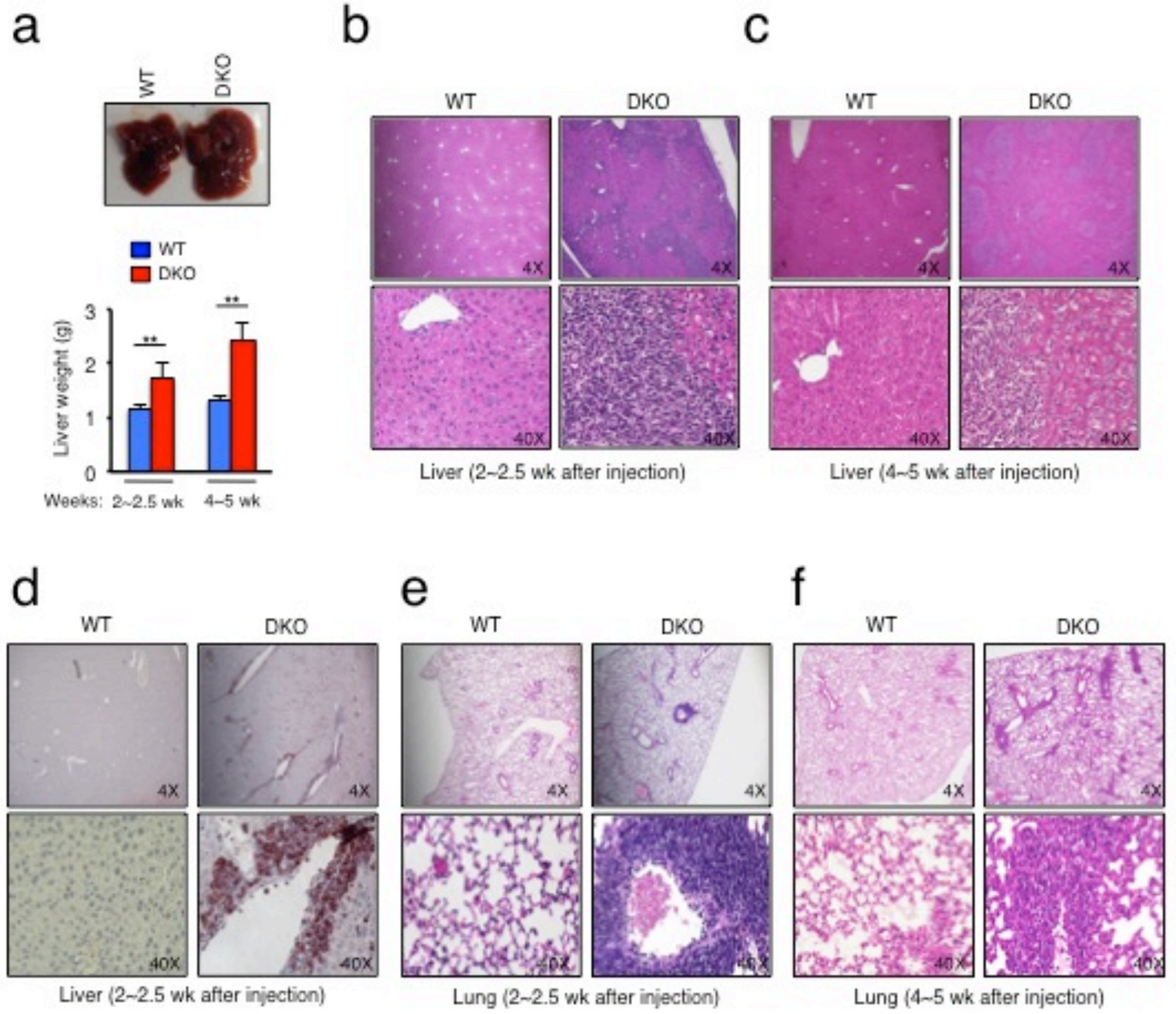

Supplementary Figure 4. Infiltration of livers and lungs of *Tet2/3* DKO mice with hematopoietic cells.
(a) Enlargement of livers in *Tet2/3* DKO mice. Representative photographs of liver from WT and *Tet2/3* DKO mice at 4~5 weeks following pIpC injection. Weights of liver at 2~2.5 or 4~5 weeks after pIpC injection were shown at bottom (n = 7~13 per each genotype). Means ± SEM are shown. $^{**}P < 0.005$, $^{***}P < 0.0005$ (Student's *t* test).
(b,c) Hematoxylin and eosin staining of livers at 2~2.5 (**b**) or 4~5 (**c**) weeks after pIpC injection. Shown are the loss of normal liver structure and hematopoietic cell infiltration into the livers of *Tet2/3* DKO mice.
(d) Myeloid cell infiltration into livers of *Tet2/3* DKO mice verified by myeloperoxidase staining at 2~2.5 weeks after pIpC injection.
(e,f) Histological analysis showing hematopoietic cell infiltration in the lung. Hematoxylin and eosin staining of lungs at 2~2.5 (**e**) and 4~5 (**f**) weeks after pIpC injection. For all figures, *top panels*, 4X magnification; *bottom panels*, 40X magnification.

Supplementary Fig. 5

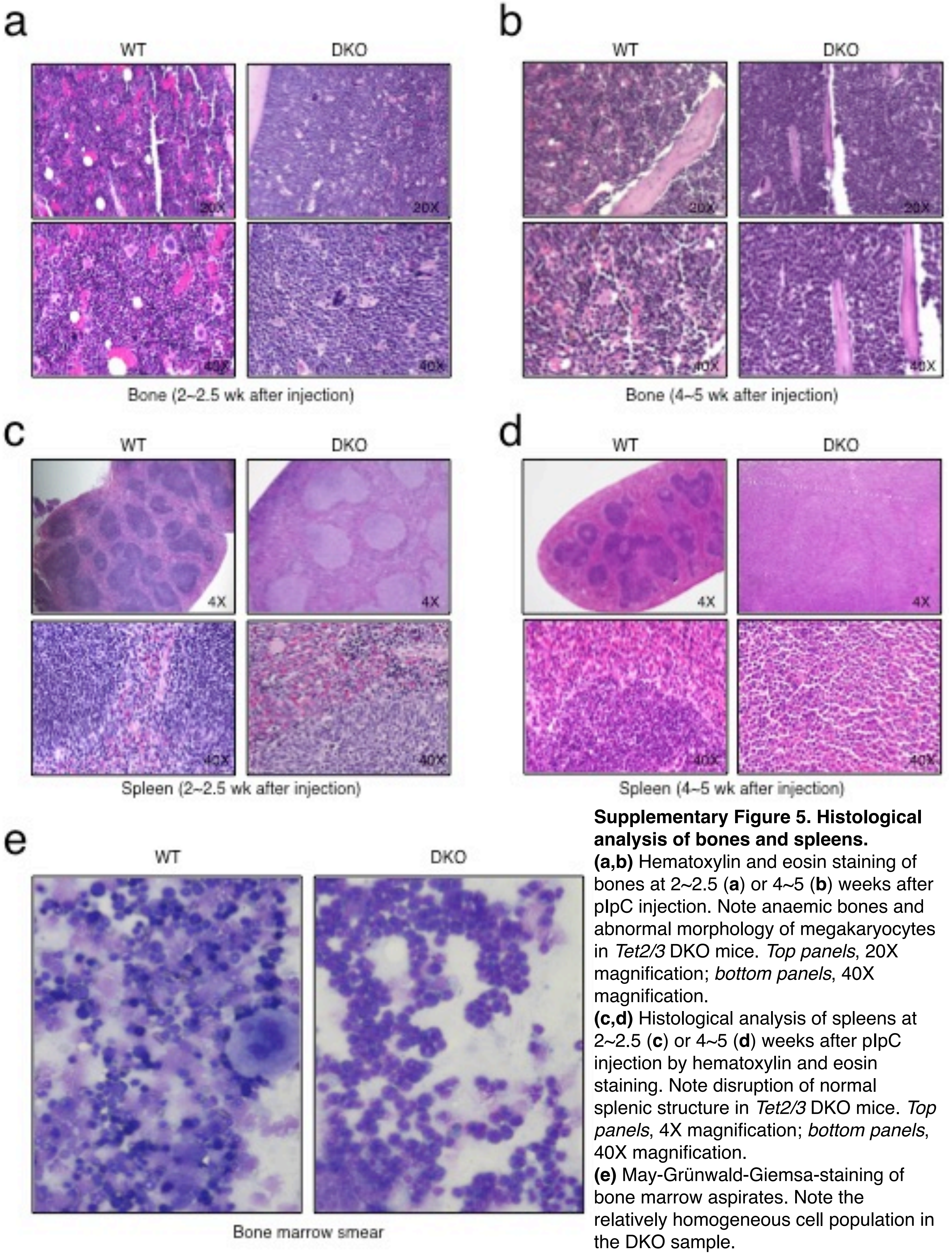

Supplementary Figure 5. Histological analysis of bones and spleens.
(a,b) Hematoxylin and eosin staining of bones at 2~2.5 (**a**) or 4~5 (**b**) weeks after pIpC injection. Note anaemic bones and abnormal morphology of megakaryocytes in *Tet2/3* DKO mice. *Top panels*, 20X magnification; *bottom panels*, 40X magnification.
(c,d) Histological analysis of spleens at 2~2.5 (**c**) or 4~5 (**d**) weeks after pIpC injection by hematoxylin and eosin staining. Note disruption of normal splenic structure in *Tet2/3* DKO mice. *Top panels*, 4X magnification; *bottom panels*, 40X magnification.
(e) May-Grünwald-Giemsa-staining of bone marrow aspirates. Note the relatively homogeneous cell population in the DKO sample.

Supplementary Fig. 6

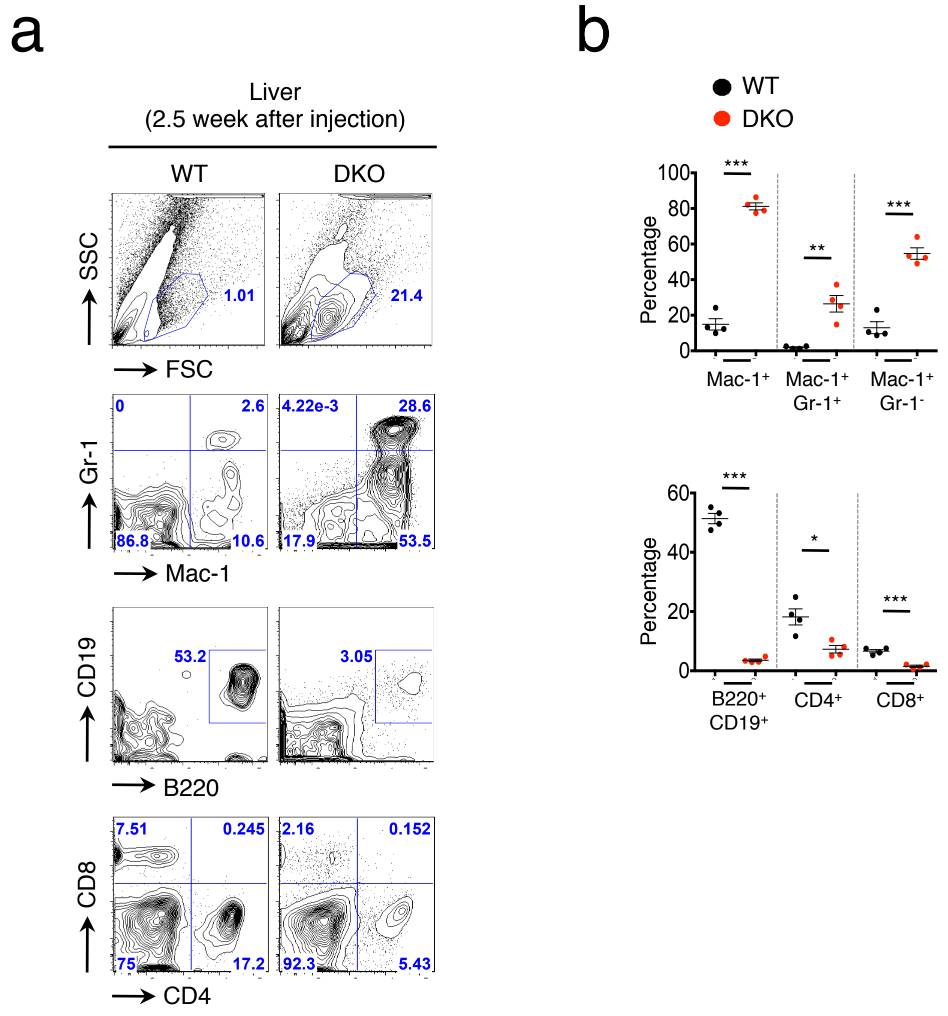

Supplementary Figure 6. Expansion of myeloid lineage cells and reduction of lymphoid cells in the liver of *Tet2/3* DKO mice.

(a) Flow cytometry was performed to assess myeloid (Gr-1/Mac-1), B lymphoid ($B220^+$ $CD19^+$) and T lymphoid (CD4/CD8) cell populations in the liver of WT or *Tet2/3* DKO mice at 2~2.5 weeks after pIpC injection. Hematopoietic cells were gated as shown in the top panel. Note expansion of FSC^{hi} and SSC^{hi} cells that normally correspond to myeloid cells in the liver of DKO mice.

(b) Summary of the percentage of cell populations shown in **a** (n = 4 per each genotype). Means ± SEM are shown. $^*P < 0.05$, $^{**}P < 0.005$, $^{***}P < 0.0005$ (Student's *t* test).

Supplementary Fig. 7

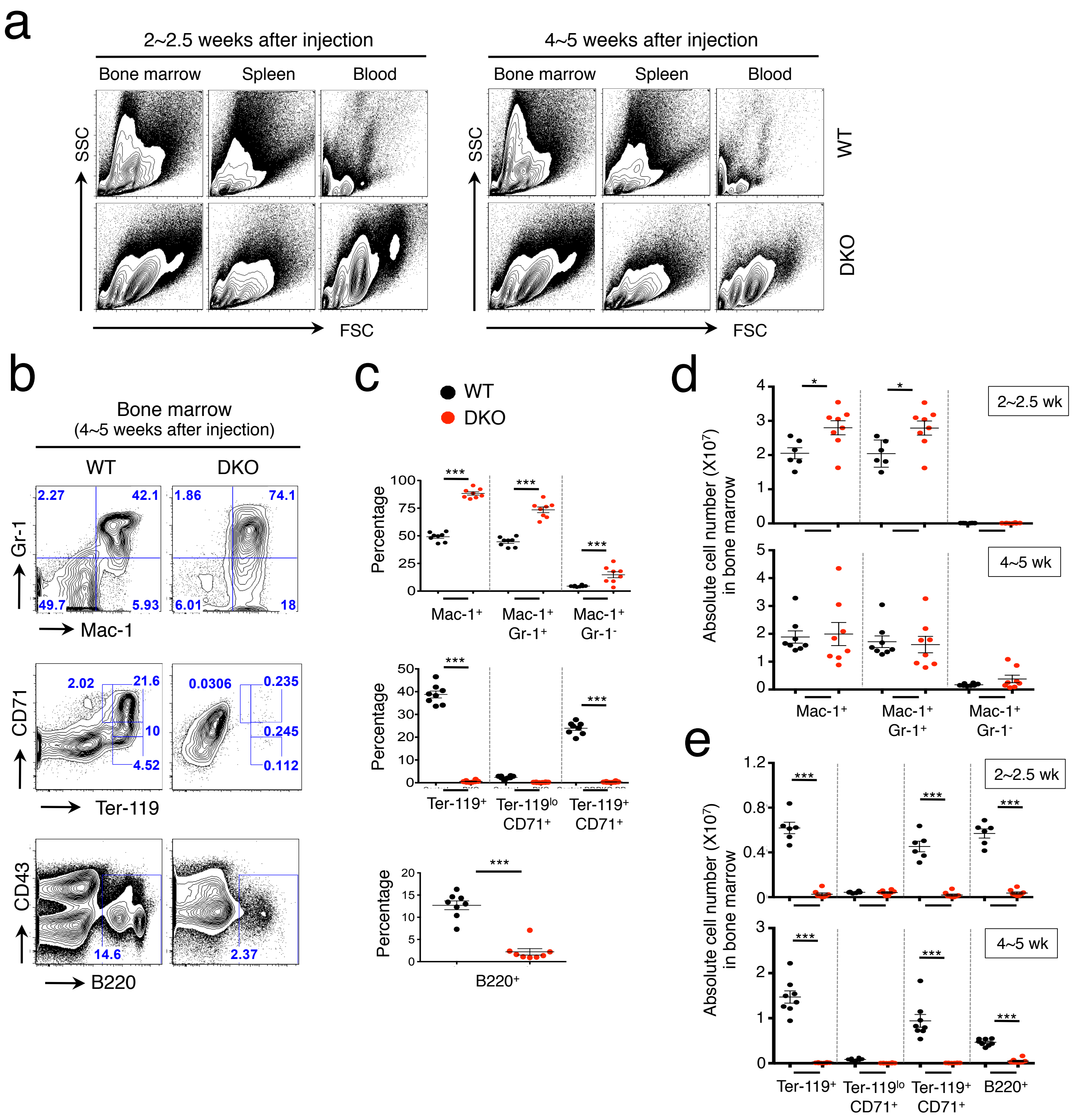

Supplementary Figure 7. Dominance of myeloid cells with reduction of erythroid cells and B cells in the bone marrow of *Tet2/3* DKO mice.

(a) Expansion of FSC^{hi} and SSC^{hi} cells in *Tet2/3* DKO mice. Relative heterogeneity (WT, *top*) or homogeneity (DKO, *bottom*) of cells in bone marrow, spleen and blood, as assessed by separating cells according to their side or forward scattering properties.

(b) Representative flow cytometry data assessing myeloid (Gr-1/Mac-1), erythroid (CD71/Ter-119) and B lymphoid (B220/CD43) cell populations in bone marrow of WT or DKO mice at 4~5 weeks after pIpC administration.

(c) The frequency of cells shown in **b**. Means ± SEM are shown. ***P* < 0.005, ****P* < 0.0005 (Student's *t* test).

(d,e) Absolute numbers of myeloid (**d**) or erythroid and lymphoid (**e**) cell subsets in bone marrow of WT or DKO mice at 2~2.5 or 4~5 weeks after pIpC injection. Means ± SEM are shown. **P* < 0.05, ***P* < 0.005, ****P* < 0.0005 (Student's *t* test).

Supplementary Fig. 8

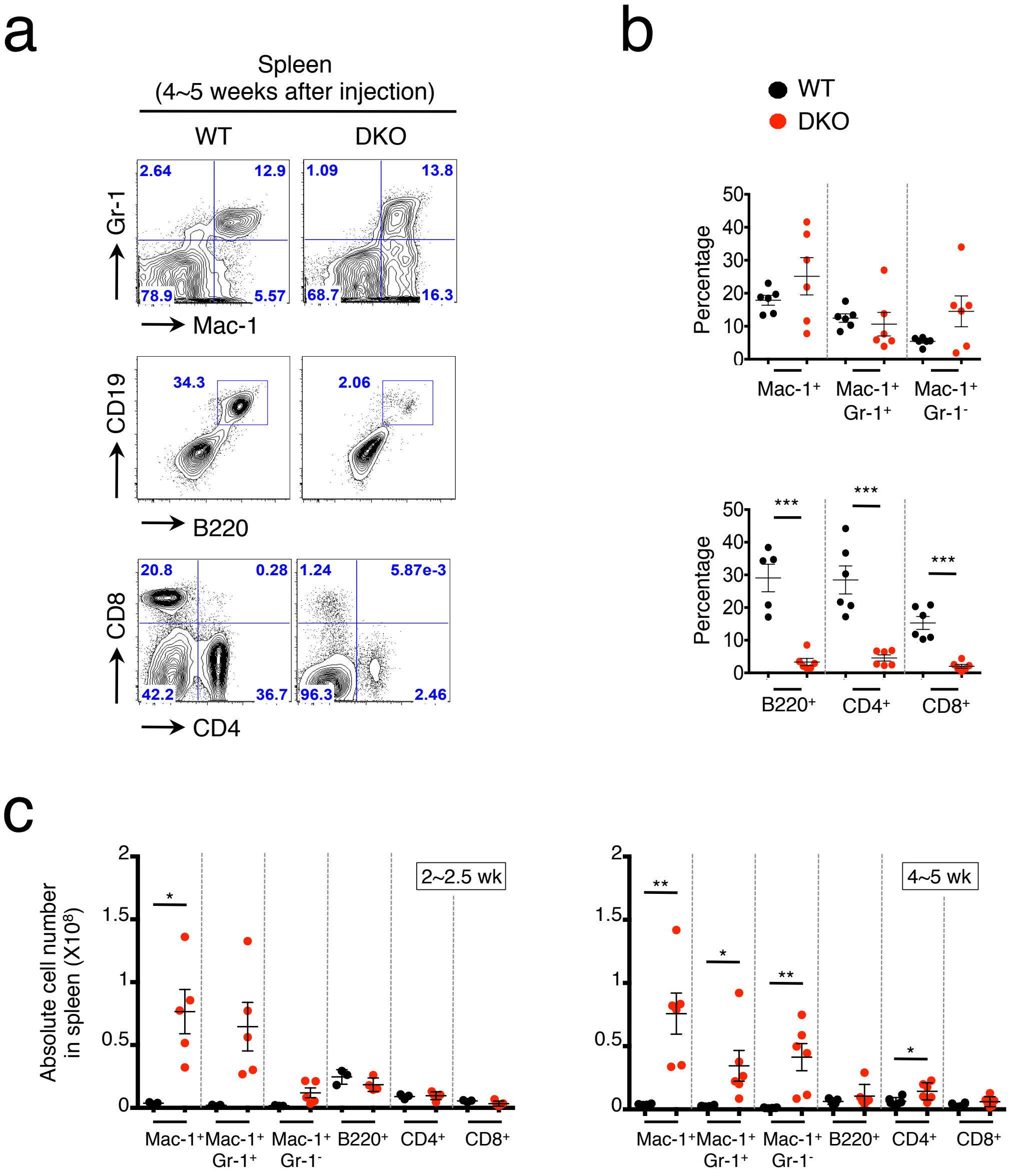

Supplementary Figure 8. Expansion of myeloid cells and reduction of lymphoid cells in the spleen of *Tet2/3* DKO mice.
(a) Representative flow cytometry data assessing myeloid (Gr-1/Mac-1), B lymphoid (B220/CD19) and T lymphoid (CD4/CD8) cell populations in spleens of WT or *Tet2/3* DKO mice at 4~5 weeks after pIpC administration.
(b) The frequency of cells shown in **a**. Means ± SEM are shown. *$P < 0.05$, ***$P < 0.0005$ (Student's *t* test).
(c) Absolute numbers of myeloid and lymphoid cell subsets in spleens of WT or *Tet2/3* DKO mice at 2~2.5 (*left*) or 4~5 (*right*) weeks after pIpC injection. Means ± SEM are shown. *$P < 0.05$, **$P < 0.005$, ***$P < 0.0005$ (Student's *t* test).

Supplementary Fig. 9

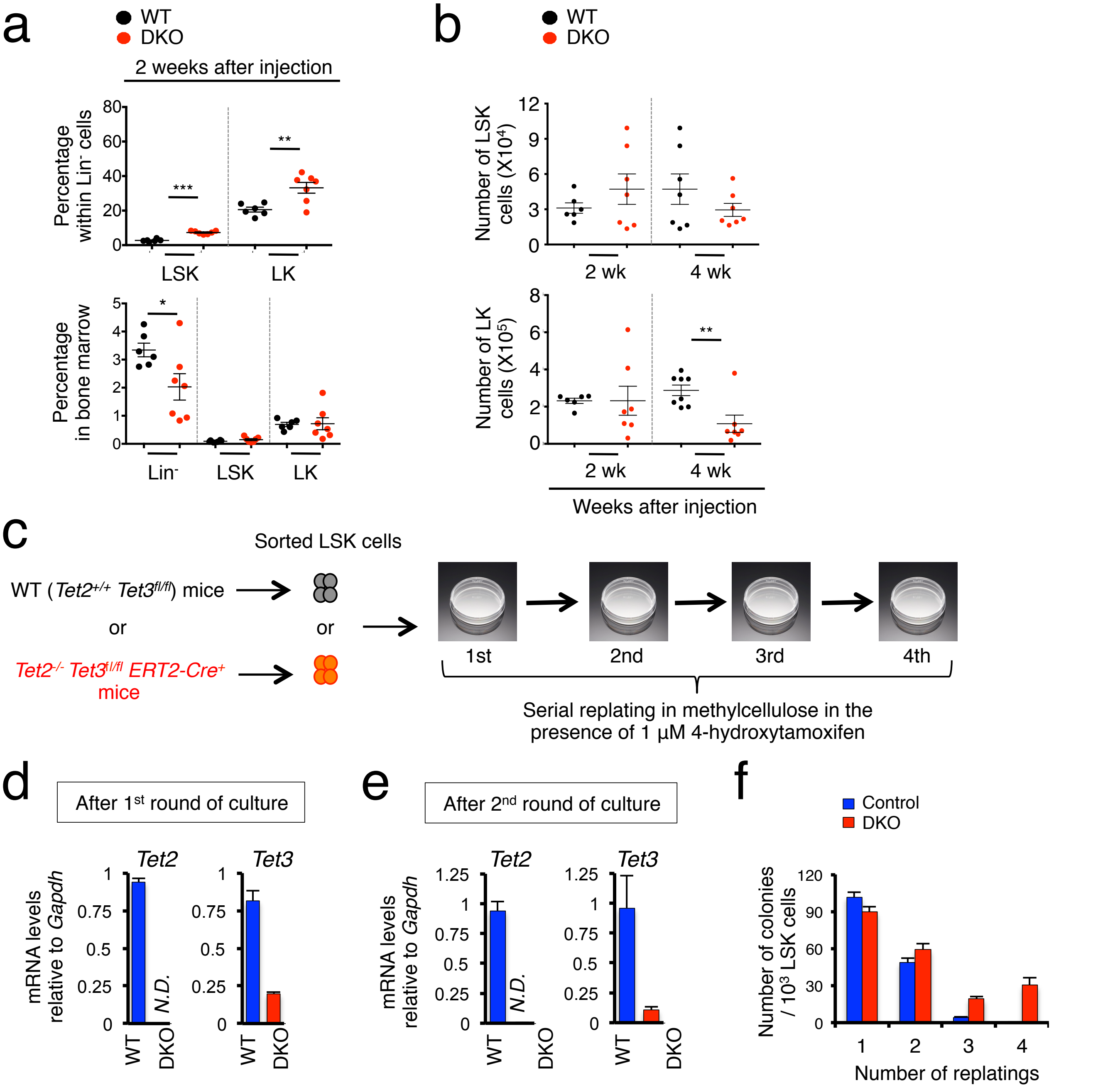

Supplementary Figure 9. Altered distribution of hematopoietic stem and progenitor cells upon acute deletion of *Tet3* in *Tet2*-deficient mice.

(a) Frequency of LSK (Lin^- $c\text{-}Kit^+$ $Sca1^+$) and LK (Lin^- $c\text{-}Kit^+$ $Sca1^-$) cells within Lin^- populations (*top*) or in the total bone marrow (*bottom*) of WT and *Tet2/3* DKO mice at 2 weeks after pIpC injection. Means ± SEM are shown. $^*P < 0.05$, $^{**}P < 0.005$, $^{***}P < 0.0005$ (Student's *t* test).

(b) Absolute numbers of LSK and LK cells in bone marrow of WT and *Tet2/3* DKO mice at 2 or 4 weeks after pIpC injection. Means ± SEM are shown. $^{**}P < 0.005$ (Student's *t* test).

(c) Experimental scheme for colony-forming assay in methylcellulose medium. LSK cells were sorted from bone marrow of WT (*Tet3*$^{fl/fl}$) or DKO (*Tet2*$^{-/-}$ *Tet3*$^{fl/fl}$ *ERT2-Cre*$^{+}$) mice by flow cytometry (n = 4 per each genotype). One thousand cells were serially replated in methylcellulose medium (MethoCult M3534, Stem Cell Technologies) and colonies were counted after 7-9 days. To induce *ERT2-Cre* recombinase, 1 µM 4-hydroxytamoxifen was added to the cultures.

(d,e) Confirmation of *Tet2* and *Tet3* deletion. Colonies after the first (**d**) or second (**e**) round of culture were pooled and quantitative RT-PCR analysis was performed to confirm germline deletion of *Tet2* and *ERT2-Cre*-mediated deletion of *Tet3*.

(f) Combined deficiency of Tet2 and Tet3 leads to increased serial replating capacity in vitro. Colony forming unit assay shows that *Tet2/3*-deficient, but not WT, LSK cells can be serially replated in methylcellulose medium.

Supplementary Fig. 10

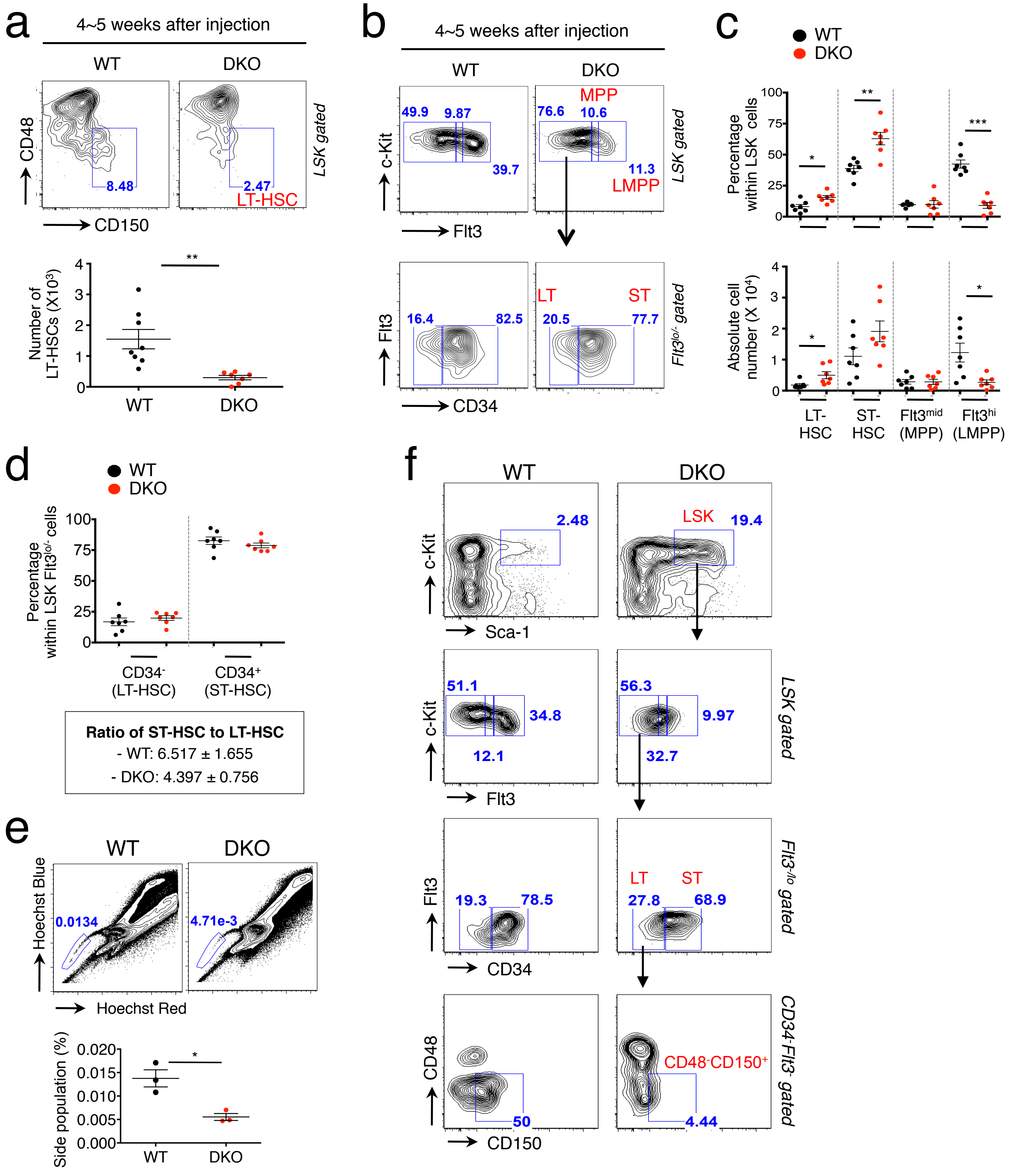

Supplementary Figure 10. Altered distribution of hematopoietic stem/progenitor cells upon acute deletion of *Tet3* in *Tet2*-deficient mice.

Supplementary Figure 10. Altered distribution of hematopoietic stem/progenitor cells upon acute deletion of *Tet3* in *Tet2*-deficient mice.

(a) Acute deletion of *Tet3* in *Tet2*-deficient mice decreases the number of SLAM-marked hematopoietic stem cells (LSK $CD150^+$ $CD48^-$). LSK cells in the bone marrow from WT and DKO mice were analyzed for surface expression of CD48 and CD150. Means ± SEM are shown. $^{**}P < 0.005$ (Student's t test).

(b) LSK cells in bone marrow of WT and *Tet2/3* DKO mice were analyzed for surface expression of c-Kit and Flt3, after which the $Flt3^{-/lo}$ fraction was further analyzed based on CD34 expression. $CD34^-$ and $CD34^+$ cells in the lower panels represent LT- and ST-HSCs, respectively.

(c) Percentage (*upper panel*) and number (*lower panel*) of LT-HSCs, ST-HSCs, MPPs and LMPPs in WT and DKO mice. Means ± SEM are shown. $^{*}P < 0.05$, $^{***}P < 0.0005$ (Student's t test).

(d) Deficiency of Tet2 and Tet3 results in only a slight decrease in the ratio of ST-HSC to LT-HSCs.

(e) Hoechst staining and flow cytometric analysis of the bone marrow of WT and DKO mice. The boxed region in the upper panel indicates the percentage of side population cells, and summary of the results (mean ± SEM) is shown in the lower panel.

(f) Increased frequency of $CD48^+$ $CD150^-$ population within LT-HSCs ($CD34^-Flt3^-$ LSK) in DKO compared to WT mice.

Supplementary Fig. 11

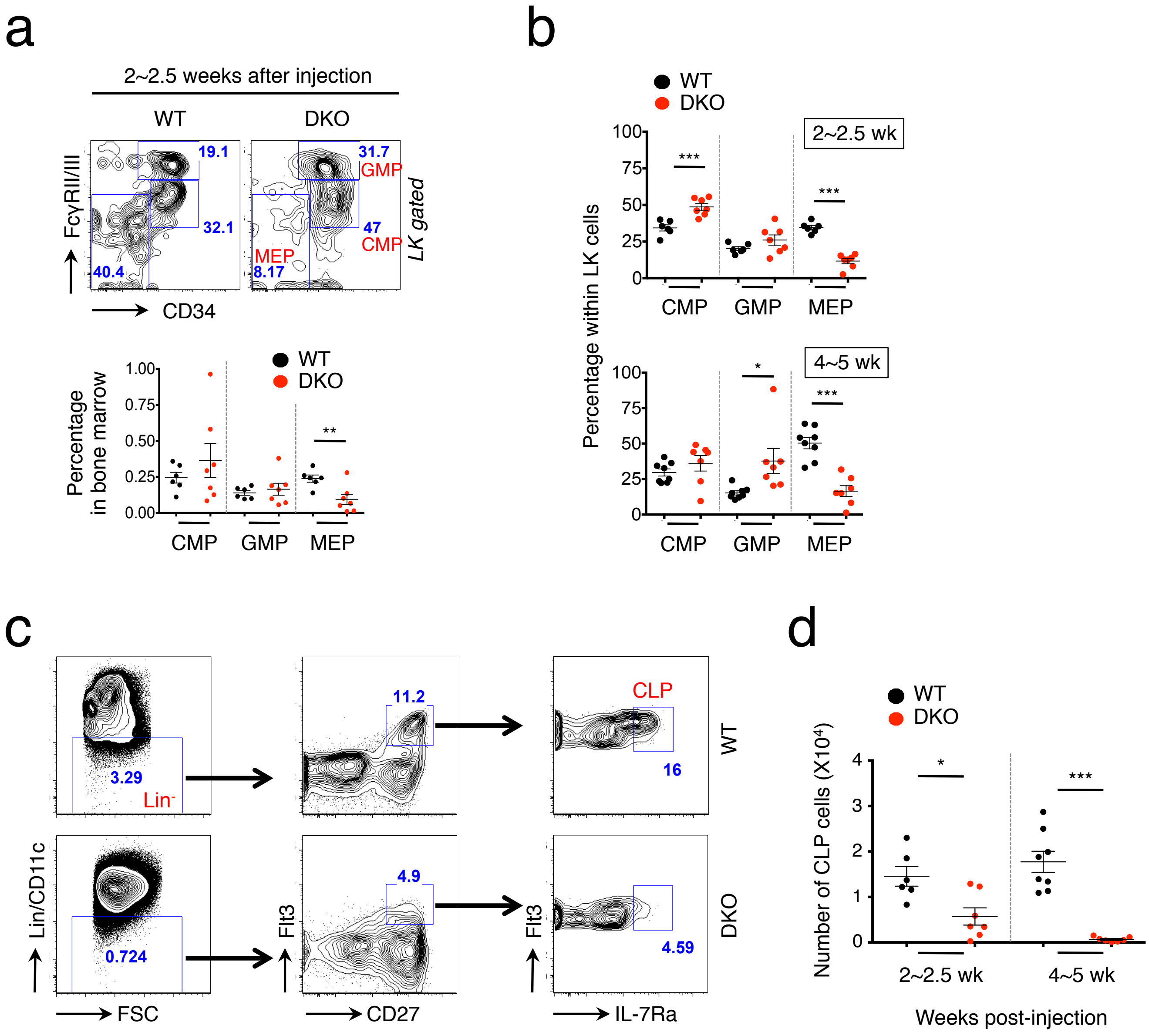

Supplementary Figure 11. Distribution of myeloid and lymphoid progenitors in the bone marrow.
(a) Representative flow cytometric analysis of myeloid progenitor cell subsets in the bone marrow of WT and *Tet2/3* DKO mice at 2~2.5 weeks after pIpC injection, as assessed by the expression of FcγRII/III and CD34 within LK population (*top*). Percentage of each myeloid progenitor in bone marrow is shown at *bottom*. Means ± SEM are shown. $^{**}P < 0.005$ (Student's *t* test).
(b) Frequency of myeloid progenitor cell subsets within LK populations of WT and *Tet2/3* DKO mice at 2~2.5 or 4~5 weeks after pIpC injection. Means ± SEM are shown. $^{*}P < 0.05$, $^{***}P < 0.0005$ (Student's *t* test).
(c) Gating strategy to identify common lymphoid progenitor (CLP) cells in bone marrow. CLPs are defined as Lin^- $Flt3^+$ $CD27^+$ IL-$7R\alpha^+$.
(d) Absolute number of CLPs in the bone marrow of WT and *Tet2/3* DKO mice at 2~2.5 or 4~5 weeks after pIpC injection. Means ± SEM are shown. $^{*}P < 0.05$, $^{***}P < 0.0005$ (Student's *t* test).

Supplementary Fig. 12

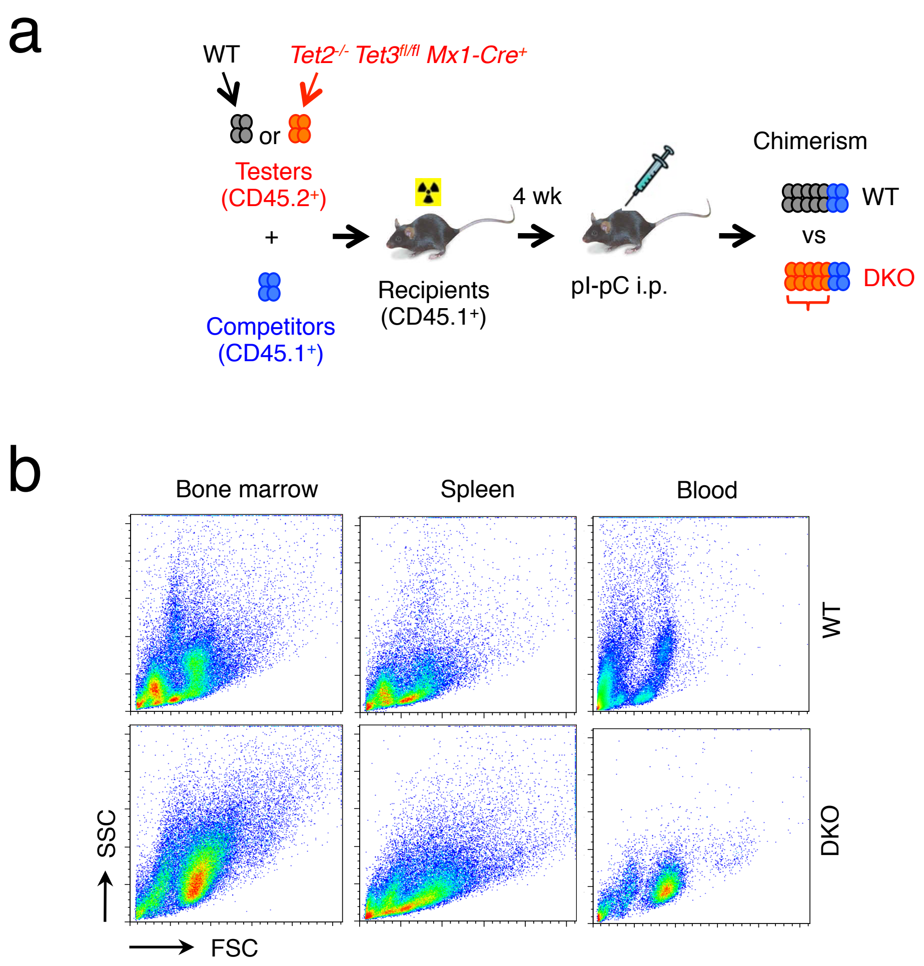

Supplementary Figure 12. Competitive engraftment assay and tumor transfer assay.
(a) Experimental scheme for competitive repopulation assay. CD45.2^{+} bone marrow cells from *Tet2*$^{+/+}$ *Tet3*$^{fl/fl}$ *Mx1-Cre*$^{-}$ (WT) or *Tet2*$^{-/-}$ *Tet3*$^{fl/fl}$ *Mx1-Cre*$^{+}$ (DKO) mice were mixed with equal number of CD45.1^{+} competitor cells and transplanted into lethally irradiated CD45.1^{+} congenic mice. At 4 weeks after transplantation, chimeric mice were injected with pIpC five times intraperitoneally (week 0) and peripheral blood was examined for donor chimerism at the indicated time.
(b) FSC^{hi} and SSC^{hi} cells in the recipients of WT and DKO splenocytes. Relative heterogeneity (WT, *top*) or homogeneity (DKO, *bottom*) of cells in the bone marrow, spleen, blood, as assessed by separating cells according to their side or forward scattering properties.

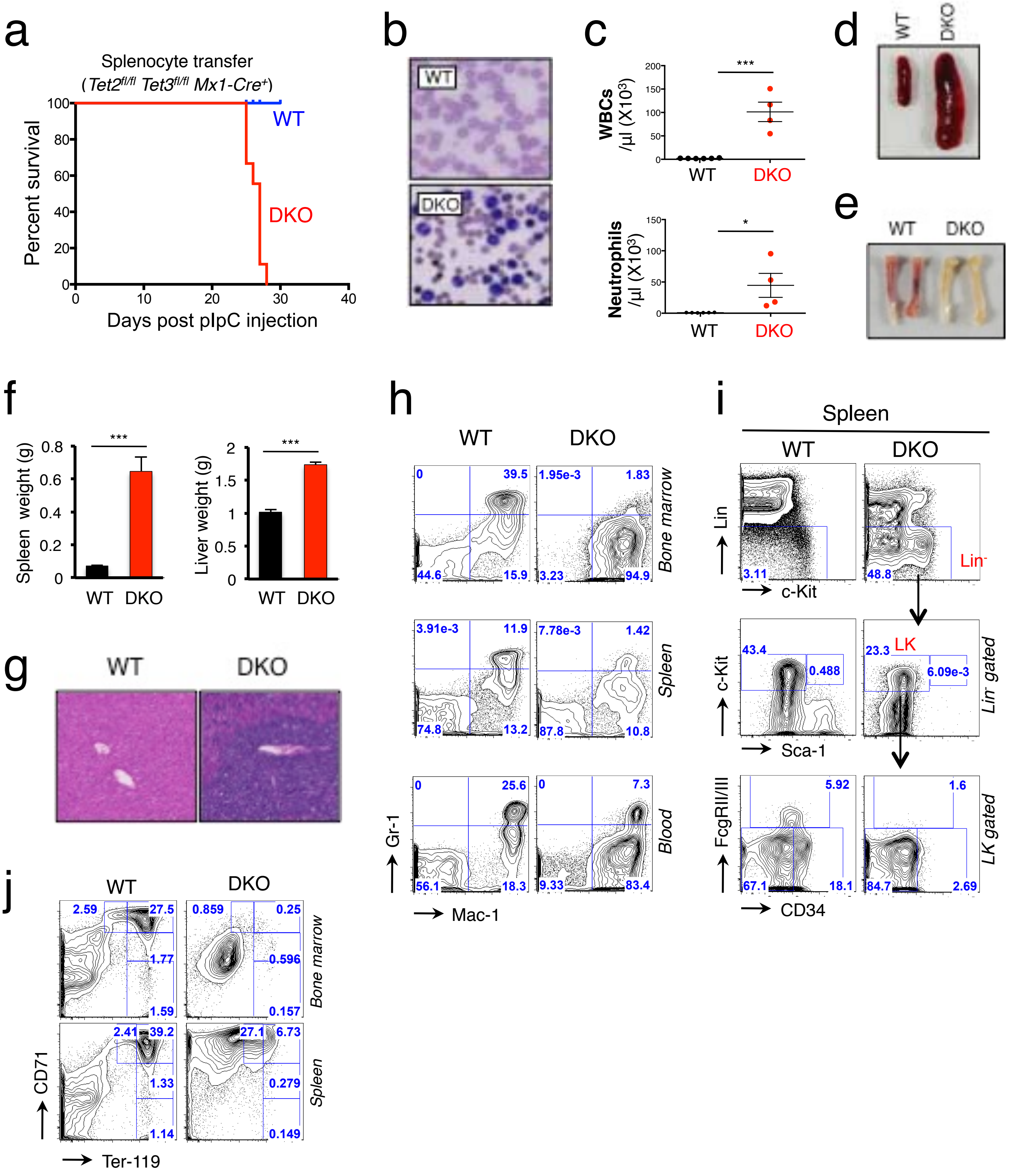

Supplementary Figure 13. Transfer of splenocytes from *Tet2/3* DKO mice leads to myeloid leukemia in secondary recipients.

Supplementary Figure 13. Transfer of splenocytes from *Tet2/3* DKO mice leads to myeloid leukemia in secondary recipients.
(a) Kaplan-Meier curve representing the percent survival of recipient mice transplanted with 2 x 10^6 splenocytes from WT (*Tet2*$^{fl/fl}$ *Tet3*$^{fl/fl}$) and diseased *Tet2/3* DKO (*Tet2*$^{fl/fl}$ *Tet3*$^{fl/fl}$ *Mx1-Cre*$^{+}$) mice (n = 10 per each group).
(b) May-Grünwald-Giemsa-stained peripheral blood smears of recipient mice.
(c) Recipients of *Tet2/3* DKO splenocytes developed progressive leukocytosis with neutrophilia. Means ± SEM are shown. $^{*}P < 0.05$, $^{***}P < 0.0005$ (Student's *t* test). For a summary of other hematopoietic parameters, see **Supplementary Table 2b**.
(d) Enlargement of spleens of mice that received *Tet2/3* DKO splenocytes.
(e) Representative photographs of femurs and tibiae from recipients of WT or *Tet2/3* DKO splenocytes.
(f) Weights of spleen or liver. Means ± SEM are shown. $^{***}P < 0.0005$ (Student's *t* test).
(g) Hematoxylin and eosin staining of livers show loss of normal liver structure and infiltration with hematopoietic cells. 4X magnification.
(h) A representative flow cytometric analysis of myeloid-lineage cells (Gr-1^{+}/Mac-1^{+}) in the bone marrow, spleen and blood of recipient mice.
(i) Increase in the frequency of Lin^{-} populations in the spleen from recipients of *Tet2/3* DKO splenocytes.
(j) A representative flow cytometric analysis of erythroid-lineage cells (Ter-119^{+}/CD71^{+}) in the bone marrow and spleen of recipient mice.

Supplementary Fig. 14

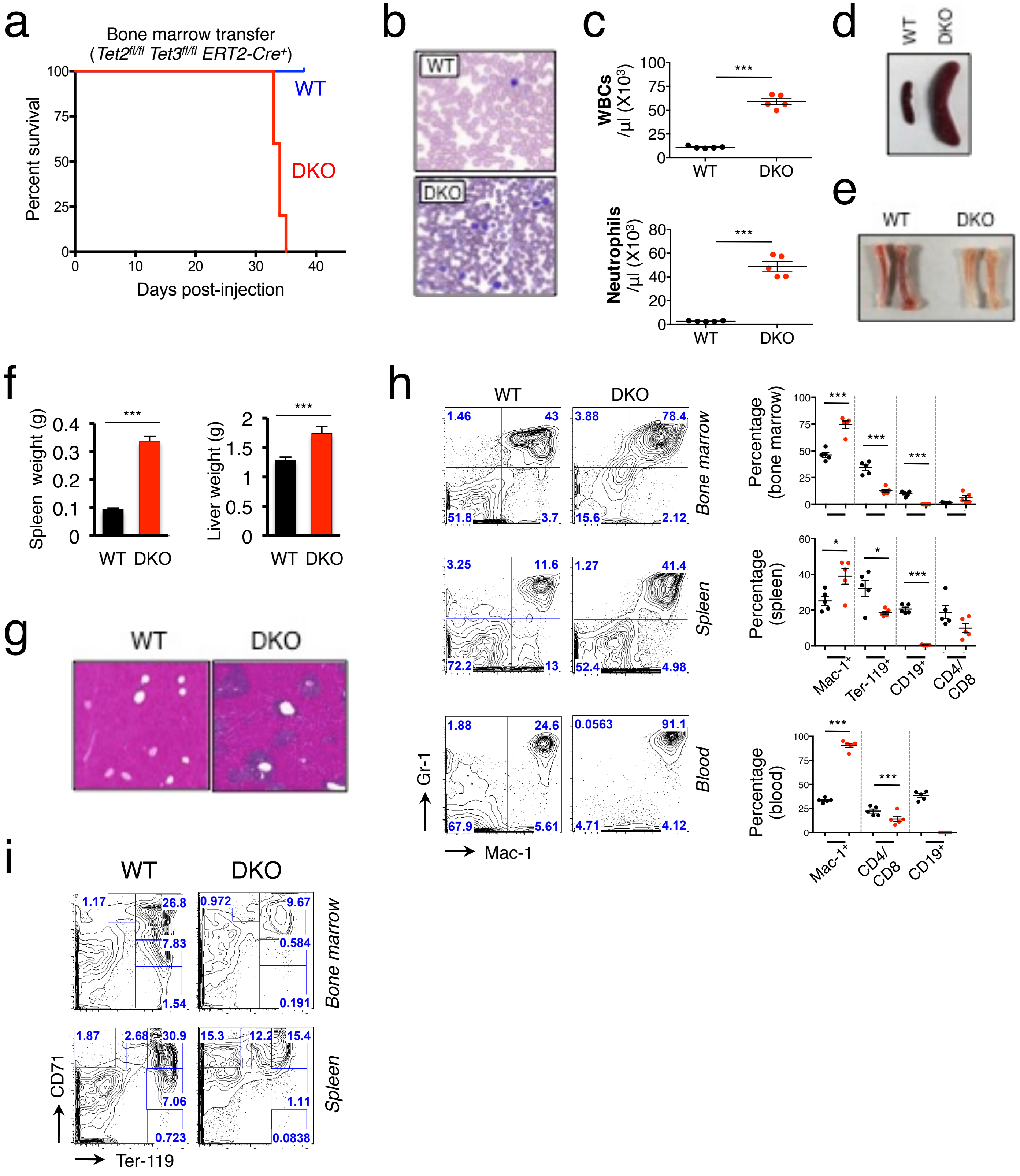

Supplementary Figure 14. Transfer of bone marrow cells from *Tet2/3* DKO mice leads to myeloid leukemia in secondary recipients.

Supplementary Figure 14. Transfer of bone marrow cells from *Tet2/3* DKO mice leads to myeloid leukemia in secondary recipients.

(a) Kaplan-Meier curve representing the percent survival of recipient mice transplanted with 2 x 10^6 nucleated bone marrow cells from WT (*Tet2$^{fl/fl}$ Tet3$^{fl/fl}$*) and diseased *Tet2/3* DKO (*Tet2$^{fl/fl}$ Tet3$^{fl/fl}$ ERT2-Cre^{+}*) mice (n = 5 per each group).

(**b**) May-Grünwald-Giemsa-stained peripheral blood smears of recipient mice.

(c) Recipients of *Tet2/3* DKO bone marrow cells developed progressive leukocytosis with neutrophilia. Means ± SEM are shown. ***$P < 0.0005$ (Student's *t* test). For a summary of other hematopoietic parameters, see **Supplementary Table 2c**

(d) Enlargement of spleens of mice that received *Tet2/3* DKO bone marrow cells.

(e) Representative photographs of femurs and tibiae from recipients of WT or *Tet2/3* DKO bone marrow cells.

(f) Weights of spleen or liver. Means ± SEM are shown. ***$P < 0.0005$ (Student's *t* test).

(g) Hematoxylin and eosin staining of livers show loss of normal liver structure and infiltration with hematopoietic cells. 4X magnification.

(h) A representative flow cytometric analysis of myeloid-lineage cells (Gr-1^{+}/Mac-1^{+}) in the bone marrow, spleen and blood of recipient mice. Summary of the percentage of each cell population is also shown in the right panel.

(i) A representative flow cytometric analysis of erythroid-lineage cells (Ter-119^{+}/CD71^{+}) in the bone marrow and spleen of recipient mice.

Supplementary Fig. 15

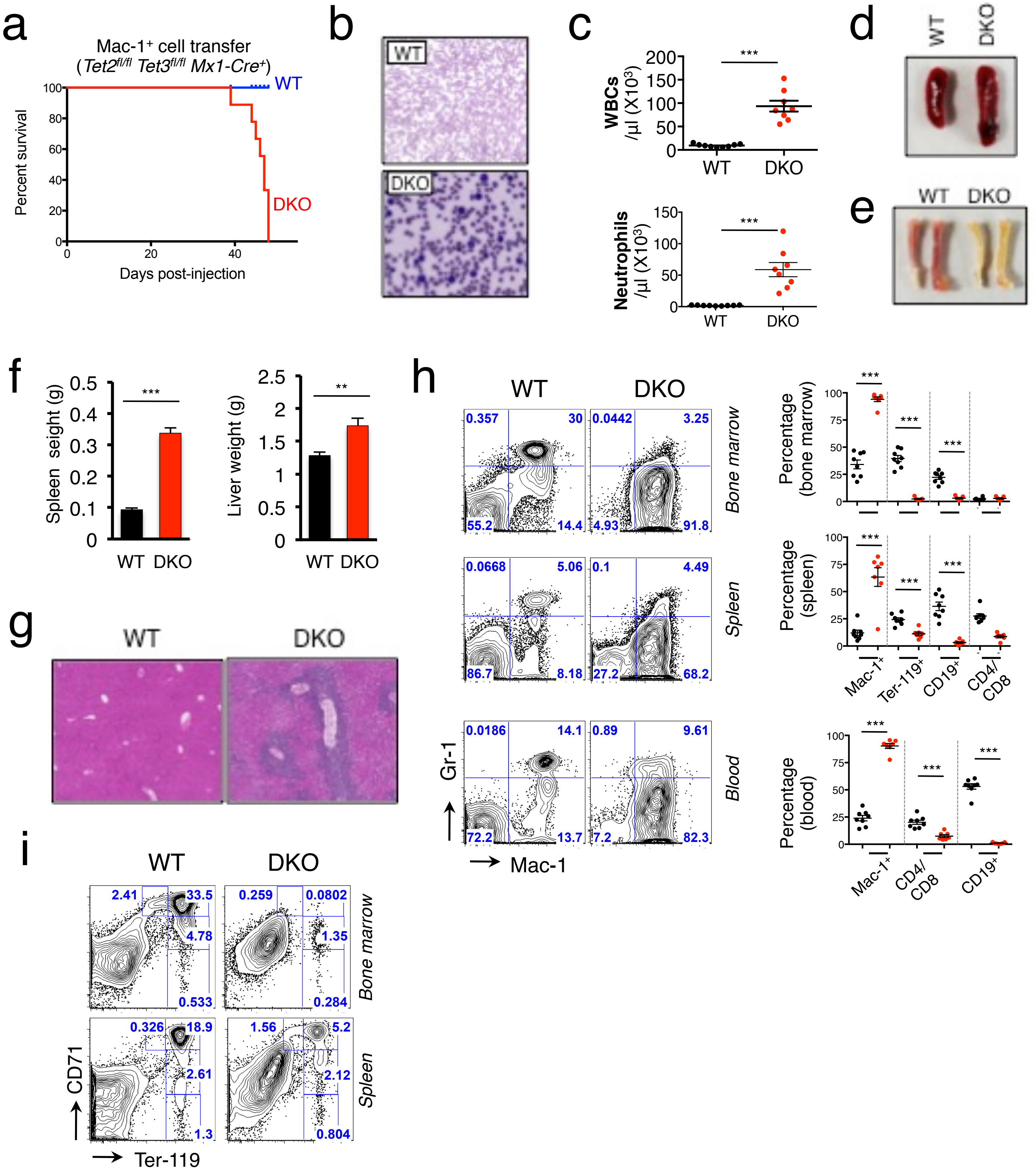

Supplementary Figure 15. Transfer of Mac-1⁺ cells from *Tet2/3* DKO mice leads to myeloid leukemia in secondary recipients.

Supplementary Figure 15. Transfer of Mac-1+ cells from *Tet2/*3 DKO mice leads to myeloid leukemia in secondary recipients.

(a) Kaplan-Meier curve representing the percent survival of recipient mice transplanted with 2 x 10^6 Mac-1$^+$ cells from WT (*Tet2$^{fl/fl}$ Tet3$^{fl/fl}$*) and diseased *Tet2/3* DKO (*Tet2$^{fl/fl}$ Tet3$^{fl/fl}$ Mx1-Cre$^+$*) mice (n = 9 per each group).

(b) May-Grünwald-Giemsa-stained peripheral blood smears of recipient mice.

(c) Recipients of *Tet2/3* DKO Mac-1$^+$ cells developed progressive leukocytosis with neutrophilia. Means ± SEM are shown. ****P* < 0.0005 (Student's *t* test). For a summary of other hematopoietic parameters, see **Supplementary Table 2d**.

(d) Enlargement of spleens of mice that received *Tet2/3* DKO Mac-1$^+$ cells.

(e) Representative photographs of femurs and tibiae from recipients of WT or *Tet2/3* DKO Mac-1$^+$ cells.

(f) Weights of spleen or liver. Means ± SEM are shown. ****P* < 0.0005 (Student's *t* test).

(g) Hematoxylin and eosin staining of livers show loss of normal liver structure and infiltration with hematopoietic cells. 4X magnification.

(h) A representative flow cytometric analysis of myeloid-lineage cells (Gr-1$^+$/Mac-1$^+$) in the bone marrow, spleen and blood of recipient mice. Summary of the percentage of each cell population is also shown in the right panel.

(i) A representative flow cytometric analysis of erythroid-lineage cells (Ter-119$^+$/CD71$^+$) in the bone marrow and spleen of recipient mice.

Supplementary Fig. 16

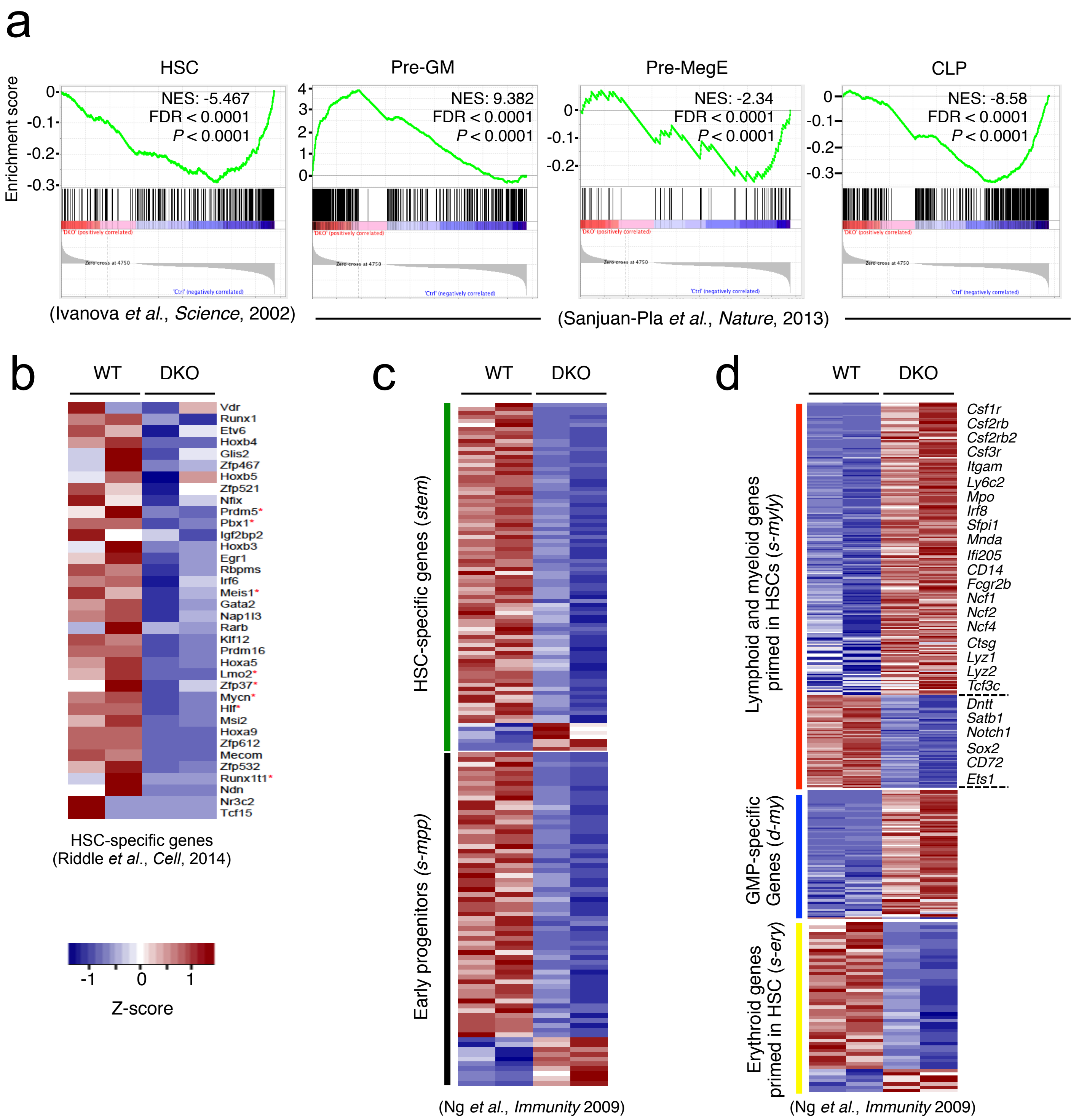

Supplementary Figure 16. Tet2 and Tet3 differentially modulate stem cell- and lineage-affiliated transcriptional programs.

Supplementary Figure 16. Tet2 and Tet3 differentially modulate stem cell- and lineage-affiliated transcriptional programs.

a, Gene set enrichment analysis (GSEA) of RNA-Seq data shows that the pre-granulocyte/ macrophage progenitor (pre-GM) gene signature is significantly enriched for genes up-regulated in *Tet2/3* DKO compared with WT LSK cells, whereas the HSC, pre-megakaryocyte/ erythrocyte progenitor (Pre-MegE) and common lymphoid progenitor (CLP) gene signatures are enriched in genes downregulated in *Tet2/3* DKO compared with WT LSK cells.

b-d, Heatmap representation showing differential expression of genes highly expressed in HSC and their immediate downstream progenitors (**b, c)**, as well as lineage-affiliated genes (**d**) in *Tet2/3* DKO LSK cells compared with WT LSK cells. The gene sets (including *stem*, *s-mpp*, *s-ery*, *d-my* and *s-myly*) have been described. Only genes with a p-value ≤ 0.05 and fold change > 1.5 or < 0.67 were considered. Asterisks in **b** indicate genes used to induce reprogramming of differentiated hematopoietic cells into induced HSCs. The color key for all heatmaps indicates row-wise scaled RPKM values (z-score).

Supplementary Fig. 17

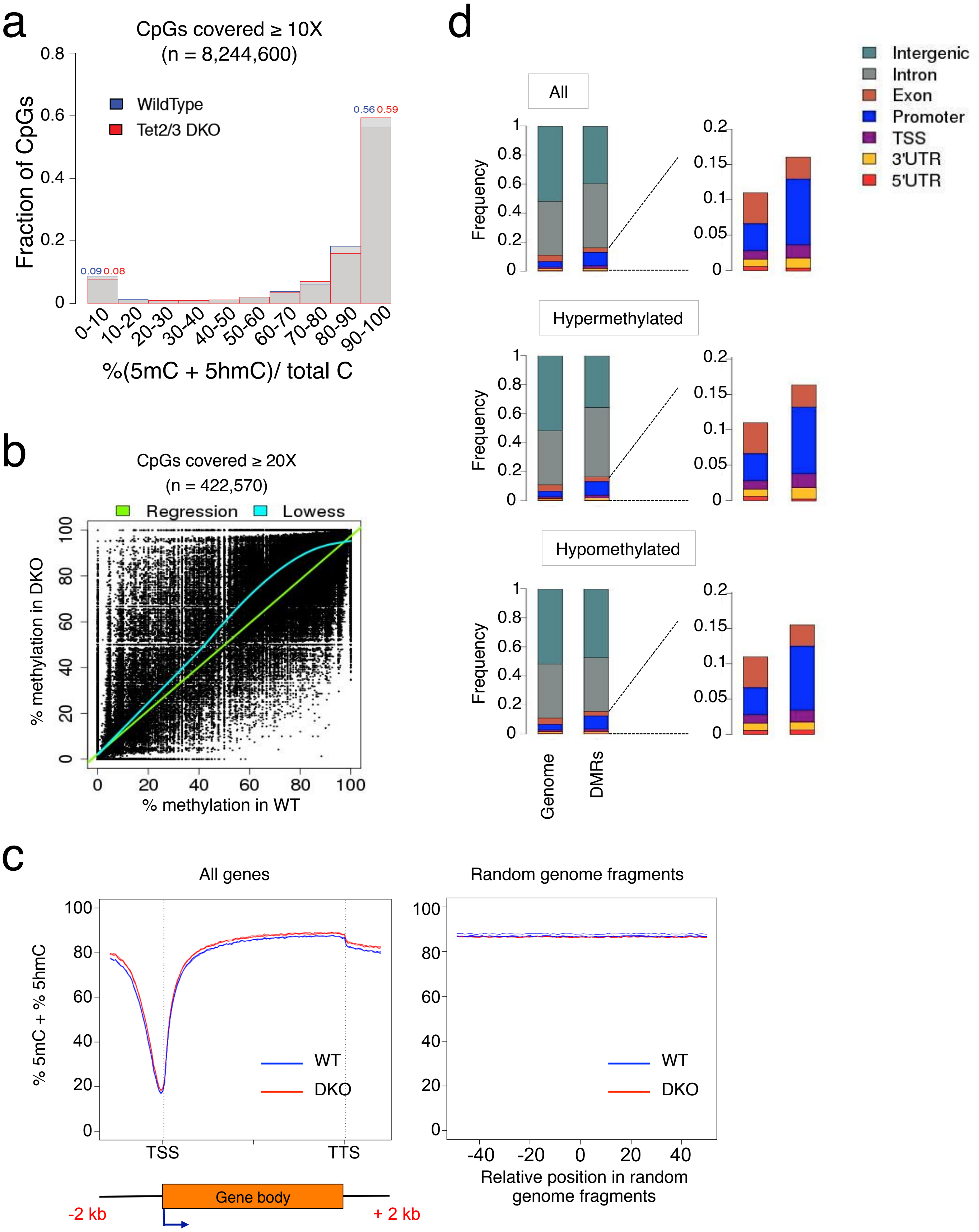

Supplementary Figure 17. Loss of Tet2 and Tet3 results in aberrant gene expression and DNA methylation.

Supplementary Figure 17. Loss of Tet2 and Tet3 results in aberrant gene expression and DNA methylation.
a, Histogram of genome-wide CpG modification (5mC + 5hmC) in WT (*blue*) and *Tet2/3* DKO (*red*) LSK cells at CpGs covered by at least ten reads. The histogram shows a typical bimodal distribution where the majority of CpGs are highly methylated.
b, Scatter plot of genome-wide CpG methylation comparing WT LSK (x-axis) and *Tet2/3* DKO LSK (y-axis) cells at individual CpGs (*black dots*) covered by at least 20 reads in each condition. The graph indicates the genome-wide increase of DNA methylation in *Tet2/3* DKO LSK cells, and the Lowess curve confirms that the majority of CpGs in both WT and DKO genomes are methylated.
c, *Left*, Average DNA methylation (5mC+5hmC) along gene regions, including 2 kb upstream of the transcription start sites (TSS) and 2 kb downstream of the transcription termination sites (TTS). Considering all genes, the average level of DNA methylation is slightly but consistently increased in *Tet2/3* DKO LSK samples (*red lines*) compared to the control LSK samples (*blue lines*); note the close correlation between the triplicate biological samples in each case. Similar results were obtained when considering upregulated or downregulated genes (see **Figure 5d**). *Right*, Average DNA methylation (5mC+5hmC) in randomly chosen genome fragments of similar size.
d, DMRs are significantly enriched in promoters and exons.

Supplementary Fig. 18

a

Total no. of promoter CpG islands (CGIs)	12005
**No. of CGIs more methylated in DKO**	**171 (1.42%)**
Upregulated	3
Downregulated	7
**No. of CGI shores more methylated in DKO**	**1539**
Upregulated	116
Downregulated	135
**No. of CGIs less methylated in DKO**	**53 (0.44%)**
Upregulated	5
Downregulated	3
**No. of CGI shores less methylated in DKO**	**224**
Upregulated	20
Downregulated	16

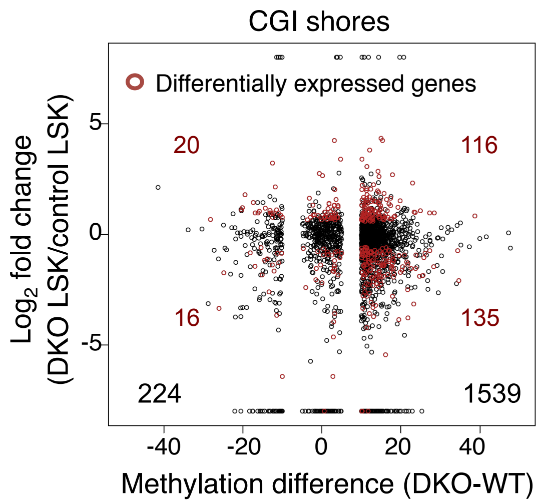

b

**Total no. of canyons**	**1,135**
**No. of canyons close to a gene**	**846 (74.54%)**
**- Upregulated**	**28**
**- Downregulated**	**77**
**No. of canyons that shrink in DKO**	**465 (40.97%)**
Genes associated with canyons that shrink	525
- Upregulated	19
- Downregulated	67
**No. of canyons that expand in DKO**	**59 (5.20%)**
Genes associated with expanded canyons	70
- Upregulated	4
- Downregulated	3

c

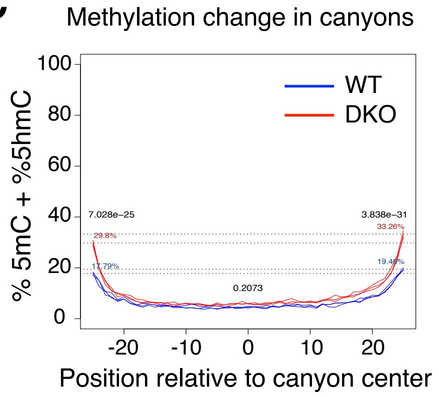

d

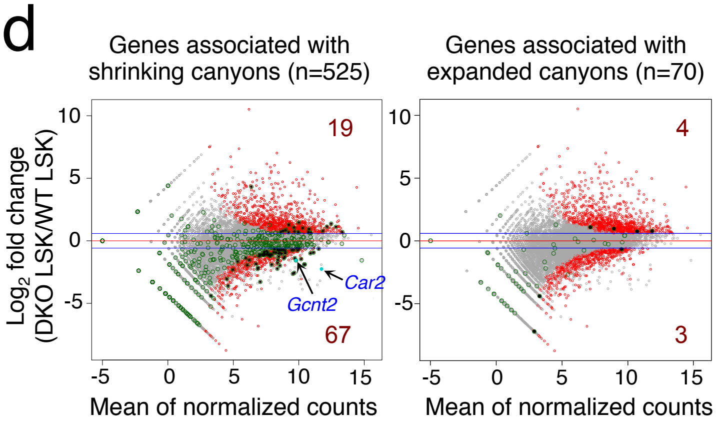

e

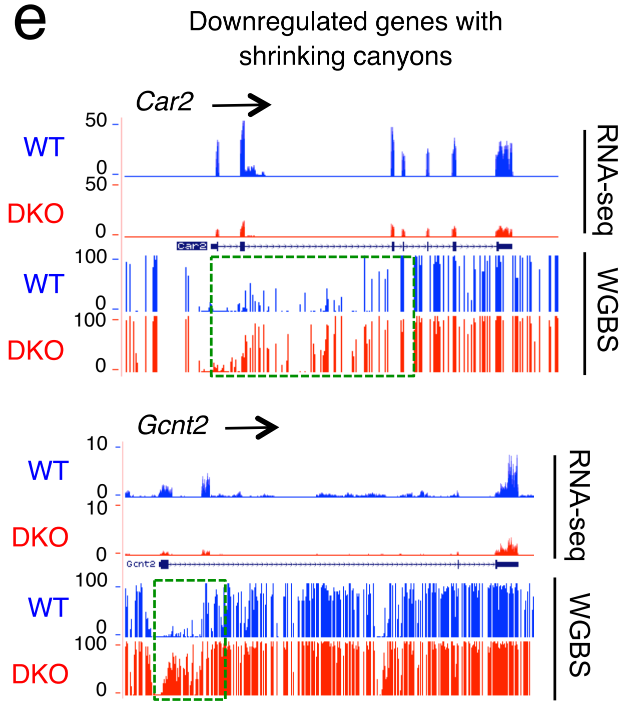

Supplementary Figure 18. DNA methylation at CpG islands and CGI shores and differentially methylated regions (DMRs).

Supplementary Figure 18. DNA methylation at CpG islands and CGI shores and differentially methylated regions (DMRs).

a, *Left panel*, Summary of the number of CpG Islands (CGIs) and CGI shores within or at the promoter regions (± 2 kb) of genes that show altered methylation in WT compared to *Tet2/3* DKO LSK cells. *Right panel*, lack of a clear relation between the DNA modification change in CGI shores at promoters (n=12,005) and the change in expression level of the associated genes. The left, central and right sets of dots show CGI shores with decreased, unchanged or increased methylation (methylation differences < -10, -5 to +5 and >+10 respectively). Of 1539 CGI shores that show increased DNA modification, only 251 genes are differentially expressed (red dots), and of these, 116 genes are upregulated whereas 135 genes are downregulated.

b, Summary of the number of canyons in WT LSK cells, including the number of canyons that shrink or expand in *Tet2/3* DKO LSK cells compared to WT.

c, Average DNA methylation at canyons that decrease in length in *Tet2/3* DKO LSK compared to WT LSK cells. The plot illustrates the overall absence of DNA methylation in the canyon center and the increase of DNA methylation at the canyon borders in *Tet2/3* DKO LSK compared to control LSK cells. There is a tendency for the gain of methylation in *Tet2/3* DKO LSK cells to be more pronounced at the right side of figure, which corresponds to the canyon border towards or within the downstream promoter region of the nearest gene.

d, The figure shows the MA plot of Fig. 5a, with the red dots indicating all genes differentially expressed between WT and *Tet2/3* DKO LSK cells, and the green dots the genes associated with canyons that shrink (*left panel; n=525*) or expand (*right panel; n=70*) in *Tet2/3* DKO LSK cells versus WT LSK cells. *Black dots*, genes that are both differentially expressed and associated with canyons that shrink or expand. Genes associated with shrinking canyons are biased towards downregulation in *Tet2/3* DKO LSK cells compared to WT.

e, Examples of RNA-seq (*top*) and WGBS (*bottom*) results for two downregulated genes (*Car2* and *Gcnt2*) associated with canyons that shrink in *Tet2/3* DKO LSK (*red*) compared with WT LSK (*blue*) cells.

Supplementary Fig. 19

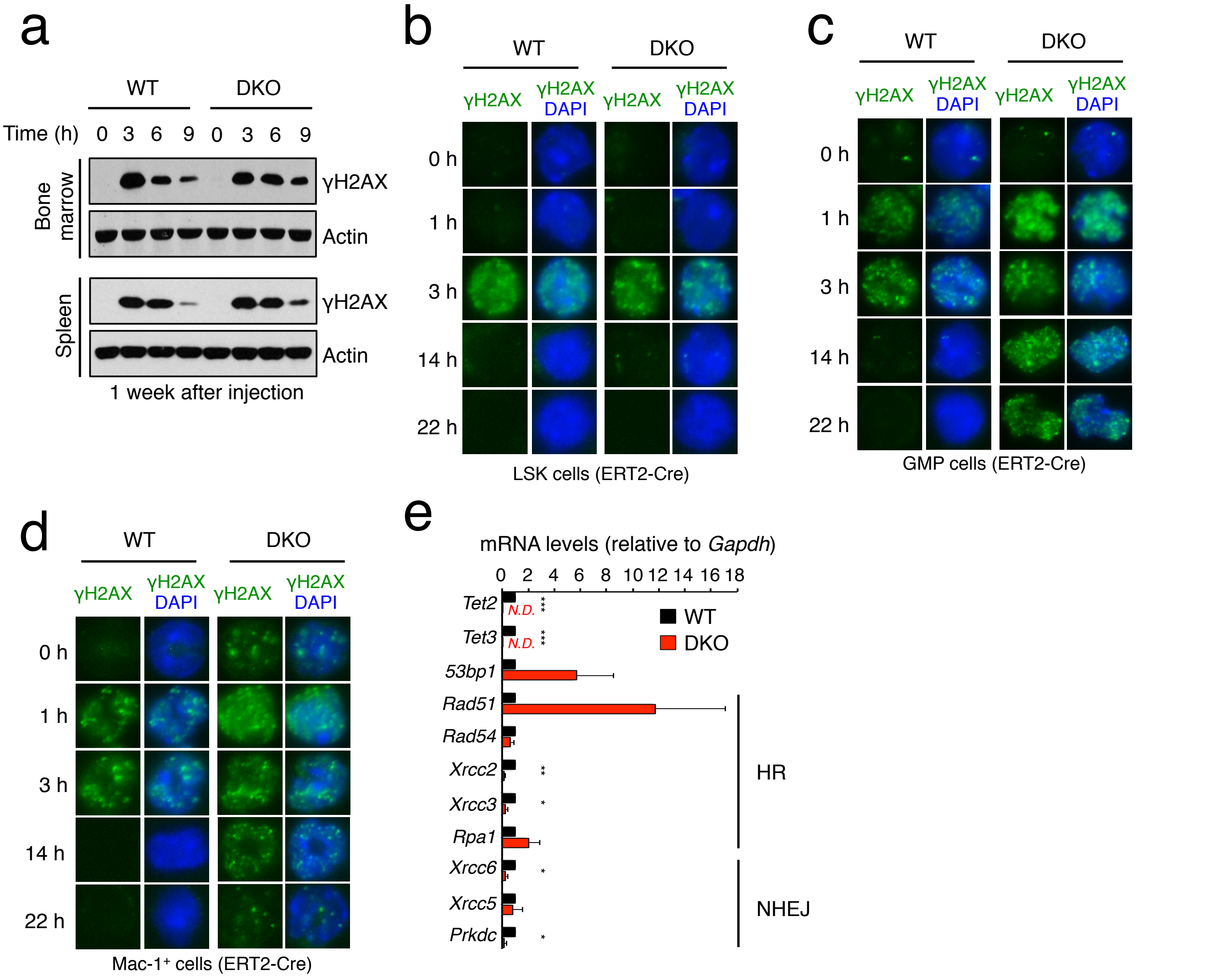

Supplementary Figure 19. Loss of Tet2 and Tet3 results in accumulation of DNA damage and impaired DNA repair.

(a) Combined loss of Tet2 and Tet3 leads to accumulation of γH2AX. Control and *Tet2/3* DKO mice were irradiated (6 Gy) at 1 week after pIpC injection and bone marrow (*top panel*) or spleen (*bottom panel*) were harvested at the indicated time points. Whole cell lysates were prepared and analyzed for the expression of γH2AX. Actin serves as a loading control.

(b) Efficient DNA repair in DKO LSK cells. LSK cells were isolated from control or DKO mice ($Tet2^{-/-}$ $Tet3^{fl/fl}$ $ERT2\text{-}Cre^{+}$) at 3 weeks after pIpC or tamoxifen injection, respectively, and DNA repair kinetics in response to 6 Gy of ionizing radiation were assessed by immunocytochemistry.

(c,d) DNA damage repair is impaired in myeloid lineage cells upon loss of Tet2 and Tet3. GMP (**c**) or Mac-1^{+} cell (**d**) were sorted from the bone marrow of control and $Tet2^{-/-}$ $Tet3^{fl/fl}$ $ERT2\text{-}Cre^{+}$ DKO mice at 3 weeks after tamoxifen injection and DNA repair kinetics in response to 6 Gy of ionizing radiation were assessed by immunocytochemistry.

(**e**) TET proteins control the expression of DNA repair genes in myeloid cells. Mac-1^{+} cells were sorted from the bone marrow of WT ($Tet2^{+/+}$ $Tet3^{fl/fl}$) and DKO ($Tet2^{-/-}$ $Tet3^{fl/fl}$ $Mx1\text{-}Cre^{+}$) mice at 3 ~ 4 weeks after pIpC injection, and quantitative RT-PCR (PCR with reverse transcription) was performed to assess the expression of genes implicated in homologous recombination (HR) and non-homologous end-joining (NHEJ). Results are expressed as fold change compared with WT cells (arbitrarily set to 1). Data from three independent experiments are shown (Means and SEM). *N.D.*, not detected. $^{*}P < 0.05$, $^{**}P < 0.005$, $^{***}P < 0.0005$ (Student's *t* test).

Supplementary Fig. 20

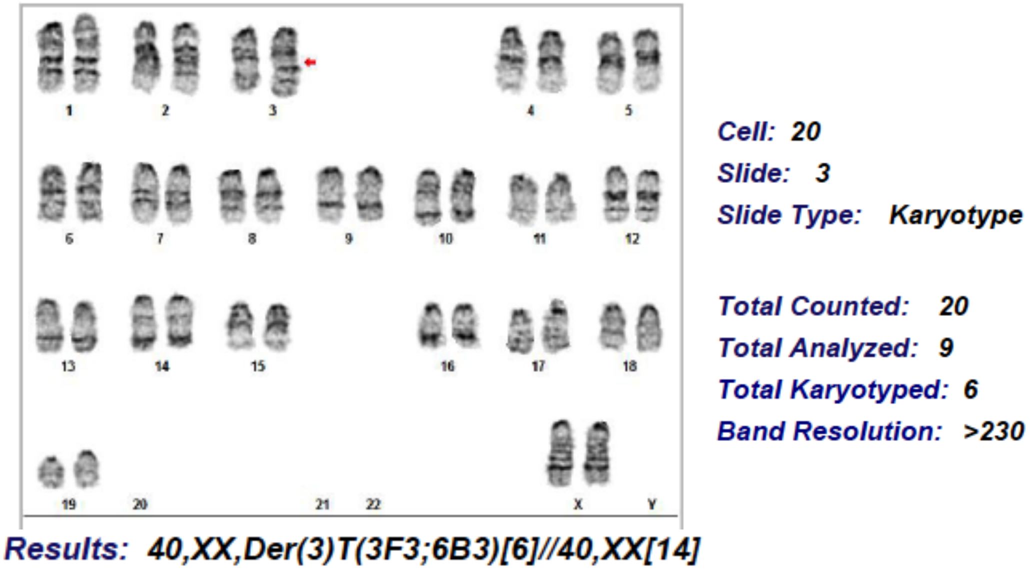

Supplementary Figure 20. Chromosomal aberrations in bone marrow cells from *Tet2/3* DKO mice. Red cell-depleted bone marrow cells were isolated from WT mice and sick *Tet2/3*-deficient DKO mice and subjected to G-banded karyotyping. In one of four bone marrow samples, 6/20 DKO cells examined displayed a structural abnormality of chromosome 3 – an unbalanced translocation of distal chromosome 6 to distal chromosome 3, resulting in an extra copy of the translocated sequences of chromosome 6 and a loss of copy of chromosome 3 distal to the breakpoint on 3.

Supplementary Fig. 21

Original blots for the main Fig. 6a,b,c, h and Supplementary Fig. 19a

Fig. 6a

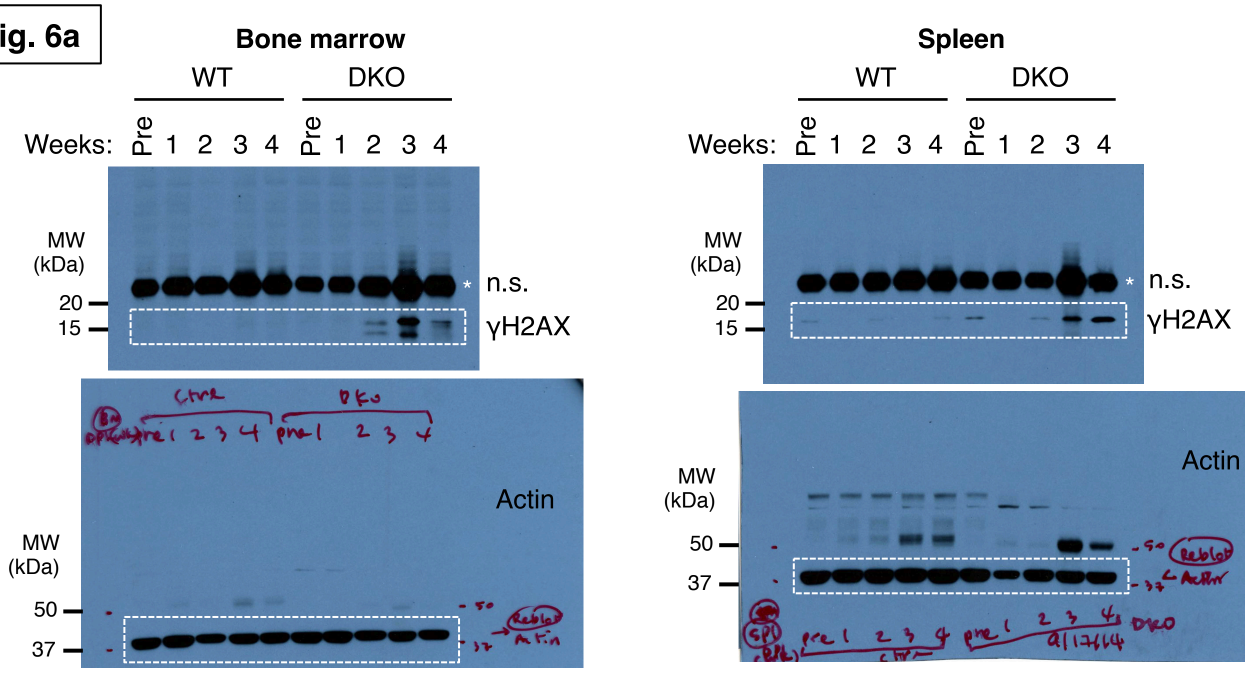

Fig. 6b

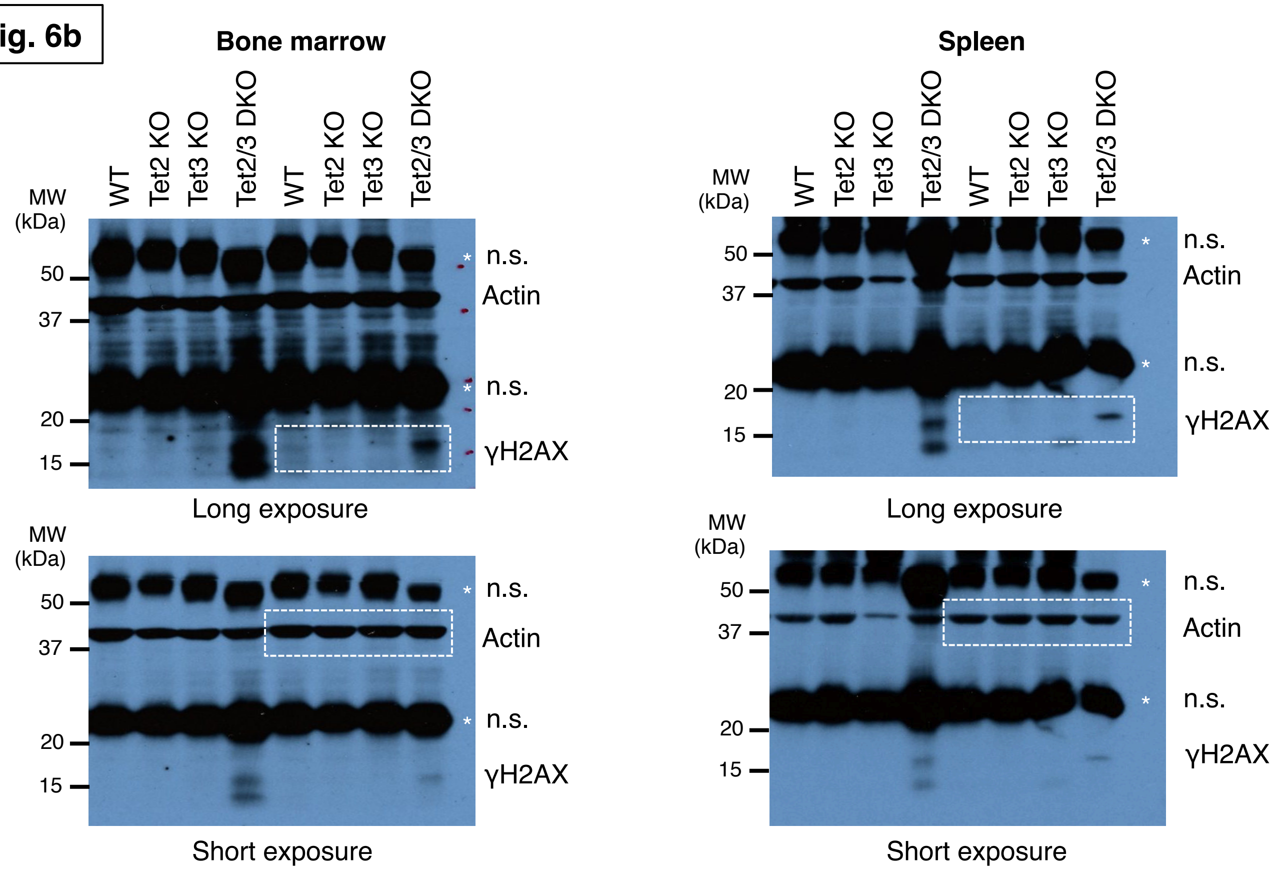

Fig. 6c

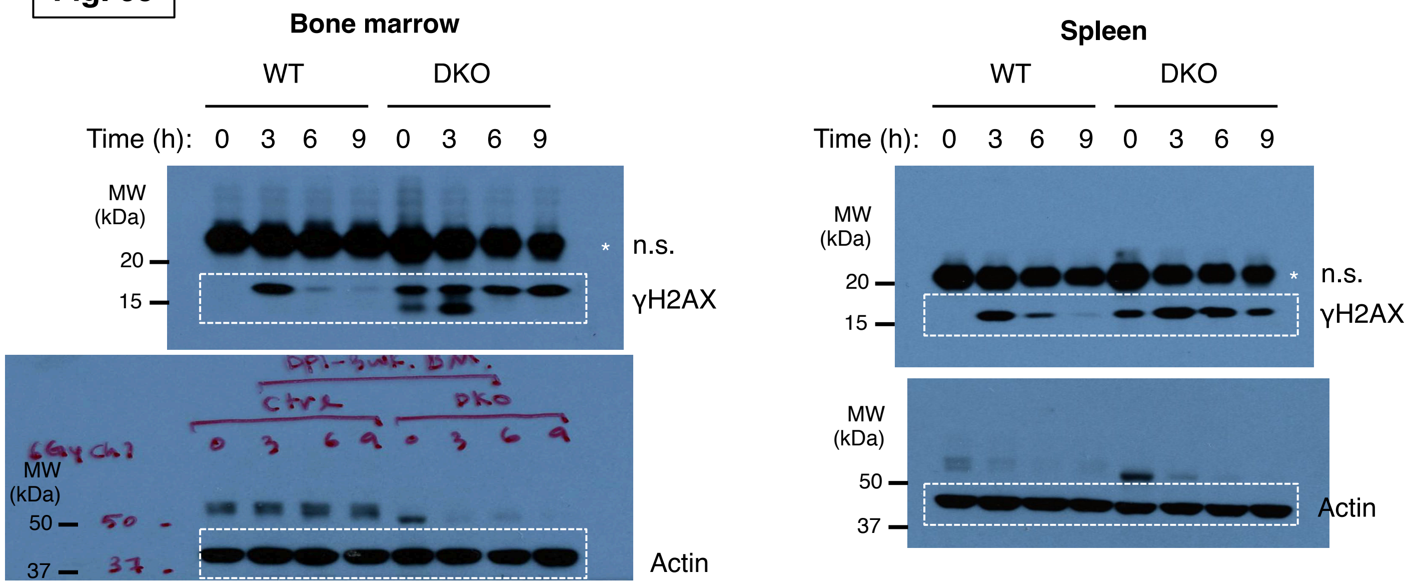

Suppl. Fig. 19a

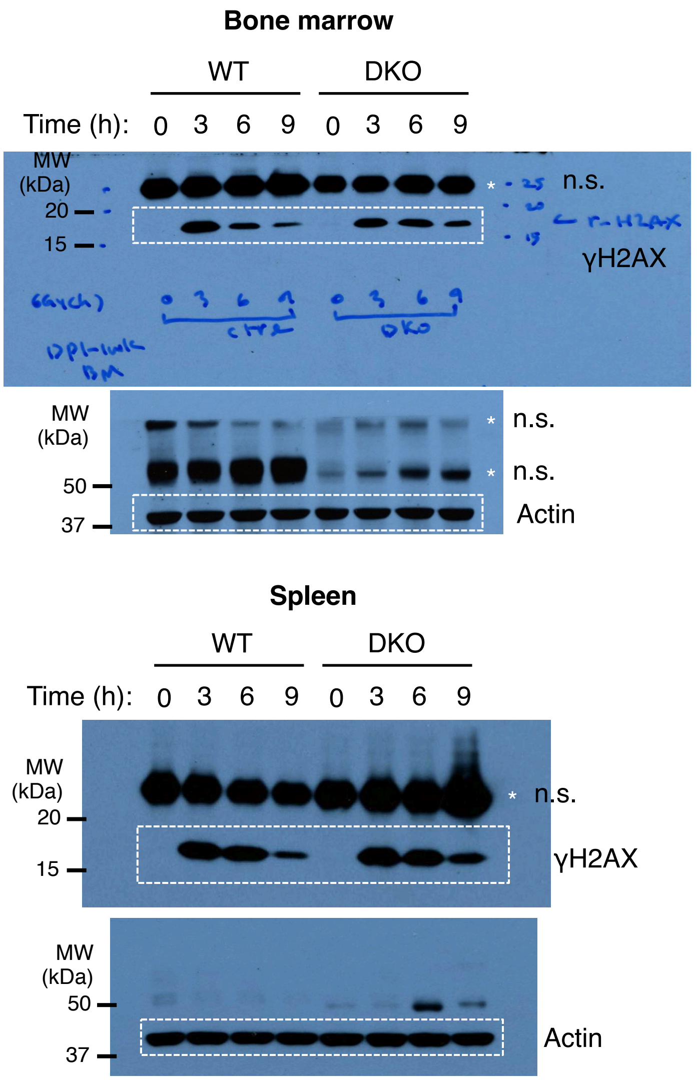

Fig. 6h

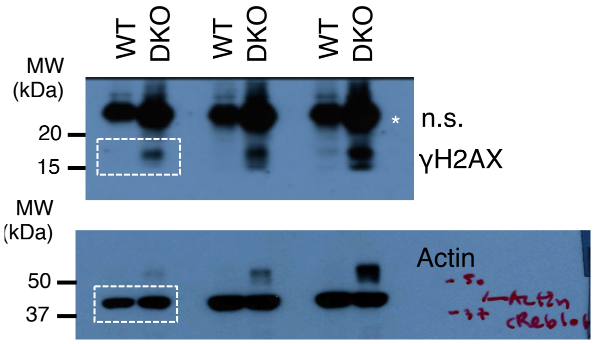

Supplementary Tables

Supplementary Table 1. Long-term monitoring of health status of WT, Tet2-deficient and Tet3-deficient mice.

WT	Genotype	Days post injection (DPI)	Health status
1	Tet3fl/fl	15 months	healthy
2	Tet3fl/fl	15 months	healthy
3	Tet3fl/fl	15 months	healthy
4	Tet3fl/fl	15 months	healthy
5	Tet3fl/fl	15 months	healthy
6	Tet3fl/fl	14.5 months	healthy
7	Tet3fl/fl	14.5 months	healthy
8	Tet3fl/fl	14.5 months	healthy
9	Tet3fl/fl	12.8 months	healthy
10	Tet2fl/fl	12.8 months	healthy
11	Tet3fl/+	12.8 months	healthy
12	Tet3fl/+	12.8 months	healthy
13	Tet3fl/fl	12.8 months	healthy
14	Tet3fl/fl	10.7 months	healthy
15	Tet3fl/fl	10.7 months	healthy
16	Tet3fl/fl	10.7 months	healthy
17	Tet3fl/fl	10.7 months	healthy
18	Tet3fl/fl	8.4 months	healthy
19	Tet3fl/fl	7.3 months	healthy
20	Tet3fl/fl	7.3 months	healthy
21	Tet3fl/fl	7.3 months	healthy

T2KO	Genotype	Days post injection (DPI)	Health status
1	Tet2-/- Tet3fl/fl	15 months	healthy
2	Tet2-/- Tet3fl/fl	12.2 months	Found dead
3	Tet2-/- Mx1-Cre	13.2 months	Found dead
4	Tet2-/- Tet3fl/fl	10.7 months	healthy
5	Tet2-/- Tet3fl/fl	10.7 months	healthy
6	Tet2-/- Mx1-Cre	10.7 months	healthy
7	Tet2-/- Mx1-Cre	10.7 months	healthy
8	Tet2-/- Tet3fl/fl	10.7 months	healthy
9	Tet2-/- Tet3fl/fl	8.4 months	healthy
10	Tet2-/- Mx1-Cre	8.4 months	healthy
11	Tet2-/- Tet3fl/fl	7.3 months	healthy
12	Tet2-/- Tet3fl/fl	7.3 months	healthy

T3KO	Genotype	Days post injection (DPI)	Health status
1	Tet3fl/fl Mx1-Cre	15 months	healthy
2	Tet3fl/fl Mx1-Cre	15 months	healthy
3	Tet3fl/fl Mx1-Cre	15 months	healthy
4	Tet3fl/fl Mx1-Cre	15 months	healthy
5	Tet3fl/fl Mx1-Cre	14.5 months	healthy
6	Tet3fl/fl Mx1-Cre	10.7 months	healthy
7	Tet3fl/fl Mx1-Cre	10.7 months	healthy
8	Tet3fl/fl Mx1-Cre	10.7 months	healthy
9	Tet3fl/fl Mx1-Cre	10.7 months	healthy
10	Tet3fl/fl Mx1-Cre	10.7 months	healthy
11	Tet3fl/fl Mx1-Cre	10.7 months	healthy
12	Tet3fl/fl Mx1-Cre	10.7 months	healthy
13	Tet3fl/fl Mx1-Cre	7.3 months	healthy
14	Tet3fl/fl Mx1-Cre	7.3 months	healthy

Supplementary Tables

Supplementary Table 2. Hematopoietic parameters after cell transfer experiments

a. Haematopoietic parameters in recipients of splenocytes (*Tet2$^{-/-}$ Tet3$^{fl/fl}$ Mx1-Cre^{+}*).

Parameter	WT	DKO	P value	Abnormality
WBC (10^3/μl)	1.95±0.33	98.14±27.54	**, 0.0008	Leukocytosis
RBC (10^6/μl)	6.27±0.64	3.4±0.28	*, 0.0063	Anaemia
Hemoglobin (g/dL)	8.93±0.78	5.6±0.6	*, 0.0119	Anaemia
Hematocrit (%)	32.69±3.09	21.62±2.46	*, 0.0288	Anaemia
Platelet (10^3/μl)	450.25±41.67	240.8±34.63	*, 0.005	Thrombocytopenia
Lymphocyte (10^3/μl)	1.16±0.36	6.372±1.07	**, 0.0002	Lymphocytosis
Neutrophil (10^3/μl)	0.67±0.08	79.9±22.96	**, 0.0009	Neutrophilia
Monocyte (10^3/μl)	0.1±0.012	7.89±2.98	*, 0.0492	Monocytosis
Basophil (10^3/μl)	0.003±0.0016	0.54±0.23	*, 0.0112	Basophilia
BM cell number (X10^7)	5.15±0.54	2.59±0.43	*, 0.0059	
Spl cell number (X10^7)	9.92±1.27	31.56±0.35	***, <0.0001	

b. Haematopoietic parameters in recipients of splenocytes (*Tet2$^{fl/fl}$ Tet3$^{fl/fl}$ Mx1-Cre^{+}*).

Parameter	Control	DKO	P value	Abnormality
WBC (10^3/μl)	2.04±0.23	101.31±20.78	***, 0.0003	Leukocytosis
RBC (10^6/μl)	8.70±0.21	2.74±0.41	***, <0.0001	Anaemia
Hemoglobin (g/dL)	12.90±0.22	5.93±0.48	***, <0.0001	Anaemia
Hematocrit (%)	47.48±0.90	15.90±1.90	***, <0.0001	Anaemia
Platelet (10^3/μl)	410.00±37.16	643.75±174.42	0.1468	
Lymphocyte (10^3/μl)	0.97±0.10	37.01±4.7	***, <0.0001	Lymphocytosis
Neutrophil (10^3/μl)	0.92±0.11	44.65±19.16	*, 0.0203	Neutrophilia
Monocyte (10^3/μl)	0.11±0.02	15.43±3.03	**, 0.002	Monocytosis
Basophil (10^3/μl)	0.01±0.001	0.24±0.19	0.1618	
Eosinophil (10^3/μl)	0.04±0.02	3.99±2.70	0.1012	
BM cell number (X10^7)	3.69±0.31	7.32±0.11	**, 0.0054	
Spl cell number (X10^7)	2.50±0.43	87.60±32.18	*, 0.0102	

c. Haematopoietic parameters in recipients of bone marrow cells (*Tet2$^{fl/fl}$ Tet3$^{fl/fl}$ ERT2-Cre^{+}*).

Parameter	WT	DKO	P value	Abnormality
WBC (10^3/μl)	10.83±0.52	58.86±3.17	***, <0.0001	Leukocytosis
RBC (10^6/μl)	9.85±0.21	5.60±0.2	***, <0.0001	Anaemia
Hemoglobin (g/dL)	12.82±0.28	7.26±0.34	***, <0.0001	Anaemia
Hematocrit (%)	49.48±1.1	28.4±1.35	***, <0.0001	Anaemia
Platelet (10^3/μl)	394.4±36.35	445.6±38.49	0.3618	
Lymphocyte (10^3/μl)	7.5±0.36	6.27±1.48	0.4444	
Neutrophil (10^3/μl)	2.71±0.19	48.8±3.97	***, <0.0001	Neutrophilia
Monocyte (10^3/μl)	0.46±0.04	1.37±0.33	*, 0.0258	Monocytosis
Basophil (10^3/μl)	0.022±0.008	0.25±0.78	*, 0.0203	Basophilia
Eosinophil (10^3/μl)	0.14±0.034	2.16±0.26	***, <0.0001	Eosinophilia
BM cell number (X10^7)	4.64±0.158	1.66±0.143	***, <0.0001	
Spl cell number (X10^7)	7.28±0.484	23.84±6.59	*, 0.0365	

d. Haematopoietic parameters in recipients of Mac1^{+} cell cells (*Tet2$^{fl/fl}$ Tet3$^{fl/fl}$ Mx1-Cre^{+}*).

Parameter	WT	DKO	P value	Abnormality
WBC (10^3/μl)	9.39±1.11	93.57±11.64	***, <0.0001	Leukocytosis
RBC (10^6/μl)	9.95±0.13	3.11±0.73	***, <0.0001	Anaemia
Hemoglobin (g/dL)	13.94±0.19	6.15±1.08	***, <0.0001	Anaemia
Hematocrit (%)	47.40±1.49	18.80±3.84	***, <0.0001	Anaemia
Platelet (10^3/μl)	681.22±58.66	291.13±27.72	***, <0.0001	Thrombocytopenia
Lymphocyte (10^3/μl)	7.21±0.94	16.04±4.25	*, 0.0489	Lymphocytosis
Neutrophil (10^3/μl)	1.89±0.19	58.85±11.26	***, <0.0001	Neutrophilia
Monocyte (10^3/μl)	0.25±0.04	12.70±1.47	***, <0.0001	Monocytosis
Basophil (10^3/μl)	0.01±0.00	0.37±0.12	*, 0.0064	Basophilia
Eosinophil (10^3/μl)	0.03±0.01	5.60±1.46	**, 0.001	Eosinophilia
BM cell number (X10^7)	4.83±0.46	7.52±0.80	*, 0.0086	
Spl cell number (X10^7)	3.52±0.55	22.65±1.67	***, <0.0001	

Supplementary Tables

Suppl. Table 3. Mapping statistics of RNA-Seq analysis.

Library/Sample	Total mapping to low-complexity reads	Total mapping to low-complexity reads %	Total mapping to reference	Total mapping to reference %	Total uniquely mapping Reads	Total uniquely mapping Reads %	Total unmapped	Total unmapped %
WT no. 1	561,236	3.06%	14,850,440	78.12%	13,794,022	72.56%	2,443,862	13.33%
WT no. 2	870,973	5.09%	12,508,630	70.86%	11,499,846	65.15%	3,497,843	20.44%
DKO no. 1	391,230	2.32%	14,394,045	83.13%	13,336,318	77.02%	1,715,346	10.19%
DKO no. 2	420,776	2.27%	15,673,189	80.69%	14,289,249	73.57%	1,995,785	10.77%

Suppl. Table 4. Pearson correlation matrix showing high correlation between replicates (0.98~0.99).

	DKO. no. 1	DKO. no. 2	WT no. 1	WT no. 2
**DKO no. 1**	1	0.99	0.94	0.92
**DKO no. 2**		1	0.92	0.9
**WT no. 1**			1	0.98
**WT no. 2**				1

Suppl. Table 5. Mapping statistics of WGBS analysis and bisulfite conversion efficiency.

Library/Sample	Total Number of Reads	Total mapping to reference	Total mapping to reference (%)	Covered Base pairs	Genome Coverage	Genome coverage per condition	Bisulfite conversion efficiency
WT no. 1	215,810,374	197,201,728	91.38	20,114,576,256	7.38	**21.24**	0.9982
WT no. 2	192,188,864	177,428,451	92.32	18,097,702,002	6.64		0.9969
WT no. 3	208,921,452	192,865,002	92.31	19,672,230,204	7.22		0.9966
DKO no.1	217,675,274	201,809,500	92.71	20,584,569,000	7.55	**22.2**	0.9957
DKO no.2	230,150,082	211,497,316	91.9	21,572,726,232	7.91		0.9983
DKO no.3	193,437,008	179,920,048	93.01	18,351,844,896	6.73		0.9981

Supplementary Tables

Suppl. Table 6. Distribution of DMRs across genome.

All DMRs

Name	P-value
Promoters	0.00E+00
Exons	2.14E-56
Protein-coding	1.06E-52
Coding	5.07E-28
5'-UTR	1.05E-22
TTS	4.77E-19
3'-UTR	5.19E-15
miscRNA	2.05E-05
CpG islands	4.69E-05
ncRNA	5.48E-05
Introns	5.58E-02
miRNA	2.59E-01
snRNA	1.00E+00
snoRNA	1.00E+00
Unknown	9.88E-01
rRNA	9.29E-01
Pseudo	3.84E-01
Centromeres	3.20E-285
Gaps	0.00E+00
Intergenic	0.00E+00

Hypermethylated in DKO

Name	P-value
Promoters	2.99E-238
Exons	5.00E-44
Protein-coding	1.44E-40
Introns	2.35E-34
Coding	2.63E-22
3'-UTR	2.61E-17
TTS	1.26E-16
5'-UTR	5.67E-08
miscRNA	2.48E-05
ncRNA	5.53E-05
miRNA	4.73E-01
snRNA	1.00E+00
Unknown	9.92E-01
rRNA	9.53E-01
snoRNA	9.34E-01
Pseudo	4.05E-01
CpG islands	3.23E-02
Centromeres	6.04E-185
Gaps	2.12e-312
Intergenic	0.00E+00

Hypomethylated in DKO

Name	P-value
Promoters	2.11E-153
5'-UTR	9.56E-19
CpG islands	1.07E-15
Exons	1.33E-14
Protein-coding	3.41E-14
Coding	9.01E-08
TTS	1.70E-04
3'-UTR	8.10E-02
miscRNA	1.06E-01
ncRNA	1.33E-01
Pseudo	6.58E-01
snRNA	1.00E+00
Unknown	9.96E-01
snoRNA	1.00E+00
miRNA	1.00E+00
rRNA	9.75E-01
Introns	2.93E-46
Centromeres	1.08E-100
Gaps	7.41E-170
Intergenic	0.00E+00

Supplementary Methods

Histology

Mice were sacrificed and tissue biopsies were fixed overnight in buffered formalin (Fisher Scientific, SF93-4), dehydrated and embedded in paraffin according to standard laboratory protocol. Bones were fixed and decalcified prior to embedding in paraffin. Paraffin blocks were sectioned at 3 µm and stained with hematoxylin and eosin (H&E) For immunohistochemistry, specimens were deparaffinized and endogenous peroxidase was blocked in 0.3% H_2O_2, then incubated with anti-myeloperoxidase antibody (Abcam, ab9535) following heat-induced antigen retrieval. Preparation of paraffin blocks, H&E staining and immunohistochemistry were performed at the Histology Core at the University of California, San Diego.

Peripheral blood and bone marrow analysis

Blood was collected by retro-orbital bleeding into EDTA-treated Microvette (Fisher Scientific) and smeared on a slide, air-dried and stained using May-Grünwald Giemsa staining (Sigma) according to the manufacturer's instruction. Automated peripheral blood counts were obtained using a Hemavet 950 (Drew Scientific) according to manufacturer's instruction. Bone marrow smears were prepared by bone marrow brush preparation and stained with May-Grünwald Giemsa staining (Sigma). Images were obtained using BZ-9000 slide scanner system (Keyence, Osaka, Japan) or Nikon Eclipse 80i microscope (Nikon Instruments Inc.).

RNA-Sequencing (RNA-Seq)

We used sorted LSK cells for RNA-seq experiments because of the problems in accurately defining LT-HSCs in *Tet2/3* DKO mice (see **Supplementary Fig. 10**). Total RNA was extracted from two biological replicates each of WT and *Tet2/3* DKO LSK cells using Purelink RNA Micro kit (Invitrogen). Using 10 ng of total RNA, cDNA was synthesized and amplified using SMARTer® Ultra™ Low Input RNA for Illumina® Sequencing-HV (Clontech® Laboratories, Inc.). RNA-Seq libraries were constructed using Nextera® XT DNA Sample Preparation kit (Illumina) and single end sequencing was performed using the Illumina HiSeq 2500 on Rapid Run mode.

Analysis of RNA sequencing data

RNA-seq data was mapped against mm9 using TopHat[1] (v.1.4.1, default parameters except --no-coverage-search, --library-type=fr-unstranded) and the *RefSeq* gene annotation was obtained from the UCSC genome Bioinformatics data base (January 27, 2011). The number of reads mapping to each gene was counted using HTSeq (-m union, -s yes, -t exon, -i gene_id, http://www.huber.embl.de/users/anders/HTSeq/). To identify differential gene expression between cell-types, we performed negative binomial tests for pairwise comparisons of the Control and DKO LSK cells by employing the Bioconductor package *DESeq*[2] using a p-value ≤ 0.05 and fold change thresholds of > 1.5 or < 0.67. Library size per sample was calculated as the sum of DESeq normalized counts across all genes and was used for calculating RPKM values. The Pearson correlation between biological replicates is 0.98 and 0.99 (**Supplementary Table 4**) indicating high reproducibility of replicates, whereas the Pearson correlations between conditions range from 0.89 to 0.94. For the heat maps, row-wise scaled RPKM values across all samples were plotted using the function *heatmap.2* in the R package *gplots* (www.r-project.org).

Gene set enrichment analysis

To test if selected gene sets are enriched in *Tet2/3* DKO LSK compared to control LSK cells, we applied Gene Set Enrichment Analysis (GSEA)[3] to our processed RNA-seq data (RPKM values of all genes) using signal to noise to rank gene expression values and 1000 permutations to test enrichment. Gene sets used in this study were obtained from references 4, 5, 6 and 7.

Whole genome bisulfite sequencing (WGBS)

Whole-genome bisulfite sequencing was performed on three biological replicates each of WT and *Tet2/3* DKO LSK cells as described previously with minor modifications[8]. For WGBS library construction, genomic DNA was fragmented using a Covaris sonication system (Covaris S2). DNA libraries were constructed using the Illumina TruSeq Nano DNA sample preparation kit. Adapter ligated libraries were bisulfite-treated using the EpiTect Bisulfite Kit (Qiagen, Valencia, CA). Enrichment PCR was done using TrueSeq primer mix and *Pfu* TurboCx hotstart DNA polymerase (Stratagene). DNA libraries were sequenced on Illumina HiSeq 2000 sequencing systems.

WGBS data analysis

Analysis of non-CpG methylation confirmed that bisulfite conversion efficiencies were higher than 99.57% in all samples **(Supplementary Table 5)**. A total of 617 and 641 million reads were generated for WT and *Tet2/3* DKO LSK, respectively, of which 92% and 92.54% aligned successfully to the reference mm9 genome **(Supplementary Table 5)**. The combined genome coverage was 21x and 22x for WT LSK and *Tet2/3* DKO LSK cells, respectively **(Supplementary Table 5)**. For downstream analyses, we removed multiple mappers and corrected for PCR amplifications and methylation bias due to 5' end repair (*see Methods*). We employed BSMAP v2.74[9] to align paired-end reads from bisulfite-treated samples to the *mm9* mouse reference genome (-R -p 12 -n 1 -v 4 -w 2 -r 1 -q 20). Low quality 5' ends of sequencing reads were trimmed by BSMAP. Reads mapping to multiple locations in the reference genome with the same mapping score were removed. Duplicated reads caused by PCR amplification were removed by BSeQC v1.0.3[10] applying a Poisson P-value cutoff of 10^{-5}. Consequently, a maximum of three stacked reads at the same genomic location were allowed and kept for further analysis. In addition, BSeQC[10] was employed for removing DNA methylation artifacts introduced by end repair during adaptor ligation. Overlapping segments of two mates of a pair were reduced to only one copy to avoid considering the same region twice during the subsequent DNA methylation quantification. To estimate CpG DNA methylation at both DNA strands, we executed the *methratio.py* script of BSMAP v2.74[9] (-t 0 -g no -x CG -i correct).

To identify differentially "methylated" regions (DMRs), we employed BSseq v1.2.0 under an R session (v3.1.0) to obtain DMRs between WT and TET2/3 DKO LSK cells, using CpGs covered at least 2 times in all samples per group (6 out of 6 total samples per comparison). After calculating t test statistics in all the CpGs overcoming the aforementioned threshold, we kept only 2% of the most significant CpG comparisons to compute DMRs (defined as a genome region having a total methylation change of at least 10% in at least 5 CpGs separated no more than 500bp from one another).

To calculate average DNA methylation at gene regions, we divided the gene bodies into 100 bins, regardless

of actual gene size, and divided the 2 kb region upstream of the transcription start sites (TSS) and downstream of the transcription termination site (TTS) into 20 bins each. For each gene and for each bin, we calculated the mean methylation of all CpGs falling into the bins. To calculate average methylation profiles across genes, we stacked the genes of interest and calculated mean methylation at each of the bins across all genes.

To identify DNA methylation canyons, we pooled replicates per condition and applied the *mOne* module of the *MOABS*[11] package (v1.2.2) using default parameters settings. Undermethylated regions (UMRs) are required to have a methylation level lower than 10% with at least five CpGs per kilobase and a permutation-based false discovery rate (FDR) of 5%. UMRs of at least 3.5 kb length are considered as canyons[8].

For the contour plot of the methylation percentages (5mC + 5hmC) in WT and *Tet2/3* DKO LSK cells (**Fig. 5f**), we examined the enhancer regions identified in LT-HSC by Lara-Astiaso et al.[12] based on overlapping H3K4me1 and H3K4me2 peaks and low H3K4me3 levels. Enhancer activity was assigned based on a threshold of H3K27Ac level (normalized number of reads), as described in Lara-Astiaso et al.[12]. Only those CpGs covered at least 5x in merged WT as well as DKO biological replicates were considered (total of 123,466 CpGs across 7033 active enhancers). The enhancer regions were extended by 1 kb in each direction from the peak centre and divided into 50 bins per axis so that each bin represents 2% 5mC + 5hmC increments in the y-axis and 40 bp in the x-axis, then re-centred based on the position of the maximum value from the ATAC-seq coverage profile. CpG density was uniformly distributed along the enhancer regions (i.e there was essentially no positional bias; **Fig. 5f**, *top*).

Dot blot analysis

Dot blot assays were performed as described elsewhere[13]. Briefly, cells were incubated with 200 µg/ml proteinase K (Roche) overnight at 55°C and genomic DNA was isolated by phenol-chloroform extraction. For CMS detection, genomic DNAs were treated with sodium bisulfite using the EpiTect Bisulfite kit (QIAGEN). DNA samples were denatured in 0.4 M NaOH, 10 mM EDTA at 95ºC for 10 min, followed by neutralization with cold 2 M ammonium acetate (pH 7.0). Two-fold serial dilutions of the denatured DNA samples were spotted on a nitrocellulose membrane in an assembled Bio-Dot apparatus (Bio-Rad) according to the manufacturer's instructions. A synthetic oligonucleotide with a known amount of CMS was used as standard[13]. The membrane was washed with 2x SSC buffer, air-dried and vacuum-baked at 80°C for 2 hr, then blocked with 5% non-fat milk for 1 hr and incubated with anti-5hmC (1:5000, produced in-house) or anti-CMS antiserum (1:10,000, produced in-house) overnight at 4°C. After incubating with horseradish peroxidase-conjugated anti-rabbit IgG secondary antibody, the membrane was visualized by enhanced chemiluminescence (PerkinElmer). To ensure equal loading of total DNA on the membrane, the same blot was stained with 0.02% methylene blue in 0.3 M sodium acetate (pH 5.2).

Flow cytometry and cell sorting

Antibody staining and flow cytometric analysis was performed as described elsewhere[14]. Briefly, bone marrow cells were flushed out of femurs and tibiae in Hanks' balanced salt solution (HBSS, Gibco Invitrogen) containing 2% FBS and 10 mM HEPES (Gibco Invitrogen). Splenocytes and liver cells were prepared by mincing spleen and liver, respectively, onto a 70 µm cell strainer (BD Biosciences). Red blood cells were

depleted of liver cells prior to flow cytometry. Cells were stained with monoclonal antibodies in PBS containing 0.5% BSA or 1% heat-inactivated FBS and 0.1% (w/v) sodium azide. All antibodies were purchased from Biolegend, eBiosciences or BD Biosciences. The following monoclonal antibodies conjugated with fluorescein isothiocyanate (FITC), Alexa Fluor 488, phycoerythrin (PE), PerCP-Cy5.5, PE-Cy7, allophycocyanin (APC), eFluor 660, APC-Cy7, Alexa Fluor 700, Pacific Blue were used: c-Kit/CD117 (2B8), Sca-1 (D7), CD34 (RAM34), Flt-3/CD135/Flk-2 (A2F10.1), CD150 (TC15-12F12.2), CD48 (HM48-1), Ter-119 (Ter-119), CD3 (145-2C11), CD19 (ID3), B220/CD45R (RA3-6B2), Mac-1/CD11b (M1/70), Gr-1 (RB6-8C5), CD4 (GK1.5), CD8α (53-6.7), FcγRII/III (2.4G2, CD16/32), CD27 (LG.7F9), CD43 (S7), CD71 (RI7217), IL-7Rα/CD127 (A7R34), IgM (RMM-1), CD45.1 (A20) and CD45.2 (104). As a lineage cocktail, biotinylated antibodies against CD3ε (145-2C11), CD45R/B220 (RA3-6B2), Mac-1/CD11b (M1/70), Gr-1 (RB6-8C5), Ter-119 (Ter-119) (all from Mouse hematopoietic Lineage Biotin Panel, eBiosciences) and IL-7Rα (A7R34, Biolegend) were used in combination with a Streptavidin-PerCP-Cy5.5 conjugate (Biolegend). 7-AAD or Live/Dead Yellow (Invitrogen) was used to exclude dead cells. Flow cytometric analyses were performed using FACS Canto II (BD Biosciences) and data were analyzed using FlowJo software (Treestar). Mac-1$^+$ cells were isolated from unfractionated bone marrow or spleen cells. Prior to sorting of LSK and myeloid progenitor cells in the bone marrow, we depleted lineage positive cells as follows. Red cell-depleted total bone marrow cells were incubated with Biotin-conjugated Mouse Hematopoietic Lineage Panel (eBioscience) at 4°C for 20 min. After washing with 1X PBS containing 1% FBS, cells were incubated with Dynabeads® Biotin Binder (Life Technologies) at 4°C for 30 min and Lin$^-$ cells were purified by magnetic separation, followed by sorting using FACSAria II. For side population staining, bone marrow cells isolated from femurs and tibias were resuspended at 10^6 cells/ ml in pre-warmed DMEM containing 2% FBS, 10 mM HEPES and 5 µg/ml Hoechst 33342 (Sigma) and were incubated for 90 min at 37oC. After centrifugation, cells were resuspended in cold HBSS containing 2% FBS, 10 mM HEPES and 2 µg/ml propidium iodide, followed by flow cytometry.

Real-time RT-PCR

Total RNA was prepared with TRIzol reagent (Invitrogen) per manufacturer's instructions, followed by reverse transcription using SuperScript III (Invitrogen). Diluted cDNAs were analyzed by real-time PCR using StepONE plus real-time PCR system (Applied Biosystems) and FastStart Universal SYBR Green Master kit (Roche). Data were analyzed by StepONE plus real-time PCR software. The level of gene expression was normalized to *Gapdh*. Primer sequences are as follows: *Tet1*-forward: 5'-GAGCCTGTTCCTCGATGTGG-3', *Tet1*-reverse: 5'-CAAACCCACCTGAGGCTGTT-3'; *Tet2*-forward: 5'-AACCTGGCTACTGTCATTGCTCCA-3', *Tet2*-reverse: 5'-ATGTTCTGCTGGTCTCTGTGGGAA-3'; *Tet3*-foward: 5'-CAACCCTCAGTGGCTTCTTG-3', *Tet3*-reverse: 5'-TGGGCCTTCATCTTTCTCCA-3'; *Gapdh*-forward: 5'-GTGTTCCTACCCCCAATGTGT-3', *Gapdh*-reverse: 5'-ATTGTCATACCAGGAAATGAGCTT-3'. The sequences of primers for DNA repair genes in **Fig. 6i** and **Supplementary Fig. 19e** have been described previously[15].

Western blotting

Cells were lysed with RIPA buffer (150 mM NaCl, 50 mM Tris-HCl, pH 8.0, 1% Triton X-100, 0.5% sodium deoxycholate and 0.1% SDS) supplemented with protease inhibitor cocktail (Roche) or mixture of protease/ phosphatase inhibitors (20 mM β-glycerophosphate, 10 mM sodium pyrophosphate, 1 mM sodium o-vanadate,

10 μM leupeptin, 10 μg/ml aprotinin, 1 mM freshly prepared PMSF) and incubated on ice for 20 min. Cell debris was removed by centrifuging at 12,000 rpm for 15 min at 4°C. The protein concentration was measured by Bradford protein assay. Samples were mixed with SDS sample buffer and boiled for 4 min. Whole cell lysates were separated by 7.5% or 10% SDS-PAGE and transferred onto nitrocellulose membranes. Proteins were detected by immunoblotting in TBST (150 mM NaCl, 10 mM Tris-Cl, pH 8.0, 0.5% Tween-20) containing 5% low-fat milk and antibodies against γH2AX (Millipore 05-636) and Actin (Sigma A5441), followed by incubation with HRP-conjugated secondary antibodies (goat anti-mouse IgG HRP, Sigma) and enhanced chemiluminescence (PerkinElmer). For the kinetic analyses in **Fig. 6c** and **Supplementary Fig. 19a**, WT and DKO mice were irradiated at 6Gy, then sacrificed at different times thereafter, followed by preparation of whole cell lysates from bone marrow or spleen and immunoblotting. Full blot images for **Fig. 6a, b, c, h** and **Supplementary Fig. 19a** are shown in **Supplementary Fig. 21**.

Immunocytochemistry

Cells were sorted by flow cytometry and irradiated using RS2000 Biological Irradiator (Rad Source Technologies, Inc.), then cultured for different times (as shown in **Fig. 6d-g** or **Supplementary Fig. 19b-d**) on poly-L-lysine coated slides (Corning) in Iscove's modified Dulbecco's medium (IMDM) supplemented with 10% FBS, 100 U/ml penicillin, 100 U/ml streptomycin, 50 μg/ml β-mercaptoethanol, 1% Glutamax (GIBCO), 1X non-essential amino acid (GIBCO) and 1% sodium pyruvate (GIBCO)) supplemented with 50 ng/ml SCF, 10 ng/ml IL-3, 10 ng/ml IL-6, and 10 ng/ml TPO (all from Peprotech). For the culture of GMP and Mac-1^{+} cells, 10 ng/ml GM-CSF was additionally added. After this incubation, the cells were fixed onto the slides with 4% paraformaldehyde in PBS for 15 min at room temperature (RT), washed with PBS and permeabilized with 0.15% Triton X-100 in PBS for 15 min at RT. After washing with PBS, cells were blocked with 1% BSA, 0.05% Tween-20 in PBS overnight at 4°C. Then, slides were incubated in blocking buffer containing mouse anti-γH2AX antibody (Millipore 05-636) for 1.5 to 3 hr at RT. After extensive washing with PBS, Alexa Fluor 488-conjugated anti-mouse IgG (Invitrogen) were added in blocking buffer for 1 hr in the dark. After extensive washing, slides were mounted in SlowFade Gold antifade reagent containing DAPI (Molecular Probes, Eugene, OR). Images were obtained with Zeiss Axiovert 200 microscope using OpenLab imaging software (Improvision, Coventry, UK).

G-banded karyotyping

G-banded karyotyping was performed at Wi-Cell (Wisconsin, USA). Briefly, cell harvest, slide preparation and G-banded karyotyping were performed using standard cytogenetics protocols for cells in suspension. Cells were incubated with colcemid and then placed in hypotonic solution, followed by fixation. Metaphase cell preparations were stained with Leishman's stain.

Supplementary References

1. Trapnell, C. *et al.* Differential gene and transcript expression analysis of RNA-seq experiments with TopHat and Cufflinks. *Nature protocols* **7**, 562-78 (2012).
2. Anders, S. & Huber, W. Differential expression analysis for sequence count data. *Genome biology* **11**, R106 (2010).
3. Subramanian, A. *et al.* Gene set enrichment analysis: a knowledge-based approach for interpreting genome-wide expression profiles. *Proceedings of the National Academy of Sciences of the United States of America* **102**, 15545-50 (2005).
4. Klinakis, A. *et al.* A novel tumour-suppressor function for the Notch pathway in myeloid leukaemia. *Nature* **473**, 230-3 (2011).
5. Ng, S.Y., Yoshida, T., Zhang, J. & Georgopoulos, K. Genome-wide lineage-specific transcriptional networks underscore Ikaros-dependent lymphoid priming in hematopoietic stem cells. *Immunity* **30**, 493-507 (2009).
6. Ivanova, N.B. *et al.* A stem cell molecular signature. *Science* **298**, 601-4 (2002).
7. Sanjuan-Pla, A. *et al.* Platelet-biased stem cells reside at the apex of the haematopoietic stem-cell hierarchy. *Nature* **502**, 232-6 (2013).
8. Jeong, M. *et al.* Large conserved domains of low DNA methylation maintained by Dnmt3a. *Nature genetics* **46**, 17-23 (2014).
9. Xi, Y. & Li, W. BSMAP: whole genome bisulfite sequence MAPping program. *BMC bioinformatics* **10**, 232 (2009).
10. Lin, X. *et al.* BSeQC: quality control of bisulfite sequencing experiments. *Bioinformatics* **29**, 3227-9 (2013).
11. Sun, D. *et al.* MOABS: model based analysis of bisulfite sequencing data. *Genome biology* **15**, R38 (2014).
12. Lara-Astiaso, D. *et al.* Immunogenetics. Chromatin state dynamics during blood formation. *Science* **345**, 943-9 (2014).
13. Ko, M. *et al.* Impaired hydroxylation of 5-methylcytosine in myeloid cancers with mutant TET2. *Nature* **468**, 839-43 (2010).
14. Ko, M. *et al.* Ten-Eleven-Translocation 2 (TET2) negatively regulates homeostasis and differentiation of hematopoietic stem cells in mice. *Proceedings of the National Academy of Sciences of the United States of America* **108**, 14566-71 (2011).
15. Flach, J. *et al.* Replication stress is a potent driver of functional decline in ageing haematopoietic stem cells. *Nature* **512**, 198-202 (2014).
